# Antipsychotics and the QTc Interval During Delirium in the Intensive Care Unit: A Secondary Analysis of a Randomized Clinical Trial

**DOI:** 10.1001/jamanetworkopen.2023.52034

**Published:** 2024-01-22

**Authors:** Joanna L. Stollings, Christina S. Boncyk, Caroline I. Birdrow, Wencong Chen, Rameela Raman, Deepak K. Gupta, Dan M. Roden, Erika L. Rivera, Amelia W. Maiga, Shayan Rakhit, Pratik P. Pandharipande, E. Wesley Ely, Timothy D. Girard, Mayur B. Patel

**Affiliations:** 1 Critical Illness, Brain Dysfunction, and Survivorship Center, Vanderbilt University Medical Center, Nashville, Tennessee; 2 Department of Pharmaceutical Services, Vanderbilt University Medical Center, Nashville, Tennessee; 3 Department of Anesthesiology, Vanderbilt University Medical Center, Nashville, Tennessee; 4 Department of Biostatistics, Vanderbilt University Medical Center, Nashville, Tennessee; 5 Center for Health Services Research, Vanderbilt University Medical Center, Nashville, Tennessee; 6 Vanderbilt Heart Imaging Core Lab, Vanderbilt Translational and Clinical Cardiovascular Research Center, Department of Medicine, Vanderbilt University Medical Center, Nashville, Tennessee; 7 Department of Medicine, Departments of Pharmacology and Biomedical Informatics, Vanderbilt University School of Medicine, Nashville, Tennessee; 8 Section of Surgical Sciences, Department of Surgery, Vanderbilt University Medical Center, Nashville, Tennessee; 9 Surgical Service, Department of Veterans Affairs Medical Center, Tennessee Valley Healthcare System, Nashville, Tennessee; 10 Anesthesia Service, Veterans Affairs Medical Center, Tennessee Valley Healthcare System, Nashville; 11 Geriatric Research Education and Clinical Center, Veterans Affairs Medical Center, Tennessee Valley Healthcare System, Nashville; 12 Department of Medicine, Vanderbilt University Medical Center, Nashville, Tennessee; 13 Center for Research, Investigation, and Systems Modeling of Acute Illness in the Department of Critical Care Medicine, University of Pittsburgh School of Medicine, Pittsburgh, Pennsylvania

## Abstract

**Importance:**

Antipsychotic medications, often prescribed for delirium in intensive care units (ICUs), may contribute to QTc interval prolongation.

**Objective:**

To determine whether antipsychotics increase the QTc interval in patients with delirium in the ICU.

**Design, Setting, and Participants:**

An a priori analysis of a randomized clinical trial in medical/surgical ICUs within 16 centers across the US was conducted. Participants included adults with delirium in the ICU with baseline QTc interval less than 550 ms. The study was conducted from December 2011 to August 2017. Data analysis was performed from April 25 to August 18, 2021.

**Interventions:**

Patients were randomized 1:1:1 to intravenous haloperidol, ziprasidone, or saline placebo administered twice daily until resolution of delirium, ICU discharge, or 14 days.

**Main Outcomes and Measures:**

Twelve-lead electrocardiograms were used to measure baseline QTc before study drug initiation and telemetry was used to measure QTc before each subsequent dose of study drug. Unadjusted day-to-day changes in QTc were calculated and multivariable proportional odds regression was used to estimate the effects of antipsychotics vs placebo on next-day maximum QTc interval, adjusting for prespecified baseline covariates and potential interactions with sex. Safety end points, including the occurrence of torsade de pointes, were evaluated. All analyses were conducted based on the intention to treat principle.

**Results:**

A total of 566 patients were randomized to haloperidol (n = 192), ziprasidone (n = 190), or placebo (n = 184). Median age was 60.1 (IQR, 51.4-68.7) years; 323 were men (57%). Baseline median QTc intervals across the groups were similar: haloperidol, 458.0 (IQR, 432.0-479.0) ms; ziprasidone, 451.0 (IQR, 424.0-472.0) ms; and placebo, 452.0 (IQR, 432.0-472.0) ms. From day 1 to day 2, median QTc changed minimally: haloperidol, −1.0 (IQR, −28.0 to 15.0) ms; ziprasidone, 0 (IQR, −23.0 to 20.0) ms; and placebo, −3.5 (IQR, −24.8 to 17.0) ms. Compared with placebo, neither haloperidol (odds ratio [OR], 0.95; 95% CI, 0.66-1.37; *P* = .78) nor ziprasidone (OR, 1.09; 95% CI, 0.75-1.57; *P* = .78) was associated with next-day QTc intervals. Effects were not significantly modified by sex (*P* = .41 for interaction). There were 2 occurrences of nonfatal torsade de pointes, both in the haloperidol group. Neither was associated with study drug administration.

**Conclusions and Relevance:**

The findings of this trial suggest that daily QTc interval monitoring during antipsychotic use may have limited value in patients in the ICU with normal baseline QTc and few risk factors for QTc prolongation.

**Trial Registration:**

ClinicalTrials.gov Identifier: NCT01211522

## Key Points

**Question:**

Do antipsychotics increase the QTc interval and the risk of fatal arrhythmias in patients with delirium in the intensive care unit (ICU) with baseline QTc interval less than 550 ms?

**Findings:**

In this secondary analysis of a randomized clinical trial with 566 patients, neither haloperidol nor ziprasidone significantly increased QTc intervals compared with placebo. Additionally, no study drug–related fatal cardiac arrhythmias occurred.

**Meaning:**

The findings of this trial suggest patients with delirium in the ICU with QTc interval less than 550 ms before administration of antipsychotics may experience no clinically significant cardiac arrhythmias attributable to antipsychotic use.

## Teaser

This secondary analysis of a randomized clinical trial evaluates the use of QTc interval monitoring in critically ill patients with delirium who are receiving haloperidol or ziprasidone.

## Introduction

Antipsychotic medications are reported to cause cardiac conduction disturbances and sudden death due to QTc interval prolongation in numerous patient populations.^1,2,3,4,5,6,7,8^ In hospitalized patients, experts recommend monitoring the QTc interval before and at least every 8 to 12 hours after the initiation, an increase in dose, or following an overdose of QTc-prolonging medications.^9^ Additionally, there are well-described differences in baseline QTc interval duration between males and females, with females having, on average, 10-ms longer QTc intervals.^10,11,12,13,14,15,16,17,18,19,20,21^ Given the profound metabolic disturbances and increased medication exposure that frequently accompanies critical illness, it is unclear whether antipsychotic medications pose the same risks of clinically significant QTc interval prolongation among patients in intensive care units (ICUs).

Delirium, an acute neuropsychiatric condition characterized by inattention and cognitive disturbances,^22^ is frequently treated with antipsychotics based on early limited data suggesting that antipsychotic medications may decrease delirium duration and/or severity in the ICU.^23,24,25^ Randomized placebo-controlled trials and meta-analyses, however, have failed to demonstrate that antipsychotics prevent or treat delirium in the ICU.^26,27,28^ Yet, a worldwide survey of ICU practitioners revealed that typical antipsychotics are used to treat 65% of patients with delirium and atypical antipsychotics are used to treat 53% of this population^29^; these patients are likely to have frequent QTc monitoring. Despite this, QTc interval prolongation secondary to antipsychotics has not been robustly studied in critically ill patients.^30^ Using data from a multicenter, randomized, placebo-controlled trial of antipsychotics for treatment of delirium, we investigated the effects of haloperidol and ziprasidone on the QTc interval in critically ill patients with delirium, the impact of sex on these effects, and whether QTc interval prolongation was associated with fatal ventricular arrhythmias.

## Methods

### Study Design and Participants

We analyzed data collected during the Modifying the Impact of ICU-Associated Neurological Dysfunction (MIND-USA) Study,^26^ which was approved by the Vanderbilt University Institutional Review Board. Participants provided informed consent; no financial compensation was provided. This multicenter, double-blind, randomized, placebo-controlled trial enrolled critically ill adults with respiratory failure or shock who developed delirium in a medical or surgical ICU in 1 of 16 participating US medical centers. The study was conducted from December 2011 to August 2017. Full inclusion and exclusion criteria are defined in a previous publication.^26^ This report follows the Consolidated Standards of Reporting Trials (CONSORT) reporting guideline for randomized studies, and the trial protocol and primary statistical analysis plan are available in Supplement 1 and Supplement 2. The analyses of QTc interval presented in this article are original, were preplanned, and have not been previously published.

### Procedures

Trained research personnel assessed all potentially eligible patients for whom informed consent was obtained twice daily for delirium using the validated Confusion Assessment Method for the ICU (CAM-ICU),^31^ 1 of the 2 validated delirium assessment tools recommended by the 2013 American College of Critical Care Medicine clinical practice guidelines for the management of pain, agitation, and delirium in the ICU.^32^ When a patient became delirious (CAM-ICU–positive), a baseline 12-lead electrocardiogram (ECG) was evaluated for QTc prolongation. Patients qualified for randomization if their baseline QTc interval (per the Bazett formula^21^) was less than the US Food and Drug Administration (FDA)– and institutional review board–approved safety threshold (550 ms for most of the trial). The Bazett correction was chosen as it is the most commonly used QT correction.^33,34^

When the trial started, we used a QTc safety threshold of less than 500 ms, but the FDA later approved the inclusion and treatment of patients with QTc less than 550 ms (rather than <500 ms) after an interim analysis found that (1) the average change in QTc observed with the first dose of study drug administration was a 7.4-ms increase (95% CI, 1.9-12.8 ms), (2) only 32 (2%) of 2264 scheduled doses were held due to QTc greater than 500 ms and only 4 of those had a QTc greater than 550 ms, and (3) no episodes of torsade de pointes had occurred in a patient receiving study drug.

When baseline QTc was greater than or equal to 550 ms, randomization was deferred, and reversible causes of QTc prolongation, such as calcium abnormalities, thyroid disease, or other medications, were identified and treated. If QTc measurements remained 550 ms or higher for 5 days, the patient was excluded and never randomized. If the baseline QTc (or a subsequent QTc measured within 5 days of informed consent) was less than 550 ms, the patient was randomized in a 1:1:1 ratio to receive twice daily (every 12 hours) intravenous saline placebo, haloperidol (maximum dose, 20 mg/d), or ziprasidone (maximum dose, 40 mg/d) using a computer-generated, permuted-block randomization scheme, and the first dose of study medication was administered immediately after delirium diagnosis, randomization, and bedside QTc interval screening, if needed. If more than 1 hour elapsed between baseline 12-lead ECG and study drug initiation, a bedside telemetry QTc interval was used to confirm QTc less than 550 ms. The volume and dose of placebo or trial drug was doubled or halved at 12-hour intervals based on the presence or absence of delirium and adverse effects. Placebo or trial drug was discontinued at ICU discharge or after the 14-day intervention period.^26^

Before the FDA-approved protocol modification of the QTc safety threshold, prompted by an interim analysis, we measured postdose QTc within 10 to 30 minutes following the first study medication dose using either manual calculation from bedside telemetry or automatic calculation from bedside monitors, if available. We selected the timeframe of 10 to 30 minutes because the time of onset of intravenous haloperidol is 3 to 20 minutes, and the peak effect of intravenous haloperidol occurs within 15 to 45 minutes post dose. Few data were available on the pharmacokinetics of intravenous ziprasidone. We calculated post-dose QTc interval as both the mean of the 3 highest QTc interval measurements obtained during this period and the peak QTc interval during this same period. If the postdose QTc was more than 50 ms higher than the baseline QTc, we obtained a 12-lead ECG to confirm the QTc interval measurement from telemetry. These postdose QTc measurements were discontinued after the FDA-approved protocol modification.

During the entirety of the trial, we monitored and documented the QTc interval before administration of each dose of study medication. If the QTc was 550 ms or greater on telemetry, we obtained a 12-lead ECG for confirmation. Again, if the 12-lead ECG QTc was 550 ms or greater, we held study drug until a reversible cause for QTc interval prolongation was found and treated and QTc was less than 550 ms on telemetry. Any time that study medication was held for QTc interval prolongation and later restarted, the medication was restarted at half the previous dose (but not less than a minimum of haloperidol, 1.25 mg, or ziprasidone, 2.5 mg). Study medication was permanently discontinued if torsade de pointes or another ventricular tachycardia that resulted in clinical sequelae (eg, hypotension) occurred. Per the trial protocol, which was approved by an independent data safety monitoring board and institutional review boards at each site, the relatedness of arrhythmia events was determined by the site principal investigator based on information provided by the ICU team and electronic health record. All parties involved in decisions regarding associations were blinded to treatment group assignment. Other ECG changes (eg, nonsustained ventricular tachycardia that lasted <30 seconds, did not cause hypotension, and was not treated) were managed by the clinical team.

### Outcomes

Our primary outcome for this analysis was the median change in QTc interval between day 1 and day 2. We selected this outcome because it provided the largest number of patients exposed to study drug. Secondary outcomes included the effect of treatment group on postrandomization day 2 maximum predose QTc interval, correlation between bedside telemetry and ECG measurements, association of sex with QTc interval on day 2 of treatment, and incidence of ventricular arrhythmias across treatment groups. Other exploratory outcomes included daily predose QTc interval across all study days, postdose QTc interval, and association of patient factors with initial postdose QTc interval.

### Statistical Analysis

Data analysis was performed from April 25 to August 18, 2021. We used multivariable proportional odds logistic regression to estimate the effect of haloperidol and ziprasidone on next-day (day 2) QTc, adjusting for the following prespecified covariates, which we selected based on expert opinion and a comprehensive literature review: prerandomization QTc interval, maximum predose QTc interval on day 1, age at enrollment, sex, body mass index at admission, and Sequential Organ Failure Assessment score at randomization. We performed redundancy analysis to ensure that no covariates could completely explain any of the others. To evaluate whether the effects of haloperidol and ziprasidone on day 2 maximum predose QTc interval were modified by sex, we included an interaction term between sex and treatment group in the model. No other interaction testing was conducted. All analyses were conducted based on the intention-to-treat principle.

We also describe the incidence of ventricular arrhythmias due to QTc interval prolongation and the median of each patient’s mean daily predose QTc interval across all study days to visualize the overall trend of predose QTc interval over time. We used Spearman correlation coefficient to analyze the correlation between bedside telemetry QTc interval and 12-lead ECG-measured QTc interval when study drug was held due to QTc interval prolongation.

We summarize continuous variables using median (IQR) and categorical variables using frequency and percentages. We report multivariable regression results using odds ratios (ORs) and 95% CIs. All hypothesis testing was 2-sided, and the findings were significant at *P* < .05. We collected and managed data using REDCap electronic data-capture tools^35^ and performed all analyses using statistical software R, version 4.3.1 (R Foundation for Statistical Computing).

## Results

A total of 566 patients were randomized and included in this study (eFigure 4 in Supplement 3). Baseline demographic characteristics are presented in Table 1. To provide information about the generalizability of the study, race and ethnicity were included. Half of the participants were younger than age 60 years, median age was 60.1 (IQR, 51.4-68.7) years, 323 were men (57%), and 243 were women (43%). Comorbid illness was common, and severity of illness was generally high. Before randomization, QTc intervals were similar in the 3 treatment groups: haloperidol, 458.0 (IQR, 432.0-479.0) ms; ziprasidone, 451.0 (IQR, 424.0-472.0) ms; and placebo, 452.0 (IQR, 432.0-472.0) ms. Participant and study drug dose data when study drug was held due to QTc interval prolongation are included in Table 2. Fewer than 10% of all participants had the study drug held due to QTc prolongation, but when this occurred, it was more common in the antipsychotic groups. The daily median predose QTc interval across all study days is reported in eTable 1 in Supplement 3.

**Table 1.** Patient Characteristics, Study Drug Interruptions, and Arrhythmias by Treatment Group

Characteristic	No. (%)			
	Placebo (n = 184)	Haloperidol (n = 192)	Ziprasidone (n = 190)	Combined (N = 566)
Age at enrollment, median (IQR), y	59.2 (51.7-67.2)	60.5 (51.3-69.0)	60.5 (50.4-69.1)	60.1 (51.4-68.7)
Sex				
Female	77 (42)	84 (44)	82 (43)	243 (43)
Male	107 (58)	108 (56)	108 (57)	323 (57)
Race and ethnicity^a^				
American Indian or Alaska Native	3 (2)	3 (2)	0	6 (1)
Asian	0	1 (1)	3 (2)	4 (1)
Black	26 (14)	23 (12)	27 (14)	76 (13)
White	153 (83)	163 (85)	151 (79)	467 (83)
Multiple races or other race	2 (1)	2 (1)	9 (5)	13 (2)
Charlson Comorbidity Index score, median (IQR)	2.0 (1.0-4.0)	2.0 (0.8-3.0)	2.0 (1.0-4.0)	2.0 (1.0-4.0)
Diagnosis at admission				
Airway protection/upper airway obstruction	53 (29)	46 (24)	44 (23)	143 (25)
Sepsis/septic shock	35 (19)	43 (22)	33 (17)	111 (20)
ALI/ARDS due to infection	31 (17)	32 (17)	28 (15)	91 (16)
ALI/ARDS without infection	8 (4)	12 (6)	7 (4)	27 (5)
Pulmonary, other	16 (9)	16 (8)	14 (7)	46 (8)
Surgery^b^	13 (7)	13 (7)	23 (12)	49 (9)
Chronic obstructive pulmonary disease/asthma	7 (4)	4 (2)	14 (7)	25 (4)
Cirrhosis/hepatic failure	6 (3)	3 (2)	3 (2)	12 (2)
Acute MI/cardiogenic shock	4 (2)	1 (1)	3 (2)	8 (1)
CHF/cardiomyopathy	2 (1)	4 (2)	2 (1)	8 (1)
Arrhythmia	0	1 (1)	1 (1)	2 (0)
Other^c^	9 (5)	17 (9)	18 (10)	44 (8)
APACHE II score at ICU admission, median (IQR)	30.0 (24.0-34.0)	28.5 (23.0-34.0)	28.0 (23.0-34.0)	29.0 (23.0-34.0)
SOFA score at randomization, median (IQR)	10.0 (7.0-13.0)	9.0 (7.0-12.0)	9.0 (7.0-12.0)	9.0 (7.0-12.0)
BMI at admission, median (IQR)^d^	29.6 (23.7-36.3)	29.3 (24.5-36.0)	28.9 (23.9-36.0)	29.1 (24.0-36.0)
Experienced delirium within 14 d, median (IQR)	184 (100)	192 (100)	190 (100)	566 (100)
Delirium duration within 14 d, median (IQR)	4.0 (2.0-8.0)	4.0 (2.0-7.0)	4.0 (2.0-6.0)	4.0 (2.0-7.0)
Experienced coma within 14 d	112 (61)	118 (61)	121 (64)	351 (62)
Delirium coma-free days within 14 d, median (IQR)	7.0 (0-11.2)	8.0 (0-11.0)	8.0 (2.0-11.0)	8.0 (1.0-11.0)
Baseline/prerandomization QTc interval, median (IQR)^e^	452.0 (431.8-472.2)	457.5 (432.0-479.0)	451.0 (424.2-472.0)	453.0 (429.2-475.0)
Study drug ever held for QTc interval prolongation during intervention period^f^^,^^g^	10 (5)	13 (7)	20 (11)	43 (8)
Median of average predose QTc interval for all days during intervention period (among those exposed to study drug), median (IQR)^g^^,^^h^^,^^d^	442.5 (421.4-469.4)	444.0 (420.5-470.0)	445.0 (419.5-465.0)	444.0 (420.5-469.1)
Drug held ever during intervention period for any reason^g^^,^^i^	148 (80)	160 (83)	161 (85)	469 (83)
Death during intervention period	31 (17)	34 (18)	29 (15)	94 (17)
Incidence of ventricular arrhythmia^j^	0	3 (2)	5 (3)	8 (1)
Incidence of torsade de pointes	0	2 (1)	0	2 (0.4)

Abbreviations: ALI, acute lung injury; APACHE, Acute Physiology and Chronic Health Evaluation; ARDS, acute respiratory distress syndrome; BMI, body mass index (calculated as weight in kilograms divided by height in meters squared); CHF, congestive heart failure; ICU, intensive care unit; MI, myocardial infarction; SOFA, Sequential Organ Failure Assessment.

^a^ Race data were obtained from the electronic medical record. Other is anything other than the categories reported herein, with data collapsed because of small sample sizes.

^b^ Surgery included vascular surgery; hepatobiliary/pancreatic surgery; transplants; gastric surgery; colonic surgery; urologic surgery; orthopedic surgery; ear, nose, and throat surgery; and obstetrics/gynecology surgery.

^c^ Other included gastrointestinal bleed, kidney failure, hemorrhagic shock, metabolic/endocrine/electrolyte disorder, other infectious disease, cancer, seizures/status epilepticus, neurologic disease, and other.

^d^ Total number is 564.

^e^ Baseline/prerandomization QTc interval was defined as the most current prerandomization electrocardiogram (ECG) QTc interval on the date of randomization. If the prerandomization ECG QTc on the date of randomization was not available, then the first available bedside telemetry QTc measurement was used. The initial ECG QTc results were used in 2 patients before the first dose on the first intervention day.

^f^ Study drug ever held for QTc interval prolongation was defined as the event that a patient ever experienced study drug hold only due to QTc prolongation during the intervention period.

^g^ Intervention period is the time frame from randomization to study drug discontinuation, which occurred at ICU discharge, death, or 14 days after randomization, whichever occurred first.

^h^ Median of average predose QTc interval for all days is a patient-level variable. The average daily predose QTc was calculated, and the median of the average daily predose QTc for each patient within the intervention period was used.

^i^ Drug held ever during intervention period was defined as the event that a patient ever experienced study drug hold for any reason during the intervention period.

^j^ Ventricular arrhythmia was defined as ventricular tachycardia, ventricular fibrillation, or torsade de pointes recorded within the daily data collection form.

**Table 2.** Study Drug and QTc Intervals

Variable	Placebo	Haloperidol	Ziprasidone	Combined
Patient-level QTc interval information when drug is held due to QTc prolongation				
Total No. of participants	184	192	190	566
Participants with doses held	10 (5)	13 (7)	20 (11)	43 (8)
Predose QTc interval (first hold only) when drug is held for QTc prolongation	522 (508.5-544.8)	532 (511.5-577.8)	535.5 (511.5-572.2)	525 (510.0-569.8)
Next drug status when drug is first held for QTc prolongation				
Drug restarted at next dose	3 (30)	10 (77)	9 (45)	22 (51)
Next dose also held for QTc	4 (40)	0	8 (40)	12 (28)
Next dose held for other reasons	3 (30)	0	3 (15)	6 (14)
Discontinuation/last dose	0	3 (23)	0	3 (7)
Dose-level QTc interval information when drug is held due to QTc prolongation				
Opportunities to receive trial drug	2195	2073	2087	6355
No. of doses held	21 (1)	19 (1)	44 (2)	84 (1)
Predose QTc interval (first hold only) when drug is held for QTc prolongation	517.5 (508.5-528.5)	516.0 (510.0-570.0)	530.0 (520.0-574.8)	524.5 (511.5-570.0)
Next drug status when drug is first held for QTc prolongation				
Drug restarted at next dose	8 (38)	13(68)	14 (32)	35 (42)
Next dose also held for QTc	8 (38)	2 (11)	20 (45)	30 (36)
Next dose held for other reasons	5 (24)	1 (5)	6 (14)	12 (14)
Discontinuation/last dose	0	3 (16)	4 (9)	7 (8)

**Figure 1.**
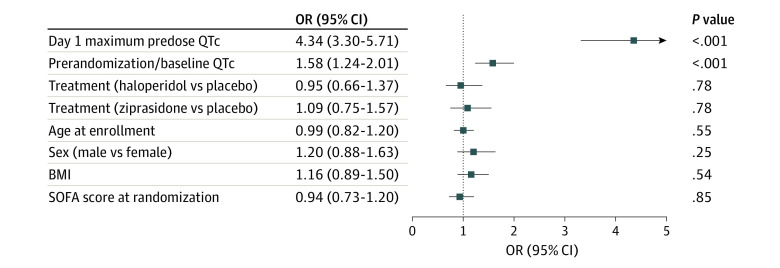
Association of Treatment Group With Day 2 Maximum Predose QTc Interval In analyses adjusting for prerandomization QTc interval, maximum pre-dose QTc interval on day 1, age at enrollment, sex, body mass index (BMI) at admission, and Sequential Organ Failure Assessment (SOFA) score at randomization, there was no association between treatment and day 2 maximum predose QTc, with haloperidol vs saline placebo and ziprasidone vs placebo. The quantitative variable values selected for the ORs represent the 25th and 75th percentile of the distribution of the variable. This common method is useful when examining an association that may be nonlinear and often provides more intuitive results than one that computes an OR from a single-unit change in the exposure variable.

The median QTc interval changes from day 1 to day 2 across the groups were haloperidol, −1.0 (IQR, −28.0 to 15.0) ms; ziprasidone, 0 (IQR, −23.0 to 20.0) ms; and placebo, −3.5 (IQR, −24.8 to 17.0) ms. The median exposure to study drug (antipsychotic or placebo) was 4.0 (IQR, 3.0-7.0) days. After controlling for baseline covariates, neither haloperidol (OR, 0.95; 95% CI, 0.66-1.37; *P* = .78) nor ziprasidone (OR, 1.09; 95% CI, 0.75-1.57; *P* = .78) had a significant population-wide effect on maximum predose QTc interval on study day 2 compared with placebo (Figure 1).

**Figure 2.**
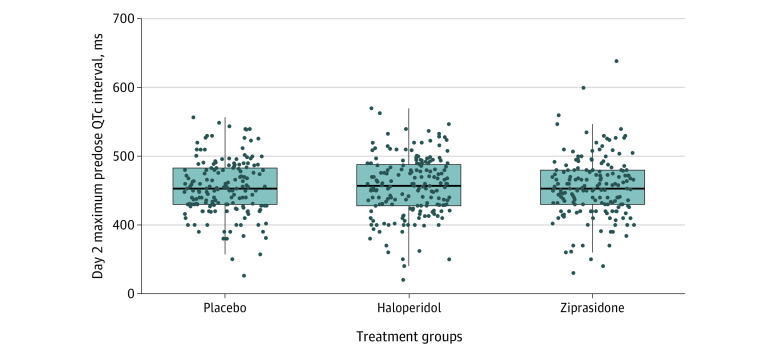
Maximum Predose QTc Interval on Study Day 2 Mean (SD) day 2 maximum predose QTc intervals were 454.6 (40.6) for the placebo group, 455.9 (44.0) for the haloperidol group, and 454.7 (44.8) for the ziprasidone group, and did not differ significantly between groups. Center lines indicate the median; upper and lower bounds of the boxes indicate 25th and 75th percentiles; vertical lines indicate 1.5 times the IQR from each box end; and dots indicate data distribution.

When bedside telemetry and ECG QTc interval values were available and paired (n = 65), the Spearman correlation coefficient was 0.53 (*P* < .001), indicating moderate correlation between the 2 measurements (eFigure 1 in Supplement 3). When bedside telemetry and ECG differed, telemetry-measured QTc was more often longer than 12-lead ECG-measured QTc, especially when the QTc was greater than 550 ms. The distribution of maximum predose QTc interval on study day 2 by treatment group is shown in Figure 2.

There was also no significant difference in initial postdose QTc interval between haloperidol, ziprasidone, or placebo (eFigure 2 in Supplement 3). Among the subset of patients who had both a predose and postdose QTc measured, there was no association between treatment groups and change in QTc (eFigure 3 in Supplement 3). The QTc intervals across treatment groups and sex are described in eTable 2 in Supplement 3. Additionally, the effect of treatment on day 2 maximum QTc interval was not modified by sex (*P* = .41 for interaction).

Torsade de pointes occurred twice in the haloperidol group and never in the other treatment groups. Neither event, however, occurred within 4 days of haloperidol receipt, so these events were deemed unrelated to study drug. Ventricular tachycardia occurred in 5 patients the same day as receiving study drug (2 haloperidol recipients, 3 ziprasidone recipients) and in 1 patient 1 day after study drug receipt (1 haloperidol recipient). Ventricular tachycardia and ventricular fibrillation occurred 1 day after study drug receipt (1 ziprasidone recipient), and ventricular fibrillation occurred 2 days after study drug receipt (1 ziprasidone recipient). However, none of these events resulted in study drug discontinuation because the clinical team did not think these events were associated with study drug administration.

**Figure 3.**
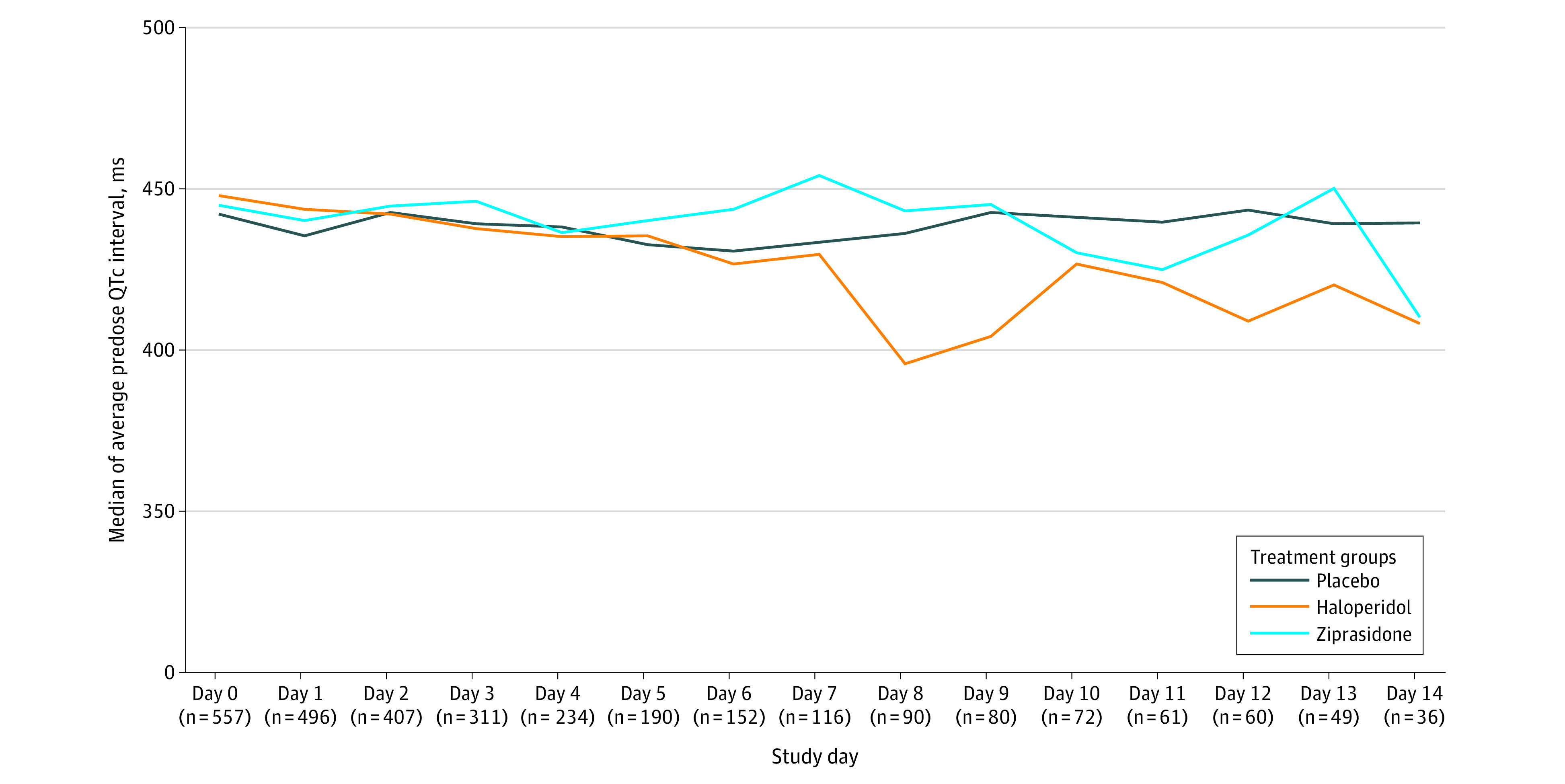
Median Predose QTc Intervals The median of the mean predose QTc interval for all days during the intervention period was 444.0 (IQR, 420.5-470.0) ms in the haloperidol group, 445.0 (IQR, 419.5-465.0) ms in the ziprasidone group, and 442.5 (IQR, 421.4-469.4) ms in the placebo group and showed no discernable trend over time. Study days are considered as time from randomization to treatment group. The sample size provided for each day represents the number of patients who were included in the analysis for that day to estimate the average predose QTc interval.

The median average daily predose QTc intervals did not differ significantly by treatment group over time during the study period (Figure 3). The median of the mean predose QTc interval for all days during the intervention period was 444.0 (IQR, 420.5-470.0) ms in the haloperidol group, 445.0 (IQR, 419.5-465.0) ms in the ziprasidone group, and 442.5 (IQR, 421.4-469.4) ms in the placebo group.

## Discussion

In this large, a priori analysis of a randomized placebo-controlled trial,^26^ we found no evidence that the doses of haloperidol or ziprasidone studied cause clinically relevant QTc interval changes or ventricular arrhythmias in critically ill adults with delirium who had baseline QTc less than 550 ms and low risk for QTc prolongation. These data suggest that the frequency of QTc monitoring during antipsychotic administration to this population should be reexamined.

A previous study suggested that haloperidol, 15 mg/d, prolongs the QTc interval by 7.1 ms, on average.^5^ This prolongation is greater when haloperidol is administered at doses greater than approximately 35 mg/d.^4,36^ With atypical antipsychotics, the incidence of QTc interval prolongation 500 ms or greater varies with the specific medication and is rare in ziprasidone recipients, with an incidence of 0.06%,^5,37,38^ even in the presence of CYP450 3A4 isozyme inhibitors,^5^ common among ICU patients. It is not surprising that the incidence of QTc prolongation in the ziprasidone group in our study was higher than in the previous studies given our study had a larger number of patients who were critically ill and had numerous risk factors for QTc prolongation.

Due to increased risk of drug-induced long-QT syndrome, torsade de pointes, and cardiac arrest in hospital settings, the 2010 American Heart Association and American College of Cardiology statement on prevention of torsade de pointes in hospital settings recommends that “a hospital protocol be established so that a single consistent method is used” to monitor QTc intervals.^39^ We found moderate correlation between telemetry- and ECG-measured QTc intervals, suggesting these 2 modalities may be used interchangeably. Case reports and case series have linked many drugs to torsade de pointes and sudden cardiac death, including antipsychotics, such as haloperidol.^4,39,40^

In an outpatient non-ICU study of Medicaid enrollees in Tennessee, higher rates of sudden cardiac death were noted, with adjusted incidence rate ratios of 1.99 (95% CI, 1.68-2.34) in patients receiving typical antipsychotics and 2.26 (95% CI, 1.88-2.72) in patients receiving atypical antipsychotics, compared with patients not receiving those drugs.^6^ Similar findings were found in another population based study. However, no effect was noted from atypical antipsychotics.^7^ These observational studies differed from our clinical trial in that strict protocols were not in place to prevent administration of antipsychotics to patients with prolonged QTc at baseline. Additionally, many of the deaths in these observational studies may have been noncardiac in etiology such that the risk of sudden cardiac death is overestimated.^41^

Although the risk for torsade de pointes has been shown to be approximately 2-fold higher in women than in men,^10,11,12,13,14,15,16,17,18,19,20^ we found no evidence that sex modified the association between antipsychotics and QTc interval in our study population. However, these results do not rule out an interaction between sex and antipsychotic-indicated QTc prolongation in other populations.

### Strengths and Limitations

This study has several strengths. It was an a priori secondary analysis of a large, multicenter, placebo-controlled, randomized trial that used an FDA-approved QTc interval threshold of 550 ms—higher than current clinical practice in many ICUs. The frequency of and adherence to QTc interval monitoring was rigorous, with telemetry measurements validated using a 12-lead ECG. The QTc interval can have diurnal variations. Twice-daily study drug administration (and therefore predose QTc measurements) at the same times minimized bias due to diurnal limitation. Additionally, our study was conducted in a diverse population of critically ill patients who commonly have electrolyte abnormalities and are receiving many concomitant ICU medications (eg, hypokalemia and hypomagnesemia; amiodarone and voriconazole) that prolong QTc intervals.

Our study also has important limitations. First, we did not record concomitant medications and electrolyte abnormalities that can cause QTc interval prolongation. Study sites, however, were encouraged to identify and correct reversible causes of QTc prolongation. Furthermore, a study by Harrigan et al^5^ did not demonstrate that concomitant medications causing metabolic inhibition prolonged the QTc interval. Second, we used only the Bazett correction from telemetry to provide a heart rate–corrected QTc interval. Other formulas, such as the Fridericia and Framingham, can be used and may be more accurate at faster heart rates. The Bazett correction is the most commonly used correction factor and was therefore applied.^33,34^

Third, each participating institution used its own standard clinical ECG equipment, and the trial required no standardization of ECG algorithms for QT interval measurement. Most ECG systems try to exclude a clear U wave from the T wave when calculating the QT interval, but this separation can be difficult such that the U wave can inflate the QT interval. Fourth, the standard telemetry leads used were 2 and 5 in the supine position, as 2 is more indicative of PR interval and 5 better detects ST-segment elevation and arrhythmias. Placement of leads on the torso is standard of care unless contraindicated, and lead placement can contribute to some QTc variation. However, this should be equal across all groups.

Fifth, we did not correct for atrial fibrillation or bundle-branch block, nor did we record these arrhythmias. It is unknown whether QT prolongation in the presence of bundle-branch block increases the risk of arrhythmias.^42^ In the presence of atrial fibrillation, the QT interval varies from beat to beat depending on the interval between subsequent R waves. There is also no consensus on how to measure the QT interval in the presence of atrial fibrillation.^43^ Sixth, doses of haloperidol and ziprasidone were protocol-mandated, such that we could not analyze the effects of higher doses on QTc or associated outcomes. Higher doses of typical and atypical antipsychotics have been associated with higher risk of sudden cardiac death.^1^ Nevertheless, the maximum daily dose of haloperidol studied was 20 mg/d and of ziprasidone was 40 mg/d; both are similar to doses used in current clinical practice. Seventh, and perhaps most importantly, our eligibility criteria and monitoring protocol prevented the treatment of high-risk patients such that our conclusions likely apply only to patients at low baseline risk of QTc prolongation and related arrhythmias.

## Conclusions

In this a priori secondary analysis of a multicenter, double-blind, randomized, placebo-controlled trial, we found no evidence that antipsychotics cause clinically important QTc interval prolongation or ventricular arrhythmias beyond that of placebo when given to critically ill patients with delirium with baseline QTc less than 550 ms and few risk factors for QTc prolongation. Telemetry- and ECG-measured QTc intervals were moderately correlated, suggesting either approach can be used in most cases. Together, these results suggest that, in low-risk patients, the frequency of QTc monitoring during antipsychotic administration should be reexamined.

## Notes

Supplement 1. Protocol

Supplement 2. Statistical Analysis Plan

Supplement 3. **eTable 1.** Daily Average Predose QTc Interval Across All Study Days **eTable 2.** The Effect of Pharmacologic Medication With QTc Interval Changes by Sex **eFigure 1.** Association Between ECG and Telemetry QTc Interval During Study Drug Held for QTc Prolongation **eFigure 2**. The Distribution of Initial Postdose QTc Interval **eFigure 3.** Association of Patient Factors With Initial Postdose QTc Interval **eFigure 4.** CONSORT Diagram: Screening, Randomization, Follow-Up, and Analysis

Supplement 4.
Data Sharing Statement
