# Supplementary material for: Antipsychotics and the QTc Interval During Delirium in the Intensive Care Unit: A Secondary Analysis of a Randomized Clinical Trial
Source: JAMA Netw Open. 2024 Jan 22;7(1):e2352034. doi: 10.1001/jamanetworkopen.2023.52034 (PMC10804270; doi:10.1001/jamanetworkopen.2023.52034)
Supplement: Supplement 1. — Protocol [file jamanetwopen-e2352034-s001.pdf]

The MIND-USA Study

Modifying the Impact of ICU-Associated Neurological Dysfunction

FDA-IND #104322

E. Wesley Ely, MD, MPH
Timothy D. Girard, MD, MSCI

Department of Medicine
Division of Allergy, Pulmonary, and Critical Care Medicine
Center for Health Services Research
Vanderbilt University School of Medicine

Geriatric Research, Education and Clinical Center (GRECC) Service
Department of Veterans Affairs Medical Center
Tennessee Valley Healthcare System

Contact Details

Primary Investigator	E. Wesley Ely, MD, MPH	
	Phone	(615) 936-2795
	Fax	(615) 936-1269
	E-mail	wes.ely@vanderbilt.edu
Co-Investigator	Timothy D. Girard, MD, MSCI	
	Phone	(615) 936-5069
	Fax	(615) 936-1269
	E-mail	timothy.girard@vanderbilt.edu

Table of Contents:

1.0 Introduction and Specific Aims
2.0 Background
3.0 Preliminary Studies
4.0 Study Objectives and Endpoints
5.0 Inclusion/Exclusion Criteria
6.0 Enrollment/Randomization
7.0 Study Procedures
8.0 Risks of Investigational Agents/Devices (side effects)
9.0 Reporting of Adverse Events or Unanticipated Problems involving Risk to Participants or Others
10.0 Study Withdrawal/Discontinuation
11.0 Statistical Considerations
12.0 Privacy/Confidentiality Issues
13.0 Follow-up and Record Retention
14.0 References

1.0 Introduction

Intensive care will soon represent 2% of the United States' gross domestic product,[1] and nearly two-thirds of intensive care unit (ICU) days are accounted for by people over 65 years old.[2] The population of aging Americans filling our ICUs is especially vulnerable to the development and persistence of delirium.[3-6] We and others have shown ICU delirium to independently predict a 3-fold higher mortality within a year,[7-9] longer hospital stays,[10,11] much higher costs of care,[12] and long-term cognitive impairment.[13,14] Delirium, a form of acute brain dysfunction, develops in 7 out of 10 mechanically ventilated patients in general medical and surgical ICUs.[7,15-18] Delirium's high prevalence and independent relationship with poor outcomes in critically ill patients have led to numerous recommendations that delirium monitoring be part of the standard daily assessment in all ICU patients.[19-21] Delirium is usually multifactorial,[22,23] and treatment of underlying disturbances represents first-line management. Even after addressing modifiable risk factors for ICU patients, however, delirium often persists from a few days to months. Antipsychotics are recommended as the medication class of choice for delirium by all major clinical guidelines,[19-21,24] yet very little evidence exists to support this internationally recognized and adopted treatment.[25-28] Because the vast population of older ICU patients are at very high risk for delirium, millions of older patients around the world receive significant amounts of intravenous antipsychotic medications for delirium despite the fact that no placebo-controlled trial has definitively demonstrated efficacy or safety of these medications for ICU delirium. Tens of thousands of ICU patients receive antipsychotics for delirium every day with limited evidence to establish best practice for effective pharmacologic management of this debilitating syndrome. Haloperidol, a "typical" antipsychotic, is the most commonly chosen, used by 86% of ICU practitioners.[29,30] Atypical antipsychotics, of which there are currently eight available in the U.S., are the next most commonly utilized drug for delirium in ICUs; 38% of practitioners use atypicals.[29,30] Despite their popularity, the use of antipsychotics for delirium remains controversial. Concerns about the safety of these drugs are fueled by well-known toxicities, including lethal cardiac arrhythmias and extrapyramidal symptoms, as well as reports of increased death rates associated with antipsychotic use in ambulatory geriatric populations.[31-37] **The MIND-USA study is a multicenter, double-blind, randomized, placebo-controlled trial investigating the effects of intravenously (IV) administered typical and atypical antipsychotics (haloperidol and ziprasidone) on delirium in critically ill patients, many of whom are older than 70 years**. This study will have adequate (> 80%) power to detect a therapeutic difference between haloperidol and an atypical antipsychotic (ziprasidone) versus placebo in a broad adult ICU population and within important subgroups such as severely ill older patients with significant comorbidities. In the burgeoning field of aging brain research—which involves the disciplines of medicine, surgery, geriatrics, psychiatry, critical care, neuroscience, nursing, and clinical pharmacology—this timely interdisciplinary investigation of a common, expensive, deadly condition without known effective therapy will both direct clinical practice and shape future work.

Hypothesis: The administration of typical (haloperidol) and atypical (ziprasidone) antipsychotics to critically ill patients with delirium will improve short- and long-term clinical outcomes.

Specific Aims

Aim 1: To determine whether haloperidol or ziprasidone administered to delirious medical/surgical ICU patients will increase days alive without delirium (measured as delirium/coma-free days [DCFDs]) over a 14-day study period compared with placebo and compared with one another.

Aim 2: To determine whether haloperidol or ziprasidone will improve 30-day, 90-day, and 1-year survival compared with placebo and compared with one another.

Aim 3: To determine whether haloperidol or ziprasidone will reduce ICU length of stay (LOS) (i.e., time to ICU discharge) compared with placebo and compared with one another.

Aim 4: To determine whether haloperidol or ziprasidone will improve long-term neuropsychological outcomes, functional independence, quality of life, and posttraumatic stress disorder symptoms at 3-month and 1-year follow-up compared with placebo and compared with one another.

To complete these Aims, we will consent adult medical and surgical ICU patients (a) on mechanical ventilation or non-invasive positive pressure ventilation, and/or (b) in shock requiring vasopressors. Once delirium occurs, we will randomize a total of 561 delirious patients (187 to the haloperidol group, 187 to the ziprasidone group, and 187 to the placebo group) and follow them for 1 year. In the absence of an indication requiring hold of study drug, patients will receive study drug until delirium has resolved for two days or ICU discharge or 14 days after the day of randomization, whichever occurs first. While tracking **primary** (delirium duration, Aim 1) and **secondary outcomes** (mortality, ICU LOS, and long-term neuropsychological function; Aims 2-4), we will closely monitor safety parameters, including arrhythmias, extrapyramidal symptoms (EPS), neuroleptic malignant syndrome (NMS), and posttraumatic stress disorder (PTSD). **The study sample size was explicitly chosen to ensure adequate power (≥ 80%) to detect the effect of antipsychotics in four important subgroups**: age ≥ 70 years, high severity of illness (APACHE II ≥ 25), presence of severe sepsis at enrollment, and medical vs. surgical ICU patients. We will also conduct hypothesis-generating subgroup analyses of the effect of antipsychotics on delirium in patients with pre-existing cognitive impairment.

2.0 Background

> **"For many aging people in good physical condition, who succumb to an acute illness, cognitive decline [e.g., delirium followed by an acquired post-ICU long-term cognitive impairment] is the main threat to their ability to recover and enjoy their favorite activities; for those whose physical activities were already limited, cognitive decline is a major additional threat to quality of life."**
>
> -National Research Council[38]
>
> **"In patient populations for whom the evidence of the efficacy of antipsychotic medications is limited…prudence would suggest that the use of these drugs should be reduced sharply."**
>
> -January 2009 NEJM[39] *(yet data show sharp increases in ICU use recently)*[29,40]

2.1 The Magnitude of the Problem of Delirium in Critically Ill Patients. Delirium is a syndrome of acute organ dysfunction occurring in 50% to 80% of ICU patients.[11,15,16,18,41-45] The DSM IV-TR[46] and the CAM-ICU define delirium as an acute form of brain dysfunction characterized by a change in baseline mental status and inattention, plus disorganized thinking or an altered level of consciousness as noted in **Figure 1**,[47] which distinguishes delirium from coma, and highlights the cardinal symptoms of delirium. A **dashed line** encircles optional symptoms of delirium (i.e., those sometimes present but not mandatory for a diagnosis of delirium). Whereas "respiratory failure" is the most common reason for ICU admission across ages, delirium (i.e., brain "failure") is the classic geriatric syndrome serving as the strongest determinant of hospitalized patients' length of stay and disposition.[10] Delirium in ICU patients carries enormous financial and societal burdens due to its association with increased morbidity, mortality,[7] and prolonged hospital stays,[10] as well as its relationship to long-term neuropsychological deficits among ICU survivors.[48-50] In fact, the occurrence of ICU delirium predicts a 3- to 13-fold rise in the likelihood of death by 1 year after adjusting for severity of illness, coma, sedatives, and other relevant covariates.[7,8] **In addition, each additional day of delirium independently increases the risk of death by 10% (p=0.03),[7,9] strongly supporting Aims 1 and 2 of the MIND-USA study.** It is estimated that ICU delirium is associated with costs ranging from $4 to $16 billion annually in the U.S. alone,[12] not including added cost of lost workdays, caregiver burden, or cognitive rehabilitation for the 66% of patients with ongoing neuropsychological deficits at 6 months and the 50% with ongoing deficits at 24 months following their ICU stay.[13,48,51,52] Due to the increased utilization of intensive care and our aging population, who have multiple medical problems, pre-existing cognitive impairment, and increased risk of functional decline,[53] the toll of ICU delirium is likely to grow exponentially in upcoming decades.[54] This toll is not merely measured in lives lost. Links are increasingly well established between delirium and long-term morbidities, including impaired quality of life, frailty, depression, anxiety, PTSD, and persistent cognitive dysfunction akin to dementia (supporting Aim 4 of MIND-USA).[55] An intervention to reduce delirium may allow more patients not only to survive but also to live more meaningfully.

Figure 1. Defining Delirium and Coma

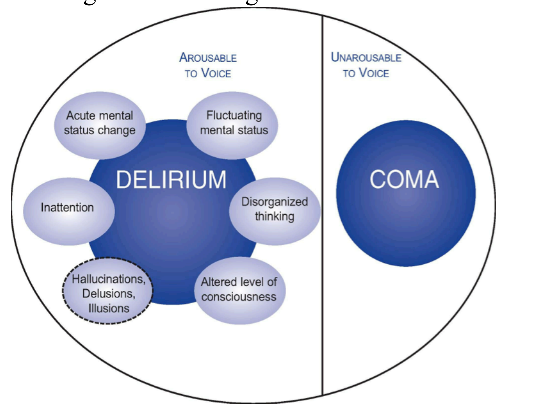

As a result of the above-mentioned facts, the Society of Critical Care Medicine (SCCM) and numerous others internationally have recommended routine monitoring for delirium in all ICU patients.[19-21] **Monitoring increases detection and has resulted in earlier and widespread increases in treatment with typical and atypical antipsychotics[29,30,56-58] despite cautionary studies.[37,39,59,60]** The majority of health care professionals in a national survey conducted in 2002[40] believed that delirium was a vital yet inadequately addressed problem in critically ill patients and that there were few data directing appropriate therapy for this condition. Unfortunately, only a few randomized trials of any pharmacological interventions in ICU patients have improved our knowledge base,[18,45,61,62] though we are poised to correct this deficit in our understanding of the efficacy and safety of antipsychotics for delirium.

Other approaches to managing delirium, such as multicomponent prevention protocols, have been shown in non-ICU populations to improve the incidence and duration of delirium but not mortality.[63-68] Unfortunately, none of these studies have included ICU patients and the marked clinical differences of the ICU population (higher number of risk factors for delirium, difficulty with communication due to intubation, and omnipresent use of sedatives) limit the utilization of these protocols in the ever-expanding population of ICU patients.

2.2 Aligning Proposed Mechanisms of Delirium with Recommended Therapy. Delirium is thought to result from imbalances in neurotransmitters within the brain.[69-73] These imbalances form the *fundamental scientific rationale and biological plausibility* for our investigation. It is not the purpose of this research, nor would it be possible within such a large trial, to measure or document correction of neurotransmitter imbalances. This section provides a basic introduction to the concepts underlying this study proposal. It is hypothesized that delirium treatment may be achieved by partial correction of neurotransmitter imbalances via haloperidol or ziprasidone, with the secondary aim of minimizing adverse effects. This last point is important because drugs that help correct a neurotransmitter at one site might improve delirium yet cause an adverse event via the same neurotransmitter at another site or via downstream effects on alternative neurotransmitters.[74,75] **In general, neurotransmitter imbalances that have received the most attention in literature are dopamine excess, acetylcholine deficiency, and interactions between the serotonergic and dopaminergic systems.[73] Importantly, there appears to be a link between dopaminergic and acetylcholine hypotheses for delirium that *support a biological rationale for the MIND-USA study*.[76-82]** These references and others indicate pharmacological and neuroanatomical evidence that acetylcholine and dopamine appear to be inversely related in delirium pathogenesis. Anatomically, dopaminergic and cholinergic pathways overlap in the brain: D_2 receptors for dopamine, which inhibit acetylcholine synthesis, coincide with cholinergic fibers in layer V of the prefrontal cortex. Common events in the ICU, such as hypoxia or hypotension (to be tracked objectively in MIND-USA), can result in surges of dopamine that decrease acetylcholine and precipitate or exacerbate delirium. Neurotransmitter derangements may also be secondary to causal factors that ICU patients frequently possess including severe sepsis, multiple organ dysfunction syndrome, hypoxemia, metabolic disturbances, sleep deprivation, exogenous toxic agents, withdrawal from substances of abuse, and exposure to psychoactive medications, such as narcotics and benzodiazepines, which are indicated but often given in much higher doses and for longer durations than required. Indeed, ICU patients often have greater than 10 risk factors for delirium.[10,16,43,83-86] Critically ill patients experiencing these problems are hypothesized to develop acute brain dysfunction/delirium due to the deranged neurotransmitter levels noted above, a concept that forms *the basis of current worldwide (though controversial) practice of treating delirium with typical and atypical antipsychotics, the efficacy and safety of which will be tested in the MIND-USA trial.*

2.3 State of the Evidence Regarding Haloperidol and Atypical Antipsychotics in Treatment of ICU Delirium. Haloperidol (used by 75-80% of intensivists) and more recently atypical antipsychotics (used by 35-40%) have emerged as the standard pharmacological treatments for delirium in the ICU.[29] In the Society of Critical Care Medicine (SCCM)[19] and other societal guidelines[87,88] for the management of pain, anxiety, and delirium in the ICU, haloperidol is recommended as the drug of choice for delirium, though this is based on sparse outcomes data from nonrandomized case series and anecdotal reports. *The dearth of data in this*

area was emphasized in recent systematic reviews[25,27,28,89] that concluded as follows: (1) "To date, there are no published double-blind, randomized, placebo-controlled trials to establish efficacy or safety of any antipsychotic medication in the management of delirium. There is limited evidence from uncontrolled studies that supports the use of low-dose, short-term treatment of delirium with some antipsychotics. Further study with well-designed clinical trials is required in this area,"[89] (2) "Better designed and larger studies evaluating the addition of antipsychotic agents to nonpharmacologic treatments are needed to measure the true effect of pharmacologic treatment,"[25] (3) "Small studies of limited scope require further corroborating evidence before translation into specific recommendations for delirium treatment."[27] **Consistent with these conclusions, in their newly drafted guideline on the diagnosis, prevention and management of delirium, the National Institute for Health and Clinical Excellence (NICE) has explicitly called for a large, randomized placebo-controlled trial of haloperidol and atypical antipsychotics.[21]**

Haloperidol has been touted for over 15 years as a preferred delirium treatment.[90-94] In a 2002 survey[40] of 638 professionals who reported treating delirium with haloperidol or atypical antipsychotics, the top reasons they used these agents were few side effects and satisfactory anecdotal efficacy. Still, over half of the respondents reported witnessing adverse reactions associated with haloperidol. A double-blind, randomized investigation of delirium in AIDS patients found that haloperidol was equal to chlorpromazine regarding delirium outcomes, and both were superior to lorazepam, which exacerbated delirium[95] (a characteristic of benzodiazepines now shown consistently in ICU patients).[6,16,18,45,86] More recently, a large, placebo-controlled prophylaxis study in elderly hip fracture patients (n=430) showed reductions in the severity (p<0.001) and duration (p<0.001) of delirium, but without lowering incidence (~16% in both groups).[96] A retrospective cohort study concluded that haloperidol in ventilated patients was associated with lower mortality.[97] Atypical antipsychotics—clozapine, ziprasidone, olanzapine, risperidone, aripiprazole, and quetiapine—are used at varying rates anecdotally throughout the world, though very sparse supportive data exist in non-ICU[98-103] and ICU patient populations.[62,104] Risperidone (in non-ICU patients)[99,103] and olanzapine (in ICU patients)[62] have been randomized against haloperidol, and in both studies there was a temporalreduction in delirium without differences between groups. The olanzapine vs. haloperidol study was an unblinded, non-placebo-controlled trial of 73 low severity and largely non-ventilated ICU patients that concluded olanzapine was a safe alternative to haloperidol.[62] Most recently, in a prospective trial[105] of 36 patients randomized to either quetiapine or placebo (n=18 per group) after having received ~3 to 5 mg of haloperidol, it was found that the haloperidol plus quetiapine treated patients experienced faster resolution of their delirium by 3.5 days as compared with the group receiving haloperidol plus placebo. In addition, the quetiapine-treated patients were over 30% more likely to be discharged home or to rehabilitation than the placebo-treated patients. The lack of a true placebo group in any of these trials precludes any firm conclusions about the role of antipsychotics in ICU delirium.

Regarding survival, however, 5 retrospective studies and 1 prospective placebo-controlled study in non-ICU, non-delirium patients (i.e., predominantly demented elderly cohorts)[31-37] have reported an association between long-term use (e.g., 2 to 3 months or longer) of both typical and atypical antipsychotics and increased mortality rates (prompting an FDA warning). **Whereas both the duration of use and the patient population will be much different in the MIND-USA trial, these reports of increased mortality with antipsychotic use coupled with our data showing rising rates of use of antipsychotics in ICU patients for**

delirium mandate that the medical community confirm the safety of short-term use in ICU patients.[29,40] ICU patients' vulnerability to adverse effects may be favorably balanced by their short term (days rather than months) exposure meeting the acute need for delirium treatment in a very well-monitored setting, especially in light of alternatives otherwise used such as benzodiazepines.

2.4 Mechanism and Safety Profile of Antipsychotics. The mechanism of action of haloperidol is believed primarily to be antagonism at dopamine (D_2)[106-109] and $5HT_{2A}$[74] receptors. In addition, its anti-inflammatory effects[97,110] may help mitigate organ dysfunction in critical illness. Dopamine blockade in the cerebral cortex could improve cognition and reduce delirium. The potent affinity of haloperidol for D_2 receptors extends beyond the cortex. Haloperidol also causes concomitant nigrostriatal pathway D_2 blockade, disinhibition of acetylcholine (i.e., acetylcholine excess), and a risk for developing EPS acutely and dystonia when used chronically.[109,111] In the 2002 survey,[40] the most frequently mentioned adverse effects of haloperidol were oversedation, EPS, NMS, QT interval prolongation and torsades de pointes, respiratory complications, dystonia, and worsening delirium. Initially, potential adverse effects of haloperidol appeared to exceed those of atypical antipsychotics,[112,113] but recent data on adverse events such as EPS, NMS, tardive dyskinesia, glucose and cholesterol abnormalities, cardiac dysrhythmias, and venous thromboembolism appear largely to counter-balance one another between typical and atypical agents[37,114-122] and have called into question the safety profile of both typical and atypical antipsychotics in older patients (especially the demented elderly).[32-36,36,122-124]

Atypical antipsychotic agents, compared with haloperidol, have a wider variety of reported affinities for CNS receptors, including dopamine, serotonin, adrenergic, and muscarinic receptors.[107,125] Ziprasidoneis an antagonist at D_2, H_1, α_1, 5-HT_{2A}, 5-HT_{2C}, and 5-HT_{1D} receptors and an agonist at 5-HT_{1A} receptors.[125,126] Like other successful antipsychotic medications, it has high affinity for the D_2 receptor, though this is not the majority of its receptor activity (as seen with haloperidol). It is distinguished from other atypicals by blocking reuptake of norepinephrine and 5-HT.[126] As stated above, atypical antipsychotics typically have a lower incidence of EPS versus haloperidol.[112,127] A high $5HT_{2A}/D_2$ receptor affinity ratio, which is a particularly strong characteristic of ziprasidone, has been correlated with lower propensity for EPS and may be advantageous in treating negative symptoms[126] so prevalent in hypoactive delirium. The α_1 receptor affinity of ziprasidone is reduced in comparison to other atypical antipsychotic medications, which may translate into less orthostatic hypotension. It increases central nervous system acetylcholine and through agonism at the $5HT_{1A}$ receptor (as seen with the antidepressant and anxiolytic buspirone) can increase dopamine in the cortex, both of which theoretically would be helpful for delirium.

The strikingly divergent receptor affinities of these two classes of antipsychotics adds to the clinical conundrum surrounding the fact that they are both used anecdotally in widely varying rates across the country for treatment of delirium. Thus, in addition to being the first ever adequately powered trial to compare antipsychotics vs. placebo for ICU delirium, MIND-USA will also be a major step toward making evidence-based decisions about which class of antipsychotics is most effective for critically ill delirious patients. Lastly, the trial may begin to shed light on the complex and poorly understood neurophysiologic derangements underlying delirium in critically ill patients.[128]

2.5 QT Prolongation and Haloperidol and Ziprasidone. Antipsychotic medications as a class pose a risk of cardiac conduction disturbance and sudden death.[6,37,59,59,129-134] The risk of QT prolongation and torsades de pointes appears to be cross-class.[135] In fact, haloperidol had the lowest rate ratio for cardiac death among typical and atypical agents in a recent *NEJM* report.[37] It has been noted that haloperidol's average QTc prolongation at 15 mg/d is 7.1 milliseconds. This modest increase in QTc has rarely been associated with significant arrhythmia risk as long as baseline and "on-treatment" QTc are < 500 ms, which is easy to detect on continuous ECG monitoring as will be used in all ICU patients for MIND-USA.[132,136] The risk is dose-related[130] and greater for haloperidol when > 35mg per day, nearly double the maximum dose to be used in this trial.[131] The incidence of QT prolongation varies with the specific type of atypical antipsychotic. It is considered rare with ziprasidone[137] at 0.06% QTc ≥ 500 msec (data from 2004 Up-To-Date report on ziprasidone), and one study found that ziprasidone and haloperidol have equivalent effects on the QTc interval.[138] The clinical significance of this associated QT prolongation is not known, yet the occurrence of arrhythmias (including torsades de pointes) has been reported as rare. In a study using a total of 50mg of IM ziprasidone and 17.5mg of IM haloperidol over 4 hours, the average increase in QTc from baseline in the ziprasidone group was 12.8 msec at Cmax and in the haloperidol group it was 14.7 msec at Cmax. In this study no patient had a QTc interval exceeding 500 msec (data from package insert for ziprasidone).

2.6 Route of Administration, Pharmacokinetics, and Drug Interactions of Haloperidol and Ziprasidone. We have an FDA IND for the MIND-USA investigation (IND #104322) that includes three treatment groups (haloperidol, ziprasidone, and placebo). Both study drugs, haloperidol and ziprasidone, may be delivered reliably and with rapid onset via the PO and IM routes, though the IV route is preferable in an ICU setting, where patients frequently do not have enteral access and coagulopathy precludes IM injections. **Whereas haloperidol has routinely been administered IV for decades (despite the absence of FDA approval for this route), the IV use of atypical antipsychotics is less common but is reported in case reports[139-141] and in one phase I study of healthy volunteers (N=12 received IV ziprasidone with no ill effects).[142]** For example, a 47-yr-old mechanically ventilated ICU patient experiencing profound delirium despite multiple doses of haloperidol and sedatives received a 20mg IV bolus of ziprasidone with "dramatic" improvement in restlessness and delirium and no extrapyramidal or cardiac side effects.[139] In the only report of extrapyramidal side effects due to IV ziprasidone, symptoms completely resolved after treatment with single doses of diphenhydramine and benztropine.[143] Both haloperidol and ziprasidone are greater than 90% protein bound. The mean half-life of haloperidol is 21 hours with N-dealkylation being the predominant pathway of metabolism; all of the metabolites are inactive with the possible exception of an active hydroxyl metabolite. The mean half-life of ziprasidone is 7 to 10 hours, with CYP 3A4 being the predominant isoenzyme involved in metabolism. The IM formulation of ziprasidone contains a solubilizing agent called β-cyclodextrin (in this case, specifically SBE-β-CD) that has been safely used in other IV medications (e.g., itraconazole and voriconazole) at much higher concentrations.[144,145] In fact, in this investigation our dosing range will have around 3% of what is given routinely with voriconazole, and even in study patients with renal impairment, they will only receive around 15-18% of what is given to humans currently with FDA-approved use of IV voriconazole. Haloperidol is extensively metabolized by the liver's CYP2D6, CYP3A4, and CYP1A1 enzymes,[74,146-150] leaving 1% of administered dose excreted unchanged in urine. Haloperidol has metabolic interactions with ~20 drugs,[148,150] but those of considerable hazard are infrequently

used in ICU patients. Some important candidates to consider include carbamazepine and phenytoin, which can cause increased haloperidol metabolism and lower its levels. Others with the opposite effect, thereby possibly causing increased concentrations of haloperidol, include buspirone, fluoxetine, and itraconazole. When given concomitantly with lithium, CNS toxicity can result. Rather than exclude patients on these agents and thereby lose the ability to advance our understanding through the context of a controlled trial, methods have been designed to collect haloperidol plasma concentrations and to monitor safety parameters, including continuous cardiac monitoring. Ziprasidone is unlikely to cause significant interactions with drugs metabolized by the cytochrome P450 system. There are no clinically significant interactions with lorazepam or other benzodiazepines, narcotics, H2 blockers, propranolol, or lithium.

2.7 Placebo "Treatment" of Delirium as the Comparator for the MIND-USA Trial. Justification for a placebo treatment group as the primary comparator for this trial rests on four distinct and firm issues. **First**, though many delirious ICU patients receive antipsychotics, the *majority* of delirious patients actually receive no specific treatment, largely due to the fact that delirium, especially hypoactive delirium,[151-153] remains unrecognized over 66% of the time in clinical practice.[152-160] Of note, < 5% of ICU delirium is pure hyperactive, with the vast majority being hypoactive or mixed type.[153] While delirium monitoring is now available, data from the 2002 survey indicated that fewer than 5% of practicing ICU healthcare professionals used a specific delirium monitoring instrument.[40] Even though this rate is now up to 33% and rising, most delirium in the ICU is not recognized or treated, which serves as the first major rationale for this placebo-controlled investigation. **Second**, there are no FDA-approved medications for treatment of delirium. Outside of the pilot studies, there are no placebo-controlled trials of any medications to treat delirium in the highest risk patients, namely older medical and surgical ICU patients. **Third**, it is possible that typical and atypical antipsychotics could induce harmful effects unrelated to delirium or even via adverse effects on delirium itself.[161] As will be presented below, haloperidol could actually precipitate or worsen delirium in ICU patients, and the placebo group of our pilot MIND study experienced no apparent harm. **Fourth**, and perhaps most importantly, the definitive study must include a placebo comparison to account for spontaneous improvements in delirium. *Such equipoise regarding efficacy and safety of any therapy, especially one as routinely yet erratically prescribed as antipsychotics in the care of fragile, delirious ICU patients, presents a solid rationale for the conduct of a landmark, randomized, placebo-controlled clinical investigation that will be accomplished via this MIND-USA trial.*

2.8 Conclusions on Background Evidence in Support of MIND-USA Trial. Taken together, this background section highlights two main points: (1) There has been an accelerated path of medical research in the area of delirium, especially among the fastest growing segment of the ICU population—older patients with acute potentially life-threatening illnesses—which happens to have the highest rates of delirium. (2) A dilemma in medicine is clinicians who monitor critically ill patients for brain organ dysfunction are posing the same question that geriatricians, psychiatrists, and neurologists have been for 20 years: ***"If I prescribe the recommended antipsychotics (haloperidol or an atypical antipsychotic) for my delirious patients, will it help them or hurt them?"*** In addition, many physicians not monitoring objectively for delirium are waiting for level I evidence from a placebo-controlled multi-center trial to inform them whether or not haloperidol should be prescribed, and if so, in whom and for

what reasons. **The MIND-USA Trial has been designed to address this dilemma.** Whether positive or negative, this trial will shape practice due to the driving unmet need for hard data to direct delirium management at a time when practice remains random from hospital to hospital, doctor to doctor, and patient to patient. In 2010, equipoise remains regarding efficacy and safety of routinely prescribed typical and atypical antipsychotics in the care of fragile, delirious ICU patients.[30]

3.0 Preliminary Studies

3.1 Haloperidol is a Possible Precipitant of Delirium. Dr. Margaret Pisani, a MIND-USA co-investigator and the site PI at Yale, conducted a prospective observational cohort study enrolling 309 patients aged 60 and older from 2002 to 2004.[6] This study was designed to examine risk factors for ICU delirium, specifically psychoactive drug use. Detailed medication, physiologic, and delirium data (using the CAM-ICU) were collected daily. Median duration of delirium was 3 days. In a multivariable model, receipt of haloperidol (analyzed as a time-varying covariate) was associated with increased delirium duration [OR=1.35 (1.21-1.50), p<0.001], as were benzodiazepine and opioid use [1.64 (1.27-2.10), p<0.001], dementia [1.19 (1.07-1.33), p=.002], and APACHE II score [1.01 (1.00-1.02), p=0.02]. Risk factors for persistent delirium, defined as a continuous episode of ICU delirium that continued to the ward, were examined. Of 173/309 (56%) patients with ICU delirium, 100 (58%) had persistent delirium. Multivariable regression using time-varying covariates showed haloperidol to be a predictor of persistent delirium [2.88 (1.38-6.02), p=0.005], as was age > 75 (2.52, p=0.01) and high dose opioids (2.9, p=0.02). *While not conclusive, these data establish equipoise to consider that haloperidol might cause harm.*

3.2 Pilot Investigation of IV Haloperidol in Titratable Dose Format. Dr. Ivor Douglas, a MIND-USA co-investigator and the site PI at University of Colorado, conducted a small pilot study to explore the safety profile of IV haloperidol in addition to standard sedation in delirious mechanically ventilated patients.[162] Once patients met CAM-ICU delirium criteria, they were randomized to either placebo (n=9) or stepped-dose IV haloperidol (n=8) in addition to usual care (including low dose haloperidol) for ICU sedation. Patients in the active treatment group received haloperidol q6 hours with 2 to 4 mg stepped increase or decrease in dose based on presence or absence of delirium. The haloperidol treatment group received 13.1±10.6 mg/d and through open-label use the control group received 2.3 ±1.9 mg/d (p<0.001). Median duration of intubation was 3.5 d (control) vs. 3 d (intervention, p=0.24), and median durations for ICU stay were 10 d (control) and 8.3 d (intervention, p=0.50). Median Folstein Mini-Mental State Examination (MMSE) scores at ICU discharge were 19.5 (control) and 30 (intervention, p=0.08). Two patients in the control group died. Adverse events included one self-extubation in each group, one patient in each group developed QTc prolongation to ≥ 500 msec, one intervention patient experienced paradoxical increase in delirium, and one in the control group had a possible seizure. **These preliminary data suggest that titratable dose IV haloperidol was well-tolerated and a suitable approach to adopt for a large-scale, definitive, randomized controlled trial.** *The trend towards better MMSE scores in patients who received an average of 13mg/d of haloperidol is consistent with the cognitive change literature recently subjected to meta-analysis,*[163] *which reported that overall cognitive performance improves while on haloperidol, high dose (≥ 10mg/d) yielded similar effect size compared to doses < 10 mg/d,*

although doses ≥ 25mg/d had deleterious effects. The MIND-USA investigators want to give the least amount of haloperidol required to balance efficacy with the need in ICU patients to provide some sedation so that breakthrough and potentially deliriogenic sedatives are minimized.

3.3 The MIND Pilot Feasibility Trial.[44] The MIND trial was designed to test the feasibility of a protocol to address the hypothesis that antipsychotic medications will improve the duration of delirium in a safe manner. In conducting the MIND feasibility study, we included both typical and atypical antipsychotics. **The MIND trial was double-blind and randomized placebo vs. haloperidol vs. ziprasidone in 103 medical and surgical ventilated ICU patients at 6 centers.[44]** Study drug was administered PO or IM because the FDA would not allow IV administration of atypical antipsychotics at that time since they are not available in this formulation, though they indicated they would allow IV haloperidol. The study was exempted by the FDA for an IND. An atypical antipsychotic was included because this class of drugs might have a favorable safety profile as compared with haloperidol and because of varied affinities of atypical antipsychotics at dopamine, serotonin, adrenergic, and muscarinic receptors.[75,107] Ziprasidone was chosen because it is an antagonist at D_2, H_1, α_1, 5-HT_{2A}, 5-HT_{2C}, and 5-HT_{1D} and a 5-HT_{1A} agonist.[126] Like other successful antipsychotic medications, it has high affinity for the D_2 receptor, though this is not the majority of its receptor activity. **Haloperidol was given in doses of 2.5 to 5 mg ranging from once per day to every 6 hours (i.e., haloperidol dose ranged from 2.5 to 20 mg per day).** *This moderate dose range, while higher than used by most geriatricians and psychiatrists yet lower than the range currently used in most ICUs, was (a) supported by psychiatric "cognitive change" literature,[163] and (b) important in the ICU because haloperidol provides patient sedation. Because sedation is usually perceived as more urgent than resolution of delirium, it will never be plausible to suggest an avoidance of sedatives altogether, though haloperidol could provide a sedative sparing affect. The average amount of haloperidol administered in the MIND pilot was 17 mg/day over a median of 7 days on study drug, and this resulted in a median haloperidol plasma concentration of 4.5 ng/mL [IQR, 2.8-6.0].* Notably, the median [IQR] haloperidol plasma concentration in both the placebo and ziprasidone group was 0 ng/mL [0-0]. The protocol for MIND was similar to that of the MIND-USA trial. Treatment groups were balanced at enrollment by age, severity of illness, and admission diagnosis (#1 diagnosis being severe sepsis). The amount of sedatives given (benzodiazepines, propofol, and fentanyl) were balanced between groups over the course of the study. **Figure 2** shows that there were no differences in resolution of delirium and coma; other outcomes, such as length of stay, cognition at discharge, and survival, were similar between the three groups. It is important to emphasize that this *pilot feasibility study* was neither intended nor powered to detect differences in these outcomes. We used a modified Simpson-Angus Scale[164] to monitor objectively for EPS and checked an ECG at baseline and at 24 hours after start of study drug to detect QTc prolongation as well as cardiac arrhythmias. No differences in these anticipated study drug-related adverse events were noted between groups.

Figure 2. MIND Pilot Feasibility Trial

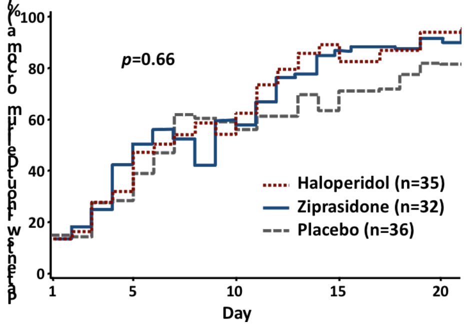

4.0 Study Objectives and Endpoints

4.1 Study Objectives. The primary objective of the MIND-USA study is to determine the efficacy and safety of haloperidol and ziprasidone vs. placebo in critically ill medical and surgical patients with delirium.

4.2 Efficacy Endpoints

4.2.1 Primary Endpoint. The primary endpoint of the trial will be **delirium/coma-free days (DCFDs)**, defined as the number of days alive without delirium or coma during the 14-day Treatment Period (from randomization until day 14 post-randomization). This continuous variable represents duration of time a patient is alive and free of acute brain dysfunction and has been used in other high impact studies.[61,165] The MIND-USA study will have tremendous implications for practice based on the outcome of the delirium duration (i.e., DCFD) results even if the secondary outcome of mortality is neutral between the two study groups. Thus, duration of time free of delirium and coma will be the key consideration of whether or not the study is considered positive or negative in the eyes of the medical community and the factor upon which the sample size is determined.

The analysis of this endpoint, as well as all primary analyses, will examine the **Intention-to-Treat (ITT) population**, defined as all patients who are randomized, regardless of receipt of study drug. Secondary analyses of patients who received at least one dose of study drug will also be conducted.

4.2.1 Secondary Endpoints

[1] 30-day, 90-day, and 1-year **survival (Aim 2)**.

[2] **ICU length of stay**, i.e., time to ICU discharge, represented by readiness for ICU discharge indicated by a physician order for transfer to lower level of care even if a bed availability problems prevent actual discharge from the ICU **(Aim 3)**.

[3] **Ventilator-free days** (VFDs), i.e., days alive and free of mechanical ventilation at 28 days.

[4] **Co-administration (frequency and quantity) of analgesics and sedatives** will be closely tracked to determine whether benefits of antipsychotics, if present, are due to sedative-sparing effects. Our MIND pilot study reported a trend towards reduced benzodiazepine exposure in the antipsychotic groups (haloperidol and ziprasidone) vs. placebo (p=0.10). Dexmedetomidine use will also be tracked because it is known to be associated with more DCFDs.[45]

[5] **Neuropsychological function** will be assessed using a telephone battery[166] to determine incidence, duration, and severity of cognitive impairment at 90 days and 1 year post-randomization **(Aim 4)**. The battery includes tests of memory, attention, reasoning, and executive function.

[6] **Quality of life, functional status (ADLs, IADLs), and psychological outcomes (PTSD)** at 90-days and 1-yearas determined by telephone follow-up. Follow-up testing will consist of ADL/IADL,[167 -168] CAM,[169] a neuropsychological battery,[166] EuroQOL quality of life (EQ-5D),[170-172] and PTSD/PCL.[173]

4.3 Safety Endpoints. Arrhythmias (including torsades de pointes), extrapyramidal symptoms, neuroleptic malignant syndrome, thromboembolism, and readmission (ICU and hospital) will be tracked.[37,114-122]

5.0 Inclusion/Exclusion Criteria

5.1 Inclusion Criteria. Patients will be eligible for inclusion in the Pre-Randomization Phase of the MIND-USA study (i.e., consented for data collection but not yet randomized because disease- or drug-induced coma prevents assessment for delirium or because delirium is not present) if they are [1] adult patients (≥ 18 years old) [2] in a medical and/or surgical ICU and [3] on mechanical ventilation or non-invasive positive pressure ventilation (NIPPV), and/or being treated for shock (e.g., with vasopressors or intra-aortic balloon pump). **Only patients who meet inclusion criteria, have no exclusion criteria (see section 5.2), and develop delirium (according to the CAM-ICU)[41,42] will advance to the Interventional Trial Phase (i.e., the randomization phase) of the MIND-USA study (see section 6.2).**

5.2 Exclusion Criteria. Patients will be excluded (i.e., not consented) for any of the following reasons:

[1] Rapidly resolving organ failure criteria, indicated by *planned immediate discontinuation* of mechanical ventilation, NIPPV, and/or vasopressors at the time of screening for study enrollment, such that the patient will no longer meet inclusion criteria.

[2] Pregnancy or breastfeeding (negative pregnancy test required prior to randomization for female patients of childbearing potential)

[3] Severe dementia or other chronic neurologic disease or disorder that either makes the patient incapable of living independently at baseline or results in an IQCODE ≥ 4.5 (completed by the patient or their qualified surrogate). Examples include but are not limited to mental illness requiring long-term institutionalization, acquired or congenital mental retardation, Parkinson's disease, Huntington's disease, Alzheimer's disease, and debilitating cerebrovascular disease. **Note**: Subjects with *mild to moderate dementia* will be enrolled in the study. The rationale for inclusion of these typically elderly patients is that they represent an important, expanding group of older ICU patients who are very high risk for a protracted delirium course, and only very sparse data exist to direct treatment for this expanding group of elders. In light of the recent black box warning regarding longer-term use of antipsychotics in patients with dementia, this trial will be an ideal mechanism by which to expand knowledge of the safety profile for short-term use of these medications to treat delirium in patients with mild to moderate dementia. Acknowledging that delirium and dementia likely share some pathogenetic mechanisms,[174,175] subgroup analyses on these patients will help generate hypotheses for future aging research.

[4] Acute or subacute severe neurologic deficit that is expected to make the patient incapable of living independently after hospital discharge due to cognitive deficits. Examples include stroke, intracranial hemorrhage, cranial trauma, intracranial malignancy, anoxic brain injury, and cerebral edema.

[5] History of torsades de pointes, documented baseline QT prolongation (congenital long QT syndrome), or QTc ≥ 550 ms at screening due to refractory electrolyte abnormalities or other refractory/uncorrectable etiologies.

[6] Ongoing maintenance therapy with typical or atypical antipsychotics or lithium[176] , with plans by the ICU team to continue the medication during the current ICU stay.

[7] History of neuroleptic malignant syndrome (NMS), haloperidol allergy, or ziprasidone allergy

[8] Expected death within 24 hours of enrollment or lack of commitment to aggressive treatment by family or the medical team (e.g., likely withdrawal of life support measures within 24 hours of screening)

[9] Inability to obtain informed consent from the patient or an authorized representative/surrogate for one of the following reasons:

  (a) Attending physician refusal
  (b) Patient and/or surrogate refusal
  (c) ~~Patient unable to consent and no surrogate available~~ [Note: No longer using this exclusion]
  (d) 72-hour period of eligibility was exceeded before the patient was screened
  (e) Non-comatose patient unable to consent and no surrogate available within 72 hours of meeting all inclusion criteria
  (f) Persistently comatose patient unable to consent and no surrogate available within 120 hours of meeting all inclusion criteria

When patients are unable to consent for themselves on enrollment, a consent/re-consent process will be used (surrogate consent at enrollment and re-consenting patients when competent), in keeping with recent literature on consenting delirious and/or ICU patients.[177,178]

[10] Blindness, deafness, or inability to speak or understand English, the latter only if at a center without Spanish-speaking research staff, since Spanish-speaking patients are eligible at sites that have Spanish-speaking research staff, these patients will not be followed in the long-term follow-up phase of the trial (see section 7.4) since the testing materials are primarily available only in English.

[11] Incarceration, since prisoners may be difficult to test during long-term follow-up

[12] Current enrollment in a study that does not allow co-enrollment or an interventional trial that uses delirium as a primary outcome

Special Note about Patients with a history of seizures. Subjects with a *history of seizures* on and off antiepileptics who meet inclusion criteria and no exclusion criteria will be eligible for enrollment into the MIND-USA study. These antipsychotics pose minimal risk of lowering the seizure threshold.[179-183] The presence of a seizure disorder in patients with schizophrenia or bipolar disorder is not a contraindication for use of haloperidol, ziprasidone, or other antipsychotics. Patients with a history, who are currently on therapy specifically for this indication, will be continued on antiepileptic medications and have the drug levels monitored by the medical team as indicated within usual practice.

6.0 Enrollment/Randomization

6.1. Screening and Obtaining Informed Consent (Pre-Randomization Phase). Study personnel at each site will screen every day using the ICU census. When an eligible patient is identified (i.e., inclusion criteria are met and no exclusion criteria are present), informed consent will be pursued; when obtained, the patient will be included in the Pre-Randomization Phase of the study, which will include approximately 1,300 participants. Surrogate consent will be required for most patients because during the initial phase of their illness they will often be either comatose or delirious. However, all patients consented via surrogate will be re-consented for participation in the trial once competent.[177,178]

6.2. Randomization (Interventional Trial Phase). Once informed consent is obtained, patients will be assessed twice daily for delirium using the CAM-ICU. When a patient becomes delirious (i.e., CAM-ICU positive), they will advance to the Interventional Trial Phase of the study and be assigned via randomization to one of the three treatment groups. Based on the most recent screening data from participating sites, we estimate 50%-60% of approximately 1,100 patients included in the pre-randomization phase will develop delirium, often after a short period of coma. We will thus be able to randomize 561 or more patients to participate in the Interventional Trial Phase, meeting our calculated sample size (see Section 11.1).

Randomization will be conducted in a 1:1:1 ratio to treatment with haloperidol, ziprasidone, or placebo using a computer-generated randomization scheme (with permuted block sizes of 6 and 8) stratified by study center and age (< 70 years vs. ≥ 70 years). The randomization scheme will be created by the study's primary biostatistician and will be distributed directly to the investigational pharmacy at each study site as a set of randomization lists stratified by study center and age (< 70 years vs. ≥ 70 years). Once a consented patient has become delirious and an order for blinded study drug is placed, the investigational pharmacist will refer to the appropriate randomization list (determined by the patient's age) to establish that patient's treatment assignment. The lists will only be accessible to investigational pharmacists, so that treatment assignment will be known only by the investigational pharmacists.

6.3 Blinding. Apart from the investigational pharmacists, all study personnel, patients, and clinicians will remain blinded to each patient's treatment assignment throughout enrollment, follow-up, and data analysis. If an AE is considered study-drug related, unexpected and serious, the study drug will be immediately discontinued and the event will be reviewed via the usual process, which may involve unblinded evaluation by the Clinical Monitor, as outlined in the Data Safety Monitoring Plan (see sections 9.2 & 9.3).

7.0 Study Procedures

7.1 Enrollment. Once informed consent is obtained, baseline data will be collected from two sources:

[1] The **medical records** will be used to collect demographics, preexisting conditions, home medications, and admission severity of illness and organ failure. If obtained as part of routine medical care, additional laboratory/test results will also be collected and may include sodium, creatinine, glucose and neuroimaging (e.g., CT and MRI).

[2] A **pre-hospital function assessment** via patient/surrogate interview will assess for tobacco and illicit drug use, an alcohol use disorder, and ascertain the patient's physical and cognitive abilities prior to the current hospitalization. Rather than relying on a "label" of dementia in the medical record, we will use the IQCODE.[184-186]

7.2 Pre-Randomization Phase. After informed consent is obtained, patients will be considered in the Pre-Randomization Phase until they either (a) become delirious according to the CAM-ICU and are thus randomized and enter the Interventional Trial Phase (see section 7.3) or (b) do not become delirious within 5 days of consent, develop or are found to have an exclusion criterion* (see section 5.2), die, or are discharged from the ICU, any of which will disqualify them from entering the Interventional Trial Phase. Patients who have been disqualified from randomization (i.e., the Interventional Trial Phase) and discharged from the ICU are eligible to be reenrolled in the trial if they are readmitted to the ICU and meet inclusion but no exclusion criteria. *Note: As an exception, patients who have resolution of organ failure criteria (e.g., discontinuation of mechanical ventilation, NIPPV, and/or vasopressors) during the Pre-Randomization Phase will remain eligible for randomization if they become delirious according to the CAM-ICU and are still in the ICU within 5 days of consent. Patients who do not progress from the Pre-Randomization phase to the Interventional Trial Phase—referred to as "Non-Randomized" patients—will not participate in the interventional trial, will not be randomized, and will not be included in the intention-to-treat population be categorized as Non-Interventional patients. In this case, the surrogate and/or patient and the medical team will be notified that the patient is not participating in the interventional trial.

During the Pre-Randomization Phase, the following daily data will be collected.

[1] The **medical records** will be used to collect vital signs and other data reflecting current severity of illness, recent and ongoing treatments (including medications and mechanical ventilator status), routine lab results, and complications, e.g., infections and device removals. These data will be generated as part of routine clinical care and will not require study-related tests.

[2] Direct **patient assessments** will occur twice daily (except when the participant or their surrogate decision maker refuses the assessment or the participant is unavailable, e.g., in the operating room for an extended period) to assess for pain using the Critical-Care Pain Observation Tool (CPOT), determine the level of sedation using the RASS,[187,188] and assess for delirium using the CAM-ICU.[41,42,189] Participants at select sites will be also assessed twice daily for signs/symptoms of catatonia using the Bush Francis Catatonia Rating Scale (BFCRS), a bedside evaluation used widely by psychiatrists to diagnose catatonia, and for motor symptoms of delirium using the Delirium Motor Subtype Scale (DMSS). Together, these two assessments take approximately 5 minutes to perform.

[3] A **bedside checklist** will be used to collect data regarding adherence to the nonpharmacologic ABCDE protocol (see section 7.2.1), which includes standardized components of ventilator weaning, sedation, and delirium management. Components of the ABCDE protocol will be marked as complete on a daily basis by ICU staff and/or study personnel after they are implemented.

[4] **Blood** (not exceeding 4 mL) will be collected to evaluate for genetic predictors of delirium, including but not limited to the apolipoprotein E4 polymorphism[175,190,191]

[5] **Urine** (10 mL) will be collected at select sites participating in an ancillary study to measure urinary cotinine and 4-(methylnitrosamino)-1-(3-pyridyl)-1-butanol (NNAL) to evaluate whether exposure to cigarette smoke increases delirium risk.

7.2.1 Nonpharmacologic (ABCDE) Protocol. Beginning in the Pre-Randomization Phase, we will standardize and/or track components of ICU care that may influence delirium risk via an evidence-based nonpharmacologic protocol referred to as the **ABCDE protocol**.[61,192-195] All study personnel will be well trained in the ABCDE protocol during the MIND-USA startup meeting, and compliance will be emphasized and tracked throughout the study. The local ICU nursing staff at each study center will also receive educational materials that will vary according to the needs of each site (though standardized template materials, including a pocket reference card, will be provided by the VCC); education will focus on the ABCDE protocol as well as on delirium recognition, risk factors, and prevention. Once a year through year 4 of the trial, the nurses in participating ICUs at each study site will be asked to complete a voluntary survey designed to identify practice habits, behaviors, attitudes, and perceptions regarding the ABCDE protocol. When knowledge gaps are identified or if compliance with the ABCDE protocol drops below 80%, additional education will be provided by study personnel. The ABCDE protocol has 3 components:

[1] **ABCDE (Awakening and Breathing Coordination)**. The ABC component is based on the Wake Up and Breathe protocol proven to improve outcomes, including one-year survival, in the Awakening and Breathing Controlled Trial.[61] The ABC component includes standardized spontaneous awakening trials[196] (i.e., daily interruption of sedation) paired with spontaneous breathing trials,[197] both administered only when specific safety criteria are met. All study centers use validated sedation scales to facilitate goal-directed sedation, a practice that will continue throughout the MIND-USA study. Ventilator management will be standardized according to each institution's approved protocols, including the use of low tidal volume ventilation for acute lung injury.[198]

[2] **ABCDE (Delirium monitoring and management)**. The Delirium component of the ABCDE protocol includes nonpharmacologic strategies that have been shown to reduce delirium in non-ICU settings.[68,199-208] Each day, study personnel will encourage members of the ICU team to perform the following tasks:

(a) Reorient and cognitively stimulate patients by conveying the day, date, place, and reason for hospitalization, updating whiteboards with caregiver names, requesting placement of a clock and calendar in the room, and discussing current events.[63,64,66,68,199-201,203,204,206]

(b) Determine need for hearing aids and/or eye glasses from the surrogate and request that the surrogate provide these to the patient when appropriate.[63,64,68,200,201,203,204,206]

(c) Monitor and manage pain in all patients using the CPOT or other assessments, in accordance with practice guidelines and local ICU policies.[19,63,209-213]

(d) Maintain sleep preservation using techniques including noise reduction strategies (e.g., minimize noise outside the room, offer white noise or earplugs), normalizing day-night variation in illumination, minimizing interruptions during normal sleeping hours via "time out" strategy, maintaining ventilator synchrony, and promoting comfort and relaxation (e.g.,

back care, massage, oral care, washing face/hands, and daytime bath).[64,200,201,203,204,214-219]

[3] **ABCDE (Early mobility and Exercise)**. The Exercise component of the ABCDE protocol will include strategies to promote mobility and exercise in the earliest phases of critical illness. Early physical/occupational therapy significantly reduced delirium duration for mechanically ventilated ICU patients in a recent randomized controlled trial.[192] Each day, study personnel will evaluate each patient's readiness for mobility and exercise and encourage or help to coordinate the following activities with the bedside nurse and physical/occupational therapist as appropriate: active range of motion in bed, sitting on the side of the bed, sitting in a bedside chair, standing in place, and ambulation.[63,64,66,192,200,203,220,221]

Barriers and Facilitators to the ABCDE bundle. At sites wishing to participate in this survey, critical care providers (i.e., nurses, respiratory therapists, pharmacists, physicians, occupational therapists [OT], and/or physical therapists [PT]) will be asked to complete a one-time voluntary electronic survey (i.e., the ABCDE Bundle Provider Survey) designed to identify barriers and facilitators to the use of the ABCDE bundle. This will replace the MIND-USA Nurse Survey at participating sites. In addition, managers (e.g., nurse manager, respiratory therapy manager, and/or PT/OT manager) will be asked to provide details about key organizational structures and processes that may influence a provider's ability to use the ABCDE bundle. Lastly, units will be observed for accessibility of equipment useful for applying the ABCDE bundle and unit layout. Both the manager evaluation and the unit observation will be accomplished utilizing the ABCDE Unit Observation and Manager Questionnaire. When barriers and facilitators are identified, this information will be provided to study personnel and/or managers at each site.

7.3 Interventional Trial Phase. Once noted to be delirious on one of the study CAM-ICU assessments and confirmed to have a QTc < 550 ms, patients will be randomized, advancing to the Interventional Trial Phase of the study, which will consist of 4 study periods: Treatment Period, Post-Study Drug Period, Discharge Period, and Long-Term Follow-Up Period. Upon entering the Interventional Trial Phase, each patient will be assigned via randomization (see section 6.2) to one of the three treatment groups, entering the Treatment Period until hospital discharge, death, or day 14 post-randomization (whichever occurs first). During the Treatment Period, patients will receive study drug while in the ICU and delirious per Study Drug Administration rules (see section 7.3.1).

7.3.1 Study Drug Administration. When a patient is diagnosed with delirium, randomization will be carried out by the investigational pharmacist at each study center according to the randomization scheme provided by the study's primary biostatistician. At each study center, the pharmacist will prepare and deliver study drug to the bedside nurse according to orders placed by study personnel. Study drugs, all clear liquids that are stable in a syringe for up to 24 hr at room temperature, will be delivered via titrated doses throughout the trial according to the administration rules in the table below:

Drug	Diluent	Equivalent Dose	Concentration
Haloperidol, USP	Provided in solution	5 mg	5 mg / mL
Ziprasidone 20 mg vial	Sterile water, 2.2 mL	10 mg	10 mg / mL
Placebo/Normal Saline, USP	Provided in solution	Placebo	Placebo

[1] **Route and Concentration**. All study drug will be administered intravenously (IV) by bolus over up to 5 minutes at concentrations of 5 mg/mL haloperidol or 10 mg/mL ziprasidone. Patients will only receive study drug while in the ICU and thus will be monitored with continuous telemetry as per usual ICU practice.

[2] **Dosing Range**. Study drug dose will be titrated in a double-blind manner according to clinical effect (see **Titration** section below). For patients in the haloperidol group, dose will range from 2.5mg (1.25 mg dose for patients ≥ 70 years old) to 10 mg of haloperidol IV q12 hours (maximum, 20mg/day), in keeping with numerous clinical practice guidelines.[19-21] This upper limit dose for haloperidol is below the threshold for excess D_2 receptor occupancy in order to avoid extrapyramidal side effects[78,106,109,111,222] and within the pharmacokinetic range established to achieve the pro-cognitive effects of the drug.[147] For patients in the ziprasidone group, dose will range from 5 mg (2.5 mg dose for patients ≥ 70 years old) to 20 mg of ziprasidone IV q12 hours (maximum, 40 mg/day) in keeping with the package insert for ziprasidone IM and recent case reports and case series of IV administration.[139,142]

[3] **Initiation**. A pre-randomization 12-lead ECG will be obtained prior to study drug initiation to assess QTc. If this ECG shows QTc ≥ 550 ms, randomization and study drug initiation will be delayed until reversible causes for the QTc prolongation are found and treated. When the QTc becomes < 550 ms, randomization may occur and study drug may be started. For those patients whose ECG shows QTc < 550 ms, the first dose of study drug will be administered immediately after delirium diagnosis and randomization at a dose of 2.5 mg haloperidol IV (or 1.25 mg for patients ≥ 70yrs old), 5 mg ziprasidone IV (or 2.5 mg for patients ≥ 70yrs old), or 0.5 mL placebo (or 0.25 mL for patients ≥ 70yrs old).

[4] **Titration**. After study drug initiation, subsequent study drug doses will be titrated twice daily—with all doses being delivered at approximately 10AM or 10PM—by doubling (increasing by 100%) or halving (decreasing by 50%) the volume according to the following rules. *Note: An exception to this schedule will occur if the initiation dose is given after 4PM, in which case the second study drug dose will be given approximately 6 hours after the initiation dose (rather than at 10PM).

(a) Titrating Up. If study drug is not at maximum dose, the volume will be doubled approximately every 12 hours until the maximum dose is reached (10 mg haloperidol/20 mg ziprasidone/2 mL placebo q12h) unless [1] the patient cannot be assessed with the CAM-ICU due to coma (see section 7.3.1.[4].(b) below) or [2] study drug has been restarted after being held for safety reasons (see section 7.3.1.[5] on Holding study drug). If study drug has been held and then restarted (see section 7.3.1.[5] on when study drug can be restarted after Holding), the volume will not be titrated up but will instead be maintained at half the dose that had been held as long as side effects do not recur.

(b) Maintaining Current Dose. If a patient cannot be assessed with the CAM-ICU due to coma that is drug-induced (i.e., RASS -4 or -5 while receiving sedatives), study drug will be maintained at the current volume. Note: Sedative-induced coma is not considered a safety reason to hold study drug, whereas study drug will be held if sedatives have been held > 12 hours and the patient is comatose (per section 7.3.1.[5].(c) below).

(c) Titrating Down. If a patient is determined to be CAM-ICU negative (i.e., not delirious) on two consecutive assessments, the study drug volume will be halved approximately every 12 hours to a minimum volume of 0.25 mL. When a patient has been CAM-ICU negative for 4 consecutive study CAM-ICU assessments, study drug will be discontinued regardless of the dose.

(d) Restarting Study Drug. If at any time during the Treatment Period a patient is noted to develop a new episode of delirium **and** is in the ICU setting, study drug will be restarted at the dose most recently administered, as long as study drug was not discontinued permanently for safety reasons (see **Permanent Discontinuation** section below). If study drug is restarted after being temporarily held for safety reasons (as described in section 7.3.1[5]), it will be restarted at half the dose that was being administered when the safety concern was noted and prompted the study drug to be held.

[5] **Holding**. Throughout the trial, study drug will be temporarily held for the following safety reasons:

(a) QTc Prolongation (≥ 550 ms). If a pre-dose 12-lead ECG (obtained per sections 7.3.1.[5].(a).(i) or (iii) below) confirms QTc ≥ 550 ms, study drug will be held. If a reversible cause for the QTc prolongation is found and treated and QTc becomes < 550 ms, study drug may be resumed at half the previous dose (not less than the minimum volume of 0.25 mL) as in our MIND pilot study[223] and other recent studies.[224] During the Interventional Trial Phase, QTc will be measured as follows:

(i) A pre-randomization 12-lead ECG will be obtained prior to study drug initiation.

(ii) Subsequently, QTc will be checked and documented prior to the administration of each dose of study drug, either manually calculated from a bedside telemetry strip using the Bazett formula (QTc = QT / √RR interval) or via software algorithms available in some bedside telemetry machines. Whenever these pre-dose measurements show a QTc ≥ 550 ms, a 12-lead ECG will be obtained to confirm that QTc ≥ 550 ms.

(b) Extrapyramidal symptoms (EPS). If the patient develops clinical EPS, including dystonia and/or symptoms detected by the modified Simpson-Angus Scale(see section 7.3.2.[2]), then the study drug will be held and the patient treated as clinically indicated by the ICU team, who will be notified of the EPS by study staff. Upon resolution of EPS, study drug will be resumed at half the previous dose (not less than the minimum of 0.25mL).

(c) Oversedation. If the patient is oversedated (i.e., > 1 RASS level deeper than ICU team's sedation target), two sequential steps will be taken: [1] **sedatives** (including analgesics being used for sedation) will be held or decreased for > 12 hours per the ICU team's standard protocol and study drug will be maintained at current dose or titrated per standard titration rules (see section 7.3.1.[4]), then [2] **study drug** will be held if sedatives have been held > 12 hours and the patient remains oversedated. In this case, study drug will be restarted at half the previous dose (not less than the minimum of 0.25 mL) when oversedation resolves (i.e., RASS is within 1 of the ICU team's target).

[6] **Permanent Discontinuation**. Study drug will be permanently discontinued at any time during the trial for any of the following safety reasons:
   - (a) Neuroleptic malignant syndrome (NMS)
   - (b) Torsades de pointes or another ventricular tachycardia (VT) that results in clinical sequelae (e.g., hypotension) and/or requires treatment
   - (c) New-onset coma due to a known structural brain disease such as stroke, intracranial hemorrhage, cranial trauma, malignancy, anoxic brain injury, or cerebral edema
   - (d) Drug reaction with eosinophilia and systemic symptoms (DRESS) syndrome
   - (e) Any other study drug-related, life-threatening, serious adverse event

7.3.2 Monitoring. The risks of haloperidol and ziprasidone include tardive dyskinesia, neuroleptic malignant syndrome (NMS), extrapyramidal side effects (EPS), torsades de pointes and other arrhythmias, and hyperglycemia. Throughout the Treatment Period, study personnel will carefully monitor all patients daily for evidence of these potential adverse effects, determine study drug efficacy and safety, and monitor other factors that may influence outcomes. The above section describes how study drug will be titrated, held, or permanently discontinued should adverse effects occur. Additionally, all patients will be monitored carefully throughout the Post-Study Drug Period, which includes the four full calendar days after study drug is discontinued. Note: For patients who receive no study drug after day 10 post-randomization, daily monitoring will conclude at the end of the Treatment Period (i.e., at hospital discharge, death, or day 14 post-randomization, whichever occurs first) because the Post-Study Drug Period for these patients will be contained within the Treatment Period. Alternatively, patients who continue to receive study drug after day 10 post-randomization will be monitored through the end of the Post-Study Drug Period, which will extend beyond the Treatment Period.

[1] **Efficacy**. To evaluate the efficacy of haloperidol and ziprasidone vs. placebo, and to guide titration of study drug dose (see section 7.3.1.[4]), all efficacy outcomes will be assessed during the Treatment Period or longer, depending on the outcome (see section 4.2). While patients are in the ICU and in the Treatment Period, study personnel will determine level of sedation using the RASS[187,188] and assess for delirium using the CAM-ICU twice daily (except when the participant or their surrogate decision maker refuses the assessment or the participant is unavailable, e.g., in the operating room for an extended period).[41,42,189] When patients are not in the ICU but still in the Treatment Period (i.e., in the hospital and prior to day 14 post-randomization), study personnel will assess them using the RASS and CAM-ICU once daily. In cases where the Post-Study Drug Period extends beyond the Treatment Period, the RASS and CAM-ICU will be completed once daily from the time the Treatment Period ends until the Post-Study Drug Period ends. Time-to-event outcomes (e.g., ICU length of stay, survival, etc.) will be assessed from the time of randomization (entry into the Interventional Trial Phase) until the outcome in question or a censoring event occurs.

[2] **Safety**. Patients will be monitored as part of routine ICU care for adverse reactions, including but not limited to NMS, acute dystonia (i.e., muscle spasms and contractions), pseudoparkinsonism, atrial and ventricular dysrhythmias, hyperglycemia, etc. Additionally, study personnel will specifically assess patients for the following safety outcomes.

(a) Extrapyramidal symptoms (EPS) according to a modified Simpson-Angus Scale[164] consisting of 5 items: elbow rigidity, wrist rigidity, glabella tap, tremor, and salivation
(b) Akathisia according to a 10 cm visual analog scale used on days that patients are not comatose or delirious
(c) QTc prolongation ≥ 550 ms as described in section 7.3.1.[5].(a)
(d) Hyperactive (i.e., agitated) delirium according to RASS[153] and Rescue Protocol use (see section 7.3.3)

[3] **Biological specimens**. On three occasions—at the time of randomization (i.e., Trial Day 0) and on Trial Days 1 and 2—20 mL of blood will be collected (max of 65 mL during study), processed, and stored at −80º C prior to being shipped to the Vanderbilt Coordinating Center biorepository, where it will be stored for batched analyses of the following:
(a) Haloperidol and ziprasidone plasma concentrations 10-60 minutes prior to morning doses of study drug on Trial Days 1 and 2
(b) Inflammatory/coagulopathic biomarkers, (e.g., IL-6, CRP, sTNFR1, protein C, and D-dimer) since some have hypothesized that a potential beneficial effect of haloperidol may be its anti-inflammatory properties[97,110,128]
(c) Exploratory biomarkers hypothesized to be associated with delirium and long-term cognitive impairment (LTCI) (e.g., markers of inflammation, coagulation, oxidative stress, amyloid beta, etc.) as well as genetic risk factors hypothesized to be associated with delirium and LTCI (e.g., APOE, single nucleotide polymorphisms [SNPs] associated with dementia, SNPs associated with sedative metabolism, etc.). Any additional biomarker analyses are to be determined by ongoing and future studies.

[4] **Delirium Experience and Cognitive Evaluation**. After resolution of delirium and prior to hospital discharge, patients will be asked to recall their delirium experience using the Delirium Experience Questionnaire (DEQ).[225,226] The DEQ assesses delirium recall and distress related to a delirium episode. Additionally, patients will complete a brief assessment of cognitive ability, the Montreal Cognitive Assessment (MoCA).[227,228]

[5] **Other data**. As in the Pre-Randomization Phase, the medical records will be used during the Intervention Trial Phase to collect vital signs and other data reflecting current severity of illness, recent and ongoing treatments (including medications and mechanical ventilator status), routine lab results, and complications, e.g., infections and device removals. These data will be generated as part of routine care and will not require study-related tests. In addition, participants at select sites will continue to be assessed with the BFCRS and DMSS twice daily until ICU discharge, then once daily until hospital discharge.

7.3.3 Rescue Protocol for Pain/Agitation/Delirium. Because half of delirious ICU patients have mixed (hypoactive with intermittent hyperactive) delirium[153,229,230] and the majority of critically ill patients will experience agitation at some point during their ICU stay,[231] a rescue protocol for the treatment of hyperactive delirium and/or agitation (which may be due to pain or anxiety as well as delirium) is an essential component of the ICU team's armamentarium. In order to effectively test the hypothesis, however, that antipsychotics improve outcomes for ICU patients with delirium, the administration of open-label antipsychotics must be dramatically

limited to ensure that patients are not exposed to antipsychotics except for what is administered as study drug. Due to the equipoise regarding the effects of antipsychotics in the ICU (see sections 2.3, 2.7, 3.3, and 3.5) and the importance of maintaining separation between treatment groups with respect to antipsychotic use, **the administration of open-label haloperidol, ziprasidone, or any other antipsychotic (except those prescribed specifically for nausea, such as compazine) will be restricted during the Treatment Period**. The Rescue Protocol described below is a 3-part evidence-based alternative to open-label antipsychotics for the management of hyperactive delirium/agitation based on current practice at participating study centers, clinical practice guidelines,[19-21] and other recent literature.[18,45,223,232-235] This rescue protocol prioritizes analgesia first, then sedation for patients displaying agitation/unsafe behavior, and then delirium treatment. All medications given will be recorded and analyzed by study group.

[1] **Pain/Analgesia**. Fentanyl or morphine (and, less often, hydromorphone) should be given as an IV bolus or a continuous infusion per the local ICU's protocol and/or the ICU team's preferences when pain is suspected or diagnosed (using the Critical-Care Pain Observation Tool [CPOT][212,213] or other assessments, per local ICU policies). Doses will be titrated to achieve adequate analgesia.

[2] **Agitation/Sedation**. If hyperactive delirium/agitation that poses a risk to the patient or ICU staff persists after adequate analgesics are delivered, sedation should be administered in the form of propofol via continuous infusion for patients with airway protection (endotracheal or tracheostomy tube). An alternative drug choice for patients without airway protection, dexmedetomidine via continuous infusion, should be considered. This sedative does not cause respiratory suppression. In centers where dexmedetomidine is not available, patients without airway protection who are agitated may be treated with benzodiazepines.

[3] **Delirium/Antipsychotics**. While in the ICU, all patients will be managed with the ABCDE nonpharmacologic protocol (which includes a Delirium component, see section 7.2.1) from the time consent is obtained until hospital discharge, death, or until the Treatment Period ends (whichever occurs first). If the patient is outside the ICU or the Treatment Period has ended, open-label antipsychotics can be administered for refractory delirium according to the ICU team's preference.

7.4 Long-Term Follow-Up Period. We will evaluate long-term outcomes among survivors, including neuropsychological function (**Aim 4**), quality of life, ADL/IADLs, PTSD, and caregiver burden approximately **3 months and 1 year** after randomization. Under the direction of the Vanderbilt Coordinating Center's lead neuropsychologist, trained study personnel will assess patients using the following validated telephone assessments:

[1] **Delirium** will be assessed via the "phone" Confusion Assessment Method[169] algorithm. Patients diagnosed with delirium will not be assessed further at that time, but contacted at weekly intervals and tested if/when delirium resolves.

[2] A **neuropsychological phone battery**[166] derived from standard cognitive tests and proven feasible and valid for phone use in a study of ICU survivors (with similar characteristics to those targeted for enrollment in the MIND-USA) will be used to assess memory, attention, reasoning, and executive functioning.

[3] **Activities of Daily Living (ADL)**[167] and **Instrumental ADLs**[168]

[4] **Quality of Life** will be assessed using the **EQ-5D**, a short, easy to administer, well-validated, and widely used instrument.[170-172]

[5] **Post-Traumatic Stress Disorder (PTSD)** will be assessed via the PTSD Checklist (PCL)[173]

[6] **Caregiver Burden** will be assessed via a caregiver burden interview, the caregiver burden checklist[236-238], and a brief caregiver employment questionnaire

[7] **Health care costs** and health status will be assessed via Medicare files

7.4.1 Retention of Patients for Long-Term Follow-Up. Throughout the trial, several methods will be employed to enhance patient retention through the end of the Long-Term Follow-Up Period. At enrollment and throughout the study, multiple contact addresses, phone numbers, and email addresses will be obtained to facilitate communication with participants during long-term follow-up. All phone conversations with participants will be characterized by study staff cheerfulness and respect for privacy, and these phone calls will be scheduled to accommodate participants' schedules. During each phone call, which will begin with an introduction, study staff will inquire about the participant's health, and express thanks for their willingness to participate. Participants will be compensated $50 after they complete the 3-month follow-up phone assessment and $50 after they complete the 12-month follow-up phone assessment.

7.5 Data Collection/Case Report Form Details. During all study phases, all data will be entered into electronic case report forms (eCRFs) in a secured password-protected database with the exception of the pre-hospital function assessment, the EQ-5D, the DEQ, the MoCA, and the neuropsychological phone battery, which will be collected on paper CRFs and later entered into the eCRFs for storage in the secured password-protected database. This study will utilize REDCap for data collection, transmission and storage. REDCap (Research Electronic Data Capture) is a secure, web-based application for building and managing online databases. Vanderbilt University, with collaboration from a consortium of institutional partners, including the Vanderbilt Institute for Clinical and Translation Research (VICTR) Informatics Core, developed and manages a software toolset and workflow methodology for electronic collection and management of research and clinical trial data. All study data will be entered via a password protected, study unique REDCap database website. REDCap servers are housed in a local data center at Vanderbilt and all web-based information transmission is encrypted. REDCap was developed specifically around HIPAA-Security guidelines and is recommended by both the Vanderbilt University Privacy Office and Institutional Review Board. REDCap has been disseminated for use locally at other institutions and currently supports > 140 academic/non-profit consortium partners and 11,000 research end-users (www.project-redcap.org).

During the Interventional Trial Phase daily data will be collected for up to 18 days or until hospital discharge or death, whichever occurs earlier. After study drug is discontinued, patients will be monitored for safety purposes during a 4-day Post-Study Drug Period; the duration of this period is chosen because the elimination half-life of haloperidol is such that all drug is expected to be eliminated within 4 days of the last dose; ziprasidone has a shorter elimination half-life than haloperidol. The Post-Study Drug Period may overlap with the Treatment Period, depending on the duration of study drug administration (which in turn depends on duration of delirium, ICU length of stay, side effects, etc., as described in section 7.3.1.) All patients will be monitored for at least 14 days after randomization unless discharge from the hospital or death occurs prior to

Trial Day 14, and those patients who receive more than 10 days of study drug will be monitored for up to 18 days after randomization, with monitoring occurring for 4 days after discontinuation of study drug. Additionally, data will be collected as it is generated, including but not limited to mechanical ventilation initiation and removal, ICU admissions and discharges, patient discharge assessments, and death will continue to be collected until hospital discharge. After hospital discharge patients will be evaluated at approximately 3 months and 1 year after randomization with the telephone neuropsychological battery described in section 7.4. In addition to the battery described, data will be collected regarding intervening events that happened since hospital discharge including, but not limited to, death and re-hospitalizations. Vital status at 12 months after randomization and date of death (if applicable) will be determined via medical records or the Social Security Death Index if not already known at hospital discharge. All signed Informed Consent Documents will be uploaded to the study database. This will allow the VCC ready access to review the consent forms for appropriate version use and completeness.

[continued on next page]

7.6 Schedule of Events

Variable* (abbreviations on next page)	Enrollment	Pre-Randomization Phase	Interventional Trial Phase			
			Treatment Period	Post-Study Drug Period†	Hospital Discharge	3 & 12 Month
Demographics, Comorbidities,[239] Neuroimaging (CT/MRI) if available	X					
AUDIT,[240] ADL,[167] IADL/FAQ,[168] IQCODE,[184-186] APACHE II,[241] tobacco and illicit drug use history, employment	X					
SOFA[242,243]	X	Daily	Daily	Daily		
Pregnancy test (Beta hCG)	X					
**Blood draw:** APOE genotyping	X					
**Urine collection:** cotinine and NNAL	X					
**Blood draw:** CPD, IL-6, CRP, D-Dimer, sTNFr1, protein-C			Days 0, 1, & 2			
**Study drug levels:** haloperidol & ziprasidone			Days 0, 1, & 2			
Hematology and Chemistry	X	Daily	Daily	Daily		
Co-administered psychoactive meds--sedative/analgesic/antipsychotic		Daily	Daily	Daily		
RASS (target/actual),[187,188] CAM-ICU[41,42] & CPOT[19,63,209-213]		2x daily	2x daily¶	Daily		
BFCRS & DMSS *at select participating site*s		2x daily	2x daily¶	Daily		
Hospital-acquired infections (in blood, urine, sputum)		Daily	Daily	Daily		
ABCDE protocol compliance‡		Daily	Daily			
12-lead ECG (baseline and subsequent ECGs)		X	See section 7.3.1.[5].(a)			
**Safety assessments:** Modified Simpson-Angus EPS scale,[164] visual analog akathisia scale, neuroleptic malignant syndrome, & dysrhythmia/QTc review			Daily	Daily		
Delirium Experience Questionnaire (DEQ)[225,226]					X	
Montreal Cognitive Assessment (MoCA)[227,228]					X	
Caregiver burden interview & checklist[236-238]						X
**Long-term telephone follow-up:** CAM,[169] neuro-psychological battery,[166] ADL,[167] IADL/FAQ,[168] EuroQOL quality of life (EQ-5D),[170-172] PTSD/PCL[173], employment						X
Health care costs and health status (via Medicare files)						X

Abbreviations (alphabetical)*: **ADL- activities of daily living, **APACHE II**- Acute Physiologic Chronic Health Evaluation II, **APOE**-apolipoprotein-E, **AUDIT**-Alcohol Use Disorders Identification Test, **BFCRS-** Bush Francis Catatonia Rating Scale, **CPOT**- Critical-Care Pain Observation Tool, **CAM**- Confusion Assessment Method, **CAM-ICU**- Confusion Assessment Method for ICU, **CRP**- C-reactive protein, **DMSS-** Delirium Motor Subtype Scale, **ECG**- electrocardiogram, **EPS**- extrapyramidal symptomatology, **FAQ**- Functional Activities Questionnaire for IADLs, **IADL**- instrumental activities of daily living, **IL-6**- interleukin 6, **IQCODE**- Informant Questionnaire of Cognitive Decline in Elderly, **NNAL**- 4-(methylnitrosamino)-1-(3-pyridyl)-1-butanol, **PCL**- PTSD checklist, **PTSD**- post traumatic stress disorder, **RASS**- Richmond Agitation Sedation Scale, **SOFA**- Sequential Organ Failure Assessment, **sTNFr1**- soluble TNF receptor 1

†*Post-Study Drug Period*: For patients who receive no study drug after day 10 post-randomization, daily monitoring will conclude at the end of the Treatment Period (i.e., at hospital discharge, death, or day 14 post-randomization, whichever occurs first) because the Post-Study Drug Period for these patients will be contained within the Treatment Period. Patients who continue to receive study drug after day 10 post-randomization will be monitored through the end of the Post-Study Drug Period, which will extend beyond the Treatment Period.

‡*ABCDE Protocol*: **A**wakening **B**reathing **C**oordination, **D**elirium monitoring/management, & **E**arly mobility/**E**xercise; NOTE: Each component of this standardized approach to patient care, referred to as the "nonpharmacologic delirium protocol," (see section 7.2.1) will be tracked daily in all patients once consented.

¶Unless the participant has been discharged from the ICU; non-ICU patients will be assessed 1x daily in the Treatment Period.

7.7 The Vanderbilt Coordinating Center (VCC). The VCC has extensive experience in the conduct of large, phase III clinical trials over the past decade and is comprised of over 50 people on-site at Vanderbilt. The VCC will perform (among other functions) the following: communicate with the FDA using a schedule of reporting in accordance with IND policies, design the database and data collection tool, establish a web-based data database, conduct startup meetings and site-training regarding protocol implementation and delirium monitoring to standardize all research activities during the trial, monitor enrollment pace and quality to ensure patients meet the inclusion/exclusion criteria, maintain blinding, track adverse events and ensure safety reporting, ensure protocol compliance, store plasma, serum, and genetic samples for planned and future analyses, conduct follow-up phone testing of neuropsychological function and quality of life via neuropsychology technicians, and work with the study centers and local study personnel using multiple proven patient retention techniques that have consistently achieved over 80% follow-up during previous studies.

8.0 Risks

8.1 Side Effects of Study Drug (Haloperidol and Ziprasidone). The risks of antipsychotics (haloperidol and ziprasidone) include tardive dyskinesia, neuroleptic malignant syndrome, extrapyramidal side effects (EPS), torsades de pointes and other arrhythmias, and hyperglycemia. Throughout the Interventional Trial Phase, study personnel will carefully monitor all patients for evidence of these potential adverse effects of the study drugs. These assessments are described above in section 7.3.2. Adverse events will be monitored closely and reported as described below in Section 9.0. Should any of these occur, adverse events will be reported to the coordinating center, with Serious Adverse Events reported within 24 hours. In addition, study drug will be titrated, held, or permanently discontinued, depending on the adverse effect, according to study protocol (see section 7.3.1). Both haloperidol and ziprasidone are pregnancy category C medications, and female patients of childbearing age must have a negative pregnancy test to meet entry criteria.

8.2 Risks from Blood Draws. All patients will have blood drawn for research purposes. Having blood drawn is commonly painful, and it can rarely lead to bleeding, bruising, or infection at the site of the blood draw. For this reason, it will be our standard approach whenever possible to obtain blood for research purposes through existing intravenous peripheral, central or arterial catheters since patients routinely have such catheters while in the ICU. The volume of blood collected for research specimens represents a small percentage of the amount of blood taken during the course of a standard ICU stay and will not represent a significant risk to the patient.

8.3 Steps Taken to Reduce Risks and Increase Impact of Study. We have taken the following steps in designing our protocol to minimize risk for the study population and to maximize the ultimate impact of this investigation on the field of medicine.[244]

[1] All interventions included in the MIND-USA study are supported by a well-grounded and clearly described rationale suggesting potential, though unproven, benefit for eligible patients.

[2] All interventions are common and with established equipoise within the context of usual care and considered good or competent care in light of an absence of clear proof in favor of one over the other.

[3] Experts in the fields of critical care, geriatrics, psychiatry, neuropsychology, nursing, pharmacology, and clinical trial design have developed the interventions being studied.

[4] The management of patients in all three treatment groups will be guided by explicit MIND-USA protocols so that the results of the trial can be clearly interpreted and imitated, where appropriate, in clinical practice. This will also allow for the use of the "superior group" as a control in future trials.

[5] The MIND-USA protocol will adjust study drug dose to meet individual patient needs in the attempt to deliver safe and effective care to critically ill older and younger patients. This protocol is explicitly designed and drafted from landmark trials as referenced to meet patients' needs over time and provide individualized care.

[6] The titration protocol for study drug and the rescue protocol for hyperactive delirium are both designed to minimize risks in comparison to anticipated benefits.

[7] Because no placebo-controlled trials of interventions for ICU delirium exist to direct our study design, we conducted the feasibility pilot studies described in sections 3.4 and 3.5 prior to proceeding with the design of this definitive phase III trial.

[8] An independent and qualified data safety monitoring board (DSMB) will be established (see section 9.3) to review the research protocol prior to the start of the study and conduct interim analyses for safety and review data on serious adverse events as close to real-time as possible.

[9] We have worked very closely with the FDA to make certain that this proposal is safe and that they agree with our planned oversight and approach to due diligence in monitoring and reviewing all adverse events during the conduct of the trial. We have an active and approved **FDA-IND #104322** for this 3-group study including both typical antipsychotic haloperidol and the atypical antipsychotic ziprasidone as well as placebo.

[10] Rigorous monitoring and reporting of prospectively defined Adverse Events (AEs) Serious Adverse Events (SAEs), and Suspected Unexpected Serious Adverse Reactions (SUSARs) will be conducted as outlined in section 9.2 to comprehensively monitor safety during the trial.[245]

9.0 Reporting of Adverse Events or Unanticipated Problems involving Risk to Participants or Others

A system has been established to report and track clinical outcomes and adverse events (AEs). Study personnel will monitor the safety of subjects and follow them until the event resolves or is explained.

9.1 Clinical Outcomes (not considered Adverse Events). In this study of critically ill patients who are at high risk for death or other adverse outcomes due to their underlying critical illness, clinical outcomes, including death and organ dysfunction, will be systematically tracked (collected in the CRF) and will be included as part of the safety and efficacy analyses for this study. For the purposes of reporting, death and organ dysfunction will not be recorded as adverse

events unless the investigator believes the event may have been caused by study drug and is more severe or prolonged than expected given the underlying critical illness (investigator's discretion). This approach—considering death and organ dysfunction as outcomes rather than adverse events and systemically tracking expected safety outcomes for analysis rather than solely recording individual adverse events—is common in ICU trials because these outcomes/events occur commonly in the ICU and this system mandates that data regarding death, organ dysfunction, and expected safety outcomes be tracked systematically for all patients and analyzed appropriately. All clinical outcomes will be systemically tracked throughout the Treatment Period and the Post-Study Drug Period as described in section 7.3.2. Listed below are events that will be tracked as clinical outcomes and will not therefore be required to be reported as adverse events during this study (unless believed to be study-drug related and more severe or prolonged than expected given the underlying critical illness):

[1] Death. All deaths occurring within the Interventional Trial Phase will be reported on the CRF in the death summary section. For deaths thought to be caused by study drug, a SUSAR (see section 9.2) will be reported along with the death summary.
[2] Respiratory failure, including need for mechanical ventilation (invasive or noninvasive) or episodes of hypoxemia
[3] Circulatory failure, including shock (whether requiring vasopressors or not) and cardiac arrhythmias, and hypertension
[4] Hepatic failure or injury leading to increased bilirubin
[5] Renal failure or injury leading to an increased creatinine or acute hemodialysis
[6] Coagulation derangements leading to thrombocytopenia
[7] Stroke and neuropsychological dysfunction that is believed to be newly acquired
[8] Alterations in vital signs (e.g., temperature, respiratory rate, oxygen saturation)
[9] Thromboembolisms
[10] ICU readmissions
[11] Infections
[12] Self-removal of devices and invasive tubing and/or monitoring equipment
[13] The following known adverse reactions to antipsychotics:
    (a) NMS
    (b) acute dystonia (i.e., muscle spasms and contractions)
    (c) pseudoparkinsonism
    (d) atrial and ventricular dysrhythmias
    (e) hyperglycemia
    (f) extrapyramidal symptoms (EPS)
    (g) tardive dyskinesia
    (h) akathisia
    (i) QTc prolongation ≥ 550 ms

9.2 Adverse Events (AEs), Serious Adverse Events (SAEs), and Serious Unexpected Suspected Adverse Reactions (SUSARs).An **Adverse Event (AE)** will be any untoward medical occurrence for a patient enrolled in the trial that is not tracked as a clinical outcome (see section 9.1), regardless of whether the event is considered study drug-related or not. If a subject experiences an AE after informed consent is signed but before receiving study drug, the event will be reported as an AE not study drug related. Prior to enrollment, study site personnel will note the occurrence and nature of each subject's medical condition(s). During the study, site

personnel will note changes in these condition(s) and/or the occurrence and nature of any AEs. All AEs occurring during the Treatment Period and/or the Post-Study Drug Period will be recorded on the CRF. This period is the window of time during which study drug-related adverse reactions are expected based on the duration of the study drug administration plus 4 additional days of Post-Study Drug Period (approximately 4-5 elimination half-lives of haloperidol, the longer acting of the two active study drugs). An AE that later meets criteria for an SUSAR between the start of study drug and hospital discharge will be reported as an SUSAR. If study drug is discontinued as a result of an AE, study personnel will document the circumstances leading to discontinuation of study drug.

All **AEs** in MIND-USA will be assessed as to whether they are (1) related to study drug, (2) serious, and/or (3) unexpected according to the following definitions:

9.2.1 Related. AEs that an investigator suspects are related to study drug receipt will be classified as **Suspected Adverse Reactions (SARs)**. Certainty of relatedness is not required as long as a reasonable possibility exists that the AE is related to study drug receipt.

9.2.2 Serious. AEs that result in any of the criteria below will be considered **Serious Adverse Events (SAEs)**:

[1] Death
[2] A life-threatening episode requiring immediate intervention
[3] Inpatient hospitalization or prolongation of existing hospitalization
[4] Persistent or significant incapacitation or substantial disruption of the ability to conduct normal life functions
[5] A congenital anomaly/birth defect
[6] An episode that requires intervention to prevent the above and/or permanent impairment or damage

9.2.3 Unexpected. AEs, including SARs, that are not listed in the investigator's brochure or are listed but are more severe or prolonged than expected based on the investigator's brochure will be considered Unexpected. In addition, AEs will be considered Unexpected even if they are mentioned in the investigator's brochure as known to be caused by the classes of drugs being studied or to be anticipated based on the pharmacological properties of the study drugs but not as specifically known to be caused by the study drugs.

As required by the FDA, all events that are **related,** **unexpected,** and **serious** will be reported as **Serious Unexpected Suspected Adverse Reactions (SUSARs).** The MIND-USA Study will monitor, track, and report all Clinical Outcomes and Adverse Events as required by regulatory bodies.

9.3 Communication and Reporting of Adverse Events with Coordinating Center and Regulatory Bodies. In order to ensure proper and timely reporting of all adverse events, there will be a clear communication plan for all sites to follow. Sites will be responsible for reporting all adverse events to the VCC; they will also follow their local IRB policies to determine when adverse events should be reported to the local IRB. The VCC will be responsible for reporting the events to the proper regulatory bodies (i.e., FDA, DSMB, and Coordinating Center IRB) in a timely manner and in communicating any responses from those bodies back to the sites. Site

IRBs will also get event reports according to site-specific IRB guidelines. Our procedures for reporting the various adverse events will be as follows:

[1] **AEs** and **SAEs** will be recorded in the patient's study chart in the electronic database and reported to the VCC within 5 days of occurrence. The VCC will provide a batched report of all study wide AEs annually to each regulatory body as part of the annual review process as required.

[2] **SUSARs** will be recorded in the patient's study chart in the electronic database and reported to the VCC within 24 hours of occurrence by telephone call. The VCC will report the event within 24 hours to the DSMB Chair and the Vanderbilt IRB. The DSMB Chair will serve as a real-time "Clinical Monitor" over all SUSARs by systematically reviewing SUSARs, evaluating relatedness of each event. When the DSMB Chair suspects an event is study-drug related, they will have the opportunity to access unblinded data in order to conduct appropriate safety monitoring. There should be no need for the site investigators to be unblinded since the study drug should have been stopped at the time of event occurrence per titrating rules of the study protocol. The DSMB Chair will work in concert with the rest of the DSMB to determine if any necessary actions need to occur as result of the event in order to increase the safety of the protocol. All SUSARs will be reported as required to the regulatory bodies, including the FDA and the full DSMB.

[3] Per the specific request of the DSMB, all occurrences of torsades de pointes and neuroleptic malignant syndrome (whether meeting the criteria of SUSAR or not) will be reported to the VCC within 24 hours of occurrence by telephone call. The VCC will report the event within 24 hours to the DSMB Chair.

9.4 Data Monitoring Plan. To ensure data is accurately and completely collected during the MIND-USA study, the VCC will follow a specific Data Monitoring Plan modeled after the FDA's guidelines for the monitoring of clinical investigations. Once each year, a VCC member will visit each study site to assure that the facilities continue to be acceptable for the purpose of the study, the study protocol is being followed, changes to the protocol have been approved by the local IRB, and the site investigator is carrying out the agreed-upon activities and has not delegated them to other unspecified staff. Also, the monitor will review randomized subject records to determine whether data collected is accurate, complete, and current. The monitor will also review disqualified subject records to determine whether the patient was accurately disqualified. Per the FDA guidelines, the monitor will compare *a representative number* of subject records and other supporting documents with the investigator's reports. Specifically, site visits will include the following:

[1] A **Technical Review** will occur annually and will consist of a VCC research nurse examining the quality and accuracy of data, regulatory documents and drug accountability. Data quality and accuracy will be reviewed through a CRF data and source document review. The monitor will randomly select three randomized subjects (or 10% of the randomized subjects enrolled since the last site visit, if more than 30 were enrolled in that time) to serve as a representative sample. In addition, the monitor will randomly select three disqualified subjects enrolled since the last site visit to confirm accuracy of disqualification. Regulatory Document Review will consist of a review of IRB approvals, informed consents, critical documents, and

protocols/amendments. The Drug Accountability review will focus on the accuracy and consistency of the investigational pharmacy records.

[2] A **Scientific Review** will occur at the discretion of the VCC as needed and could consist of presentations by the site staff on their organizational structure, patient recruitment, staff training, and quality control procedures. The site monitoring team will include a VCC research nurse as well as the MIND-USA Principal or Co-Investigator.

The site monitoring reports from these reviews will be submitted to the DSMB (Data and Safety Monitoring Board) and other regulatory bodies (IRB, FDA and NIH) as requested and/or required. Data accuracy reports (including site comparisons) as well as site monitoring updates will be presented to the VCC. This Monitoring Plan will serve as a method for identifying systematic problems and provide a means in which to institute resolution and follow-up and therefore increase data quality.

9.5 Data Safety Monitoring Board (DSMB)

[1] **Membership**. The DSMB includes 4 independent voting members (3 members will constitute a quorum) who are not study investigators and have no financial, scientific, or other conflict of interest with the trial; written documentation attesting to absence of conflict of interest will be required. Members include Roy Brower, MD (Chair), Professor of Medicine at Johns Hopkins University in Baltimore, Gordon Rubenfeld, MD, MSc, Professor of Medicine at Sunnybrook Health Sciences Centre in Toronto, David Schoenfeld, PhD, Biostatistician and Professor of Medicine at the Harvard School of Public Health in Boston, and Kate Payne, RN, JD, Director of Ethics & Palliative Care at Saint Thomas Hospital in Nashville. These members were recommended by the MIND-USA study PI and approved by the NIA. Also, Molly Wagster, PhD, Chief of the Behavioral & Systems Neuroscience Branch at the NIA will serve as an ex officio nonvoting member. Collectively, the DSMB has expertise in the following fields: **clinical geriatrics, critical care medicine, psychiatry, clinical trial methodology, and biostatistics**. Dr. Brower has agreed to serve as the Chair and will be responsible for overseeing meetings, developing agendas in consultation with the NIA and PI, and being the contact person for the DSMB.

[2] **Initial Meeting**. Prior to the initiation of the trial, the DSMB will meet and review the entire IRB-approved study protocol with regard to subject safety, recruitment, randomization, intervention, data management, quality control and analysis. If the protocol and other study documents are deemed satisfactory by the DSMB, they will recommend to the VCC that subject recruitment begin. If, alternatively, modifications to the protocol or other study documents are needed, the DSMB will recommend such modifications and postpone its recommendation to begin recruitment. This initial meeting may occur via conference call or in person and will begin with an introduction by the PI and VCC Co-Investigator, then continue as a closed session, including only DSMB members and (if available) NIA program staff.

[3] **Additional Meetings**. Throughout the study period, the DSMB will meet in person or by teleconference at least twice (at N=100 and N=300 patients randomized; see 11.5 for detailed description), with additional meetings when needed to review blinded or unblinded data as needed and appropriate, including data on recruitment, randomization, compliance, retention, protocol adherence, operating procedures, form

completion, intervention effects, gender and minority inclusion, and subject safety. The DSMB is responsible for identifying problems related to safety (including all SUSARs), requesting additional data relevant to safety (including all SUSARs), proposing analyses of safety endpoints as needed, and considering the rationale for continuation of the study in light of safety data, progress of randomization, retention, protocol adherence, and data management. Reports of AEs and SUSARs for the two interim looks at the data (at N=100 and N=300 patients randomized; see 11.5 for detailed description) will initially be provided to the DSMB in a blinded fashion (i.e., treatment group assignment will not be revealed), but the DSMB will retain the right to request an unblinded report. Only DSMB members will have access to unblinded data in order to preserve the integrity of data and minimize potential for bias while maintaining appropriate safety monitoring. After each DSMB meeting, the Chair will provide a written report to the VCC and the NIA program official. In addition, the VCC, in turn, will provide the reports to the Vanderbilt University IRB and to all sites for submission to their local IRBs.

10.0 Study Withdrawal/Discontinuation

Subjects may be withdrawn from study participation at the discretion of the investigator or if the patient/surrogate or attending physician requests that the subject be withdrawn. The reason and date of every withdrawal will be recorded. The Informed Consent Document will notify participants that their participation is voluntary, and they can tell the study staff at any time if they decide to stop participating. In addition, if they choose to withdraw their authorization for study staff to access protected health information (PHI) in the medical record, they may do so by notifying study staff in writing (the address is provided). If a participant chooses to no longer participate but does not notify study staff that they withdraw authorization for access to PHI, their medical record may be accessed to obtain outcomes and safety data. Follow-up will be performed for all discontinuations due to an AE or other safety concern until resolution, until deemed chronic and stable, or as long as clinically appropriate.

11.0 Statistical Considerations

11.1 Power Analyses and Sample Size Calculations for Delirium (Aim 1). Based on data collected during our BRAIN-ICU Study, we anticipate that patients in the control (placebo) group in MIND-USA will have a mean ± SD of 6.8±5.2 delirium/coma-free days during the 14-day Treatment Period. At a 2-sided significance level of 2.50%, after Bonferonni adjustment for 2 pairwise comparisons (each active treatment will be compared to placebo), a trial of 187 patients per treatment group (total N=561) will have 92% analytical power to detect a 2-day improvement in delirium/coma-free days. Importantly, this sample size will also provide 80% power to detect a 2.5-day improvement in DCFDs within 4 subgroups expected to be ~45% (N=252) of the study population: severe sepsis, age ≥ 65 years, high illness severity (APACHE II ≥ 25), and medical vs. surgical patients. We will also conduct a hypothesis-generating subgroup analysis for patients enrolled with preexisting cognitive impairment (~25% of study population, N=140).

11.2 Power analyses for Mortality and ICU LOS (Aims 2 and 3). Assuming 40% one-year mortality in the control (placebo) group, this study will have 80% power to detect a 14% absolute difference in one-year mortality at a 2-sided significance level of 2.50% (if control mortality is 50%, study has 80% power to detect 15% difference). For ICU length of stay (LOS), this study will have 80% power to detect a 2.43-day (29%) difference at a 2-sided significance level of 2.50% assuming an 8.2±7.6 day ICU LOS in controls.

11.3 Power Analyses for Long-Term Cognitive Impairment (Aim 4). We expect to follow ≥ 80% of survivors for evaluation of LTCI. Based on the expected mortality rates (see above), we expect an overall 31% mortality across all 3 groups and plan to test 310 (=561x0.69x0.80) patients for LTCI at 12 months. Analysis for this aim will use multivariable linear regression to account for potential confounders introduced by imbalances in groups created by death and loss to follow-up. With 310 patients, we will able to include independent variables with up to 21 degrees of freedom. The proposed study will have adequate—indeed abundant—ability to assess the independent effect of the intervention on cognitive impairment while controlling for confounders.

11.4 Data Analysis Plan. To assess success of randomization, the distribution of baseline factors, such as age, sex, race, severity of illness, and sepsis, will be assessed by comparing summary measures between patients randomized into the 3 groups to identify clinically meaningful differences rather than relying on statistical testing.[246] The primary outcome of the Aim 1 analysis is delirium/coma-free days, which we will examine using both unadjusted and adjusted analyses. To determine the unadjusted effect of antipsychotic administration on this and other continuous outcomes, we will utilize the Kruskal-Wallis test to compare delirium/coma-free days between the three treatment groups. If the test is significant, we will use pairwise Wilcoxon rank-sum tests to compare each of the two antipsychotics groups directly to the placebo group. To determine the unadjusted effect of antipsychotic administration on ICU length of stay, survival, and other time-to-event outcomes, the cumulative incidence probability of these outcomes will be estimated via Kaplan-Meier product limit method, and the log-rank test will be used to compare the three groups.

In addition to unadjusted analyses, we will also perform multiple regression to adjust for *a priori*-selected risk factors for outcomes, including age, severity of illness, admission diagnosis of sepsis, and duration of coma prior to randomization. Adjusting for strong risk factors of outcome variables is known to reduce measurement error, which may increase statistical power.[247] We will carefully assess differential effects of antipsychotic treatment by study sites by examining site-specific effects as well as by a formal interaction analysis using multiple regression. We will include study site as a random effect. For the analysis of delirium/coma-free days and other continuous outcomes, we will choose the type of multiple regression by carefully examining the distribution of the data. Cox proportional hazards regression[248] will be used to analyze the adjusted effect of antipsychotic treatment on time-to-event outcomes (including one-year survival), with censoring as appropriate. The proportionality assumption for Cox regression will be assessed graphically, using log-minus-log survival plot, and by adding interaction terms between each covariate and time in the model. The number of variables to be included in each regression analysis will be based on the rule of thumb that a multivariable regression model must include no more than m/10 parameters for it to be reliable on future similar patients, where m is the effective samples size (e.g., number events for Cox regression or number of patients for

proportional odds logistic regression).[249,250] This approach avoids overfitting and ensures robustness of regression parameters.

In addition to assessing whether study site modifies the effects of antipsychotic treatment on outcomes, as described above, we will also assess whether patient characteristics modify the effects of the interventions on outcomes to identify a subcategory of patients who may benefit more than others. For these analyses, we will develop multiple regression models that include interaction terms between study group and clinical characteristics, such as age, sepsis, and duration of coma prior to randomization. Nonlinearity of the association between continuous variables and outcomes will be assessed by inclusion of restricted cubic splines.

Regarding the analyses of all *a priori*-defined secondary outcomes described herein, no adjustments will be made for multiple comparisons in keeping with authoritative recommendations on this topic[251-253] and standard practice when analyzing multiple, prospectively defined outcomes in a clinical trial. As described in our proposal, each comparison made will be hypothesis-driven and based upon biological plausibility rather than exploratory in nature.

11.5 Interim Analysis. To ensure protocol compliance and safety within the MIND-USA study, we will conduct interim analyses that will allow early presentation of outcomes to the DSMB (see section 9.3). In addition to the final analysis of 561 patients, two interim analyses are planned at 100 patients and 300 patients randomized; these interim analyses will compare 90-day survival in the blinded treatment groups.

Using a modified Haybittle-Peto method, the MIND-USA Study should be terminated or modified (e.g., one group dropped) if the p value for the global test, which simultaneously compares the three treatment groups, is less than 0.0001 at any of the two interim analyses. If the trial continues to completion (N=561 randomized), a p value <0.0498 for the global test will be considered statistically significant to account for the two planned interim analyses. The DSMB will be provided data showing the effect size (relative risk) in order to put the p-values into clinical perspective.

11.6 Intention-to-Treat (ITT) Principle and Handling Missing Data. For all primary analyses, we will employ the intention-to-treat (ITT) principle (as defined in section 4.2.1). A secondary set of analyses will be conducted involving only patients who received at least one dose of study drug. One would expect that results of the primary and secondary would be nearly identical, yet in an ICU population, trials can sometimes experience an unexpected number of early and unexpected deaths. For outcome variables of Aims 1 through 3, missing data will be very low because the majority of data are collected during ICU stay. When missing data occur due to patient withdrawal, we will continue to follow patients in an observational setting to minimize missing outcome variables. When data cannot be collected, however, we will impute missing variables via multiple imputation methods, and the imputed variables will be included in the ITT analyses.[255] For Aim 4 (long-term cognitive impairment), our pilot work indicates that we can complete testing on 91% of survivors, yet we are powering the study conservatively for a lower follow-up rate in case we achieve 80% follow-up testing. Loss of survivors to follow-up is not random and may be associated with more severe cognitive or functional status deficits among those not tested. Losing these patients will, if anything, lead to underestimation of the overall degree of LTCI and likely bias towards the null. We will analyze whether particular baseline demographics, duration of delirium, or drug exposure are risk factors for failure to follow-up

with long-term cognitive testing. We will also conduct analyses using data from deceased and untested patients due to illness along observed LTCI scores with proportional odds logistic regression using previously developed[256] composite outcome as follows: 1 point: cognitively normal, 2 points: cognitively impaired, 3 points: un-able to test due to illness, 4 points: unable to test due to death. This method is popularly used in a research of long-term brain deficits to avoid survivor bias.[256]

12.0 Privacy/Confidentiality Issues

At no time will we reveal subject identities in any manner, whether in presentation, description or publication of the research for scientific purposes. All data obtained with subject or provider identifiers will be kept in locked file cabinets to ensure confidentiality, and all paper file contents will be shredded before disposal. All subjects will be assigned a unique study number for use in the computer database, and all electronic data will be kept in password-protected computer files to ensure confidentiality. All biological specimens (serum, plasma, and DNA samples), maintain for batched assay after trial completion, will be stored in a locked –80 °C freezer and labeled with study ID# only, without any patient identifiers. These samples will be accessible only to designated co-investigators. Results of the specified laboratory tests will be maintained in a password-protected database to be accessed only by designated co-investigators.

13.0 Follow-up and Record Retention

13.1 Duration of Record Retention. Information stored in the database will be stored for an indefinite period of time for future reference, including for use in subsequent data analyses. Throughout the study, all collected data will be entered directly in to the secure password-protected web-based database.

13.2 Method for Destruction or Indefinite Archival of Information. Biological specimens will be stored for an indefinite period of time for use in future studies (as described in the informed consent document). Because genetic markers of risk for delirium and other study outcomes are not currently known, storage of specimens for later research use is required to advance this knowledge of ICU delirium. It is our hope that polymorphisms will eventually be identified that will help us better understand the pathogenesis of delirium and patients' response (or lack thereof) to therapy. When such information is available, it would be invaluable to have a bank of genetic samples available for testing that can be coupled with a comprehensive set of clinical data such as the database being collected in this investigation. Consent for use of genetic samples will be obtained from patients and/or surrogates at the local sites. In the event that the permission to use the genetic sample is incomplete and the patient has already been discharged from the hospital, study coordinators from the VCC will pursue this permission from study participants. This contact may occur outside of the 12 month follow-up study period.

As stated in the informed consent document, participants' consent to use or share PHI does not expire unless study staff are explicitly notified of this decision in writing from the participant (even in the event that a subject withdraws from further participation). If a subject chooses to withdraw their authorization for study staff to access PHI and/or to have stored data/specimens destroyed, they may do so by notifying study staff in writing (the address is

provided). In this case, actions will be taken to ensure that the data is properly destroyed and that the appropriate documentation is maintained as outlined in standard operating procedures.

13.3 Duration of Study. This study is planned to last a total of 5 years. Patient recruitment and follow-up will take place over 4 years and 3 months. Throughout the recruitment and testing phases, we will diligently monitor data collection, perform data cleaning, and hold conference calls and in-person meetings with study personnel to ensure protocol compliance and overall efficiency of study implementation. We will also work regularly with our statisticians to execute the planned analyses. The final analyses will occur during the last 6 months of the study period, when monitoring, quality checks, archiving and manuscript preparation will take place.

14.0 References

(1) Halpern NA, Pastores SM, Greenstein RJ. Critical care medicine in the United States 1985-2000: an analysis of bed numbers, use, and costs. Crit Care Med 2004; 32:1254-1259.
(2) Angus DC, Kelley MA, Schmitz RJ, et al. Caring for the critically ill patient. Current and projected workforce requirements for care of the critically ill and patients with pulmonary disease: can we meet the requirements of an aging population? JAMA 2000; 284:2762-2770.
(3) Levkoff SE, Evans DA, Liptzin B, et al. Delirium. The occurrence and persistence of symptoms among elderly hospitalized patients. Arch Intern Med 1992; 152:334-340.
(4) Marcantonio ER, Simon SE, Bergmann MA, et al. Delirium symptoms in post-acute care: prevalent, persistent, and associated with poor functional recovery. J Am Geriatr Soc 2003; 51:4-9.
(5) Cole MG, Ciampi A, Belzile E, et al. Persistent delirium in older hospital patients: a systematic review of frequency and prognosis. Age Ageing 2009; 38:19-26.
(6) Pisani MA, Murphy TE, Araujo KL, et al. Benzodiazepine and opioid use and the duration of intensive care unit delirium in an older population. Crit Care Med 2009; 37:177-183.
(7) Ely EW, Shintani A, Truman B, et al. Delirium as a predictor of mortality in mechanically ventilated patients in the intensive care unit. JAMA 2004; 291:1753-1762.
(8) Lin SM, Liu CY, Wang CH, et al. The impact of delirium on the survival of mechanically ventilated patients. Crit Care Med 2004; 32:2254-2259.
(9) Pisani MA, Kong SY, Kasl SV, et al. Days of delirium are associated with 1-year mortality in an older intensive care unit population. Am J Respir Crit Care Med 2009; 180:1092-1097.
(10) Ely EW, Gautam S, Margolin R, et al. The impact of delirium in the intensive care unit on hospital length of stay. Intensive Care Med 2001; 27:1892-1900.
(11) Thomason JW, Shintani A, Peterson JF, et al. Intensive care unit delirium is an independent predictor of longer hospital stay: a prospective analysis of 261 non-ventilated patients. Crit Care 2005; 9:R375-R381.
(12) Milbrandt EB, Deppen S, Harrison PL, et al. Costs associated with delirium in mechanically ventilated patients. Crit Care Med 2004; 32:955-962.

(13) Jackson JC, Gordon SM, Hart RP, et al. The association between delirium and cognitive decline: a review of the empirical literature. Neuropsychol Rev 2004; 14:87-98.
(14) Girard TD, Jackson JC, Pandharipande PP, et al. Delirium as a predictor of long-term cognitive impairment in survivors of critical illness. Crit Care Med 2010; 38:1513-1520.
(15) Pisani MA, Murphy TE, Van Ness PH, et al. Characteristics associated with delirium in older patients in a medical intensive care unit. Arch Intern Med 2007; 167:1629-1634.
(16) Pandharipande P, Cotton BA, Shintani A, et al. Prevalence and risk factors for development of delirium in surgical and trauma intensive care unit patients. J Trauma 2008; 65:34-41.
(17) Lat I, McMillian W, Taylor S, et al. The impact of delirium on clinical outcomes in mechanically ventilated surgical and trauma patients. Crit Care Med 2009; 37:1898-1905.
(18) Riker RR, Shehabi Y, Bokesch PM, et al. Dexmedetomidine vs midazolam for sedation of critically ill patients: a randomized trial. JAMA 2009; 301:489-499.
(19) Jacobi J, Fraser GL, Coursin DB, et al. Clinical practice guidelines for the sustained use of sedatives and analgesics in the critically ill adult. Crit Care Med 2002; 30:119-141.
(20) Borthwick M, Bourne R, Craig M, Egan A, Oxley J, for the United Kingdom Clinical Pharmacy Association. Detection, prevention and treatment of delirium in critically ill patients. United Kingdom Clinical Pharmacy Association 2006. (Accessed January 25, 2010 at http://www.ics.ac.uk/intensive_care_professional/ukcpa_delirium_2006.)
(21) Young J, Anderson D, Gager M, George J, Healy J, Harvey.W., Hicks A, Holmes J, Ouldred E, Siddiqi N, Sturmey G, Tabernacle B, White R, Wiltshire M, Clegg A, for the National Institute for Health and Clinical Excellence. Delirium: diagnosis, prevention and management. National Institute for Health and Clinical Excellence 2010. (Accessed January 19, 2010 at http://www.nice.org.uk/nicemedia/pdf/DeliriumDraftFullGuideline061109.pdf.)
(22) Inouye SK, Viscoli CM, Horwitz RI, et al. A predictive model for delirium in hospitalized elderly medical patients based on admission characteristics. Ann Intern Med 1993; 119:474-481.
(23) Marcantonio ER, Goldman L, Mangione CM, et al. A clinical prediction rule for delirium after elective noncardiac surgery. JAMA 1994; 271:134-139.
(24) Martin J, Heymann A, Basell K, et al. Evidence and consensus-based German guidelines for the management of analgesia, sedation and delirium in intensive care - short version. Ger Med Sci 2010; 8:Doc02.
(25) Lacasse H, Perreault MM, Williamson DR. Systematic review of antipsychotics for the treatment of hospital-associated delirium in medically or surgically ill patients. Ann Pharmacother 2006; 40:1966-1973.
(26) Seitz D, Gill SS. Perioperative haloperidol to prevent postoperative delirium. J Am Geriatr Soc 2006; 54:861.
(27) Lonergan E, Britton AM, Luxenberg J, et al. Antipsychotics for delirium. Cochrane Database Syst Rev 2007;CD005594.
(28) Campbell N, Boustani MA, Ayub A, et al. Pharmacological management of delirium in hospitalized adults--a systematic evidence review. J Gen Intern Med 2009; 24:848-853.
(29) Patel RP, Gambrell M, Speroff T, et al. Delirium and sedation in the intensive care unit: survey of behaviors and attitudes of 1384 healthcare professionals. Crit Care Med 2009; 37:825-832.

(30) Mac Sweeney R, Barber V, Page V, et al. A national survey of the management of delirium in UK intensive care units. QJM 2010; 103:243-251.
(31) Nasrallah HA, White T, Nasrallah AT. Lower mortality in geriatric patients receiving risperidone and olanzapine versus haloperidol: preliminary analysis of retrospective data. Am J Geriatr Psychiatry 2004; 12:437-439.
(32) Kuehn BM. FDA warns antipsychotic drugs may be risky for elderly. JAMA 2005; 293:2462.
(33) Schneider LS, Dagerman KS, Insel P. Risk of death with atypical antipsychotic drug treatment for dementia: meta-analysis of randomized placebo-controlled trials. JAMA 2005; 294:1934-1943.
(34) Wang PS, Schneeweiss S, Avorn J, et al. Risk of death in elderly users of conventional vs. atypical antipsychotic medications. N Engl J Med 2005; 353:2335-2341.
(35) Gill SS, Bronskill SE, Normand SL, et al. Antipsychotic drug use and mortality in older adults with dementia. Ann Intern Med 2007; 146:775-786.
(36) Ballard C, Hanney ML, Theodoulou M, et al. The dementia antipsychotic withdrawal trial (DART-AD): long-term follow up on a randomised placebo-controlled trial. Lancet Neurology 2009; 8:151-157.
(37) Ray WA, Chung CP, Murray KT, et al. Atypical antipsychotic drugs and the risk of sudden cardiac death. N Engl J Med 2009; 360:225-235.
(38) National Research Council Committee on Future Directions for Cognitive Research on Aging. The Aging Mind: Opportunities in Cognitive Research. Washington, D.C.: National Academy Press, 2000.
(39) Schneeweiss S, Avorn J. Antipsychotic agents and sudden cardiac death--how should we manage the risk? N Engl J Med 2009; 360:294-296.
(40) Ely EW, Stephens RK, Jackson JC, et al. Current opinions regarding the importance, diagnosis, and management of delirium in the intensive care unit: a survey of 912 healthcare professionals. Crit Care Med 2004; 32:106-112.
(41) Ely EW, Margolin R, Francis J, et al. Evaluation of delirium in critically ill patients: validation of the Confusion Assessment Method for the Intensive Care Unit (CAM-ICU). Crit Care Med 2001; 29:1370-1379.
(42) Ely EW, Inouye SK, Bernard GR, et al. Delirium in mechanically ventilated patients: validity and reliability of the confusion assessment method for the intensive care unit (CAM-ICU). JAMA 2001; 286:2703-2710.
(43) McNicoll L, Pisani MA, Zhang Y, et al. Delirium in the intensive care unit: occurrence and clinical course in older patients. J Am Geriatr Soc 2003; 51:591-598.
(44) Girard TD, Carson SS, Pandharipande PP, et al. The modifying the incidence of delirium (MIND) trial: a randomized controlled trial of the feasibility, efficacy, and safety of antipsychotics for the prevention and treatment of ICU delirium. Am J Respir Crit Care Med 177, A817. 2008.
(45) Pandharipande PP, Pun BT, Herr DL, et al. Effect of sedation with dexmedetomidine vs lorazepam on acute brain dysfunction in mechanically ventilated patients: the MENDS randomized controlled trial. JAMA 2007; 298:2644-2653.
(46) American Psychiatric Association. Diagnostic and statistical manual of mental disorders. Fourth edition, text revision. Washington, DC.: American Psychiatric Association, 2000.

(47) Morandi A, Pandharipande P, Trabucchi M, et al. Understanding international differences in terminology for delirium and other types of acute brain dysfunction in critically ill patients. Intensive Care Med 2008; 34:1907-1915.
(48) Jackson JC, Hart RP, Gordon SM, et al. Six-month neuropsychological outcome of medical intensive care unit patients. Crit Care Med 2003; 31:1226-1234.
(49) Jackson JC, Obremskey W, Bauer R, et al. Long-term cognitive, emotional, and functional outcomes in trauma intensive care unit survivors without intracranial hemorrhage. J Trauma 2007; 62:80-88.
(50) Hopkins RO, Jackson JC. Assessing neurocognitive outcomes after critical illness: are delirium and long-term cognitive impairments related? Curr Opin Crit Care 2006; 12:388-394.
(51) Hopkins RO, Weaver LK, Pope D, et al. Neuropsychological sequelae and impaired health status in survivors of severe acute respiratory distress syndrome. Am J Respir Crit Care Med 1999; 160:50-56.
(52) Hopkins RO, Weaver LK, Collingridge D, et al. Two-Year Cognitive, Emotional, and Quality-of-Life Outcomes in Acute Respiratory Distress Syndrome. Am J Respir Crit Care Med 2005; 171:340-347.
(53) Nelson JE, Tandon N, Mercado AF, et al. Brain dysfunction: another burden for the chronically critically ill. Arch Intern Med 2006; 166:1993-1999.
(54) Inouye SK. Delirium in older persons. N Engl J Med 2006; 354:1157-1165.
(55) Ringdal M, Plos K, Ortenwall P, et al. Memories and health-related quality of life after intensive care: a follow-up study. Crit Care Med 2010; 38:38-44.
(56) van den Boogaard M, Schoonhoven L, Roodbol G, et al. Successful implementation of a delirium assessment tool on the ICU. Intensive Care Medicine 34, S130. 2008.
(57) Page VJ, Navarange S, Gama S, et al. Routine delirium monitoring in a UK critical care unit. Crit Care 2009; 13:R16.
(58) van den BM, Pickkers P, van der HH, et al. Implementation of a delirium assessment tool in the ICU can influence haloperidol use. Crit Care 2009; 13:R131.
(59) Ray WA, Meredith S, Thapa PB, et al. Antipsychotics and the risk of sudden cardiac death. Arch Gen Psychiatry 2001; 58:1161-1167.
(60) Ray WA, Taylor JA, Meador KG, et al. Reducing antipsychotic drug use in nursing homes. A controlled trial of provider education. Arch Intern Med 1993; 153:713-721.
(61) Girard TD, Kress JP, Fuchs BD, et al. Efficacy and safety of a paired sedation and ventilator weaning protocol for mechanically ventilated patients in intensive care (Awakening and Breathing Controlled trial): a randomised controlled trial. Lancet 2008; 371:126-134.
(62) Skrobik YK, Bergeron N, Dumont M, et al. Olanzapine vs haloperidol: treating delirium in a critical care setting. Intensive Care Med 2004; 30:444-449.
(63) Marcantonio ER, Flacker JM, Wright RJ, et al. Reducing delirium after hip fracture: a randomized trial. J Am Geriatr Soc 2001; 49:516-522.
(64) Inouye SK, Bogardus ST, Jr., Charpentier PA, et al. A multicomponent intervention to prevent delirium in hospitalized older patients. N Engl J Med 1999; 340:669-676.
(65) Bogardus ST, Jr., Desai MM, Williams CS, et al. The effects of a targeted multicomponent delirium intervention on postdischarge outcomes for hospitalized older adults. Am J Med 2003; 114:383-390.

(66) Pitkala KH, Laurila JV, Strandberg TE, et al. Multicomponent geriatric intervention for elderly inpatients with delirium: a randomized, controlled trial. J Gerontol A Biol Sci Med Sci 2006; 61:176-181.
(67) Rizzo JA, Bogardus ST, Jr., Leo-Summers L, et al. Multicomponent targeted intervention to prevent delirium in hospitalized older patients: what is the economic value? Med Care 2001; 39:740-752.
(68) Milisen K, Lemiengre J, Braes T, et al. Multicomponent intervention strategies for managing delirium in hospitalized older people: systematic review. J Adv Nurs 2005; 52:79-90.
(69) Trzepacz PT. Update on the neuropathogenesis of delirium. Dement Geriatr Cogn Disord 1999; 10:330-334.
(70) Gunther ML, Morandi A, Ely EW. Pathophysiology of delirium in the intensive care unit. Crit Care Clin 2008; 24:45-65.
(71) Van Der Mast RC. Delirium: the underlying pathophysiological mechanisms and the need for clinical research. J Psychosom Res 1996; 41:109-113.
(72) Tune LE, Egeli S. Acetylcholine and delirium. Dement Geristr Cogn Disord 1999; 10:342-344.
(73) Hshieh TT, Fong TG, Marcantonio ER, et al. Cholinergic deficiency hypothesis in delirium: a synthesis of current evidence. J Gerontol A Biol Sci Med Sci 2008; 63:764-772.
(74) Baldessarini RJ, Tarazi FI. Drugs and the Treatment of Psychiatric Disorders. In: Hardman JG, Limbird LE, editors. Goodman & Gilman's The Pharmacological Basis of Therapeutics. McGraw-Hill, 2001: 485-520.
(75) Schotte A, Janssen PF, Gommeren W, et al. Risperidone compared with new and reference antipsychotic drugs: in vitro and in vivo receptor binding. Psychopharmacology (Berl) 1996; 124:57-73.
(76) Seamans JK, Floresco SB, Phillips AG. D1 receptor modulation of hippocampal-prefrontal cortical circuits integrating spatial memory with executive functions in the rat. J Neurosci 1998; 18:1613-1621.
(77) Mrzljak L, Goldman-Rakic PS. Acetylcholinesterase reactivity in the frontal cortex of human and monkey: contribution of AChE-rich pyramidal neurons. J Comp Neurol 1992; 324:261-281.
(78) Wilson JM, Sanyal S, Van Tol HH. Dopamine D2 and D4 receptor ligands: relation to antipsychotic action. Eur J Pharmacol 1998; 351:273-286.
(79) Broderick PA, Gibson GE. Dopamine and serotonin in rat striatum during in vivo hypoxic-hypoxia. Metab Brain Dis 1989; 4:143-153.
(80) Breitbart W, Strout D. Delirium in the terminally ill. Clinics in Geriatric Medicine 16, 357-372. 2000.
(81) Platt MM, Breitbart W, Smith M, et al. Efficacy of neuroleptics for hypoactive delirium. J Neuropsychiatry Clin Neurosci 1994; 6:66-67.
(82) Wilkinson LS. The nature of interactions involving prefrontal and striatal dopamine systems. J Psychopharmacol 1997; 11:143-150.
(83) Dubois MJ, Bergeron N, Dumont M, et al. Delirium in an intensive care unit: a study of risk factors. Intensive Care Med 2001; 27:1297-1304.
(84) Van Rompaey B, Schuurmans MJ, Shortridge-Baggett LM, et al. Risk factors for intensive care delirium: a systematic review. Intensive Crit Care Nurs 2008; 24:98-107.

(85) Aldemir M, Ozen S, Kara IH, et al. Predisposing factors for delirium in the surgical intensive care unit. Crit Care 2001; 5:265-270.
(86) Pandharipande P, Shintani A, Peterson J, et al. Lorazepam is an independent risk factor for transitioning to delirium in intensive care unit patients. Anesthesiology 2006; 104:21-26.
(87) American Psychiatric Association. Practice guideline for the treatment of patients with delirium. Am J Psychiatry 1999; 156:1-20.
(88) Shapiro BA, Warren J, Egol AB, et al. Practice parameters for sustained neuromuscular blockade in the adult critically ill patient: an executive summary. Society of Critical Care Medicine. Crit Care Med 1995; 23:1601-1605.
(89) Seitz DP, Gill SS, van Zyl LT. Antipsychotics in the treatment of delirium: a systematic review. J Clin Psychiatry 2007; 68:11-21.
(90) Sanders KM, Stern TA, O'Gara PT, et al. Delirium during intra-aortic balloon pump therapy. Incidence and management. Psychosomatics 1992; 33:35-44.
(91) Riker R, Fraser G, Cox P. Continuous infusion of haloperidol controls agitation in critically ill patients. Crit Care Med 1994; 22:433-440.
(92) Levenson JL. High-dose intravenous Haloperidol for agitated delirium following lung transplantation. Psychosomatics 1995; 36:66-68.
(93) Seneff MG, Mathews RA. Use of haloperidol infusions to control delirium in critically ill adults. Ann Pharmacother 1995; 29:690-693.
(94) Caplan JP. Haloperidol and delirium: management or treatment? Crit Care Med 2009; 37:354-355.
(95) Breitbart W, Marotta R, Platt MM, et al. A double-blind trial of haloperidol, chlorpromazine, and lorazepam in the treatment of delirium in hospitalized AIDS patients. Am J Psychiatry 1996; 153:231-237.
(96) Kalisvaart KJ, de Jonghe JF, Bogaards MJ, et al. Haloperidol prophylaxis for elderly hip-surgery patients at risk for delirium: a randomized placebo-controlled study. J Am Geriatr Soc 2005; 53:1658-1666.
(97) Milbrandt EB, Kersten A, Kong L, et al. Haloperidol use is associated with lower hospital mortality in mechanically ventilated patients. Crit Care Med 2005; 33:226-229.
(98) Boettger S, Breitbart W. Atypical antipsychotics in the management of delirium: a review of the empirical literature. Palliat Support Care 2005; 3:227-237.
(99) Han CS, Kim YK. A double-blind trial of risperidone and haloperidol for the treatment of delirium. Psychosomatics 2004; 45:297-301.
(100) Lee KU, Won WY, Lee HK, et al. Amisulpride versus quetiapine for the treatment of delirium: a randomized, open prospective study. Int Clin Psychopharmacol 2005; 20:311-314.
(101) Maneeton B, Maneeton N, Srisurapanont M. An open-label study of quetiapine for delirium. J Med Assoc Thai 2007; 90:2158-2163.
(102) Pae CU, Lee SJ, Lee CU, et al. A pilot trial of quetiapine for the treatment of patients with delirium. Hum Psychopharmacol 2004; 19:125-127.
(103) Prakanrattana U, Prapaitrakool S. Efficacy of risperidone for prevention of postoperative delirium in cardiac surgery. Anaesth Intensive Care 2007; 35:714-719.
(104) Devlin JW, Roberts RJ, Fong JJ, et al. Efficacy and safety of quetiapine in critically ill patients with delirium: a prospective, multicenter, randomized, double-blind, placebo-controlled pilot study. Crit Care Med 2010; 38:419-427.

(105) Devlin JW, Roberts RJ, Fong JJ, et al. Efficacy and safety of quetiapine in critically ill patients with delirium: a prospective, multicenter, randomized, double-blind, placebo-controlled pilot study. Crit Care Med 2010; 38:419-427.
(106) Kapur S, Seeman P. Antipsychotic agents differ in how fast they come off the dopamine D2 receptors. Implications for atypical antipsychotic action. J Psychiatry Neurosci 2000; 25:161-166.
(107) Bymaster FP, Calligaro DO, Falcone JF, et al. Radioreceptor binding profile of the atypical antipsychotic Olanzapine. Neuropsychopharmacology 1996; 14:87-96.
(108) Kapur S, Remington G, Jones C, et al. High levels of dopamine d2 receptor occupancy with low-dose haloperidol treatment: a pet study. Am J Psychiatry 1996; 153:948-950.
(109) Tauscher J, Tauscher-Wisniewski S, Kasper S. Treatment of patients with delirium. Am J Psychiatry 2000; 157:1711.
(110) Moots RJ, Al Saffar Z, Hutchinson D, et al. Old drug, new tricks: haloperidol inhibits secretion of proinflammatory cytokines. Ann Rheum Dis 1999; 58:585-587.
(111) Tauscher J, Kufferle B, Asenbaum S, et al. Striatal dopamine-2 receptor occupancy as measured with [123I]iodobenzamide and SPECT predicted the occurrence of EPS in patients treated with atypical antipsychotics and haloperidol. Psychopharmacology (Berl) 2002; 162:42-49.
(112) Tran PV, Dellva MA, Tollefson GD, et al. Extrapyramidal symptoms and tolerability of olanzapine versus haloperidol in the acute treatment of schizophrenia. J Clin Psychiatry 1997; 58:205-211.
(113) Riker RB, Fraser GL, Richen P. Movement disorders associated with withdrawal from high-dose intravenous Haloperidol therapy in delirious icu patients. Chest 1997; 111:1778-1781.
(114) Nielsen J, Bruhn AM. Atypical neuroleptic malignant syndrome caused by olanzapine. Acta Psychiatr Scand 2005; 112:238-240.
(115) Lee PE, Sykora K, Gill SS, et al. Antipsychotic medications and drug-induced movement disorders other than parkinsonism: a population-based cohort study in older adults. J Am Geriatr Soc 2005; 53:1374-1379.
(116) Lindenmayer JP, Czobor P, Volavka J, et al. Changes in glucose and cholesterol levels in patients with schizophrenia treated with typical or atypical antipsychotics. Am J Psychiatry 2003; 160:290-296.
(117) Liperoti R, Pedone C, Lapane KL, et al. Venous thromboembolism among elderly patients treated with atypical and conventional antipsychotic agents. Arch Intern Med 2005; 165:2677-2682.
(118) Miyaji S, Yamamoto K, Hoshino S, et al. Comparison of the risk of adverse events between risperidone and haloperidol in delirium patients. Psychiatry Clin Neurosci 2007; 61:275-282.
(119) Rochon PA, Stukel TA, Sykora K, et al. Atypical antipsychotics and parkinsonism. Arch Intern Med 2005; 165:1882-1888.
(120) Rosencheck R, Perlick D, Bingham S, et al. Effectiveness and cost of olanzapine and haloperidol in the treatment of schizophrenia: a randomized controlled trial. JAMA 2003; 290:2693-2702.
(121) Zornberg GL, Jick H. Antipsychotic drug use and risk of first-time idiopathic venous thromboembolism: a case-control study. Lancet 2000; 356:1219-1223.

(122) Leucht S, Corves C, Arbter D, et al. Second-generation versus first-generation antipsychotic drugs for schizophrenia: a meta-analysis. Lancet 2009; 373:31-41.
(123) Schneider LS, Tariot PN, Dagerman KS, et al. Effectiveness of atypical antipsychotic drugs in patients with Alzheimer's disease. N Engl J Med 2006; 355:1525-1538.
(124) Tyrer P, Kendall T. The spurious advance of antipsychotic drug therapy. Lancet 2009; 373:4-5.
(125) Bishara D, Taylor D. Upcoming agents for the treatment of schizophrenia: mechanism of action, efficacy and tolerability. Drugs 2008; 68:2269-2292.
(126) Schmidt AW, Lebel LA, Howard HR, Jr., et al. Ziprasidone: a novel antipsychotic agent with a unique human receptor binding profile. Eur J Pharmacol 2001; 425:197-201.
(127) Wirshing WC, Wirshing DA, Spellberg B, et al. A comparative review of atypical antipsychotics. Essent Psychopharmacol 1998; 2:431-461.
(128) van Gool WA, van de BD, Eikelenboom P. Systemic infection and delirium: when cytokines and acetylcholine collide. Lancet 2010; 375:773-775.
(129) Glassman AH, Bigger JT. Antipsychotic drugs: prolonged QTc Interval, Torsade de Pointes, and sudded death. Am J Psychiatry 2001; 158:1774-1784.
(130) Reilly JG, Ayis SA, Ferrier IN, et al. QTc-interval abnormalities and psychotropic drug therapy in psychiatric patients. Lancet 2000; 355:1048-1052.
(131) Sharma ND, Rosman HS, Padhi ID, et al. Torsades de Pointes associated with intravenous haloperidol in critically ill patients. Am J Cardiol 1998; 81:238-240.
(132) Harrigan EP, Miceli JJ, Anziano R, et al. A randomized evaluation of the effects of six antipsychotic agents on QTc, in the absence and presence of metabolic inhibition. J Clin Psychopharmacol 2004; 24:62-69.
(133) Straus SM, Bleumink GS, Dieleman JP, et al. Antipsychotics and the risk of sudden cardiac death. Arch Intern Med 2004; 164:1293-1297.
(134) Liperoti R, Gambassi G, Lapane KL, et al. Conventional and atypical antipsychotics and the risk of hospitalization for ventricular arrhythmias or cardiac arrest. Arch Intern Med 2005; 165:696-701.
(135) Roden DM. Drug-induced prolongation of the QT interval. N Engl J Med 2004; 350:1013-1022.
(136) Hatta K, Takahashi T, Nakamura H, et al. The association between intravenous haloperidol and the prolonged QT interval. J Clin Psychopharmacol 2001; 21:257-261.
(137) Levy WO, Robichaux-Keene NR, Nunez C. No significant QTc interval changes with high-dose ziprasidone: a case series. J Psychiatr Pract 2004; 10:227-232.
(138) Miceli JJ, Anziano RJ, Swift RH, et al. IM ziprasidone and IM haloperidol show comparable change in QTc at Cmax. NIMH 43rd Annual NCDEU Meeting . 2002.
(139) Young CC, Lujan E. Intravenous ziprasidone for treatment of delirium in the intensive care unit. Anesthesiology 2004; 101:794-795.
(140) Boulton DW, Kollia G, Mallikaarjun S, et al. Pharmacokinetics and tolerability of intramuscular, oral and intravenous aripiprazole in healthy subjects and in patients with schizophrenia. Clin Pharmacokinet 2008; 47:475-485.
(141) Chan EW, Knott JC, Taylor DM, et al. Intravenous olanzapine--another option for the acutely agitated patient? Emerg Med Australas 2009; 21:241-242.
(142) Miceli JJ, Wilner KD, Swan SK, et al. Pharmacokinetics, safety, and tolerability of intramuscular ziprasidone in healthy volunteers. J Clin Pharmacol 2005; 45:620-630.

(143) Lu JJ, Pallasch EM, Thompson TM, et al. Acute dystonia after intravenous administration of ziprasidone. Clin Toxicol 45, 630. 2007.
(144) Stella VJ, He Q. Cyclodextrins. Toxicol Pathol 2008; 36:30-42.
(145) Martin Del Valle EM. Cyclodextrins and their uses: a review. Process Biochemistry 2004; 39:1033-1046.
(146) Fang J, Baker GB, Silverstone PH, et al. Involvement of CYP3A4 and CYP2D6 in the metabolism of haloperidol. Cell Mol Neurobiol 1997; 17:227-233.
(147) Fang J, McKay G, Song J, et al. In vitro characterization of the metabolism of haloperidol using recombinant cytochrome p450 enzymes and human liver microsomes. Drug Metab Dispos 2001; 29:1638-1643.
(148) Kudo S, Ishizaki T. Pharmacokinetics of haloperidol: an update. Clinical Pharmacokinetics 1999; 37:435-456.
(149) Scordo MG, Spina E. Cytochrome P450 polymorphisms and response to antipsychotic therapy. Pharmacogenomics 2002; 3:201-218.
(150) Lacy CF, Armstrong LL, Goldman MP, et al. Drug Information Handbook. 17 ed. Hudson, Ohio: Lexi-Comp, 2008.
(151) Lipowski ZJ. Delirium in the elderly patient. N Engl J Med 1989; 320:578-582.
(152) Meagher DJ, Hanlon DO, Mahony EO, et al. Relationship between symptoms and motoric subtype of delirium. J Neuropsychiatry Clin Neurosci 2000; 12:51-56.
(153) Peterson JF, Pun BT, Dittus RS, et al. Delirium and its motoric subtypes: a study of 614 critically ill patients. J Am Geriatr Soc 2006; 54:479-484.
(154) Inouye SK. The dilemma of delirium: clinical and research controversies regarding diagnosis and evaluation of delirium in hospitalized elderly medical patients. Am J Med 1994; 97:278-288.
(155) Gustafson Y, Brannstrom B, Norberg A, et al. Underdiagnosis and poor documentation of acute confusional states in elderly hip fracture patients. J Am Geriatr Soc 1991; 39:760-765.
(156) Inouye SK, Foreman MD, Mion LC, et al. Nurses' recognition of delirium and its symptoms: comparison of nurse and researcher ratings. Arch Intern Med 2001; 161:2467-2473.
(157) Sanders AB. Missed delirium in older emergency department patients: a quality-of-care problem. Ann Emerg Med 2002; 39:338-341.
(158) Fitten LJ, Coleman L, Siembieda DW, et al. Assessment of capacity to comply with medication regimens in older patients. J Am Geriatr Soc 1995; 43:361-367.
(159) Nicholas LM, Lindsey BA. Delirium presenting with symptoms of depression. Psychosomatics 1995; 36:471-479.
(160) Truman B, Ely EW. Monitoring delirium in critically ill patients: using the Confusion Assessment Method for the ICU. Crit Care Nurse 2003; 23:25-36.
(161) Lim CJ, Trevino C, Tampi RR. Can olanzapine cause delirium in the elderly? Ann Pharmacother 2006; 40:135-138.
(162) Graham BB, Overdier K, Douglas IS. A Prospective Randomized Study of Haloperidol in Addition to Standard Sedation in Delirious and Intubated Patients: Preliminary Safety Analysis. American Journal of Respiratory & Critical Care Medicine 177, A816. 2008.
(163) Woodward ND, Purdon SE, Meltzer HY, et al. A meta-analysis of cognitive change with haloperidol in clinical trials of atypical antipsychotics: dose effects and comparison to practice effects. Schizophr Res 2007; 89:211-224.

(164) Simpson GM, Angus JW. A rating scale for extrapyramidal side effects. Acta Psychiatr Scand Suppl 1970; 212:11-19.
(165) Pandharipande PP, Sanders RD, Girard TD, et al. Effect of dexmedetomidine versus lorazepam on outcome in patients with sepsis: an a priori-designed analysis of the MENDS randomized controlled trial. Crit Care 2010; 14:R38.
(166) Christie JD, Biester RC, Taichman DB, et al. Formation and validation of a telephone battery to assess cognitive function in acute respiratory distress syndrome survivors. J Crit Care 2006; 21:125-132.
(167) Katz S, Ford AB, Moskowitz RW, et al. Studies of illness in the aged. The index of ADL: a standardized measure of biological and psychological function. JAMA 1963; 185:914-919.
(168) Pfeffer RI, Kurosaki TT, Harrah CH, Jr., et al. Measurement of functional activities in older adults in the community. J Gerontol 1982; 37:323-329.
(169) Marcantonio ER, Michaels M, Resnick NM. Diagnosing delirium by telephone. J Gen Intern Med 1998; 13:621-623.
(170) Badia X, Diaz-Prieto A, Gorriz MT, et al. Using the EuroQol-5D to measure changes in quality of life 12 months after discharge from an intensive care unit. Intensive Care Med 2001; 27:1901-1907.
(171) EuroQol Group. The EuroQoL - a new facility for the measurement of health related quality of life. Health Policy 1990; 16:199-208.
(172) Rabin R, de Charro F. EQ-5D: a measure of health status from the EuroQol Group. Annals of Medicine 2001; 33:337-343.
(173) Kang HK, Li B, Mahan CM, et al. Health of US veterans of 1991 Gulf War: a follow-up survey in 10 years. J Occup Environ Med 2009; 51:401-410.
(174) Eikelenboom P, Hoogendijk WJG. Do delirium and Alzheimer's dementia share specific pathogenetic mechanisms? Dementia Geriatr Cogn Disord 1999; 10:319-324.
(175) Ely EW, Girard TD, Shintani AK, et al. Apolipoprotein E4 polymorphism as a genetic predisposition to delirium in critically ill patients. Crit Care Med 2007; 35:112-117.
(176) Sandyk R, Hurwitz MD. Toxic irreversible encephalopathy induced by lithium carbonate and haloperidol. A report of 2 cases. S Afr Med J 1983; 64:875-876.
(177) Holt R, Siddiqi N, Young J. The ethics of consent in delirium studies. J Psychosom Res 2008; 65:283-287.
(178) Davis N, Pohlman A, Gehlbach B, et al. Improving the process of informed consent in the critically ill. JAMA 2003; 289:1963-1968.
(179) Hedges D, Jeppson K, Whitehead P. Antipsychotic medication and seizures: a review. Drugs Today (Barc) 2003; 39:551-557.
(180) Gazdag G, Barna I, Ivanyi Z, et al. The impact of neuroleptic medication on seizure threshold and duration in electroconvulsive therapy. Ideggyogy Sz 2004; 57:385-390.
(181) Alper K, Schwartz KA, Kolts RL, et al. Seizure incidence in psychopharmacological clinical trials: an analysis of Food and Drug Administration (FDA) summary basis of approval reports. Biol Psychiatry 2007; 62:345-354.
(182) Amann BL, Pogarell O, Mergl R, et al. EEG abnormalities associated with antipsychotics: a comparison of quetiapine, olanzapine, haloperidol and healthy subjects. Hum Psychopharmacol 2003; 18:641-646.
(183) Centorrino F, Price BH, Tuttle M, et al. EEG abnormalities during treatment with typical and atypical antipsychotics. Am J Psychiatry 2002; 159:109-115.

(184) Jorm AF, Jacomb PA. The Informant Questionnaire on Cognitive Decline in the Elderly (IQCODE): socio-demographic correlates, reliability, validity and some norms. Psychol Med 1989; 19:1015-1022.
(185) Jorm AF, Scott R, Cullen JS, et al. Performance of the Informant Questionnaire on Cognitive Decline in the Elderly (IQCODE) as a screening test for dementia. Psychol Med 1991; 21:785-790.
(186) Jorm AF. A short form of the Informant Questionnaire on Cognitive Decline in the Elderly (IQCODE): development and cross-validation. Psychol Med 1994; 24:145-153.
(187) Sessler CN, Gosnell MS, Grap MJ, et al. The Richmond Agitation-Sedation Scale: validity and reliability in adult intensive care unit patients. Am J Respir Crit Care Med 2002; 166:1338-1344.
(188) Ely EW, Truman B, Shintani A, et al. Monitoring sedation status over time in ICU patients: reliability and validity of the Richmond Agitation-Sedation Scale (RASS). JAMA 2003; 289:2983-2991.
(189) Ely EW, Pun BT. The Confusion Assessment Method for the ICU (CAM-ICU) training manual. ICU Delirium and Cognitive Impairment Study Group Website 2005. (Accessed September 20, 2006 at http://www.icudelirium.org/docs/CAM_ICU_training.pdf.)
(190) van Munster BC, Korevaar JC, Zwinderman AH, et al. The association between delirium and the apolipoprotein E epsilon 4 allele: new study results and a meta-analysis. Am J Geriatr Psychiatry 2009; 17:856-862.
(191) van Munster BC, Korevaar JC, de Rooij SE, et al. The association between delirium and the apolipoprotein E epsilon4 allele in the elderly. Psychiatr Genet 2007; 17:261-266.
(192) Schweickert WD, Pohlman MC, Pohlman AS, et al. Early physical and occupational therapy in mechanically ventilated, critically ill patients: a randomised controlled trial. Lancet 2009; 373:1874-1882.
(193) King MS, Render ML, Ely EW, et al. Liberation and Animation: Strategies to Minimize Brain Dysfunction in Critically Ill Patients. Semin Respir Crit Care Med 2010; 31:87-96.
(194) Girard TD, Kress JP, Fuchs BD, et al. "Wake up and breathe" for patients with respiratory failure - Authors' reply. Lancet 2008; 371:1414-1415.
(195) Girard TD. Wake up and breathe flowchart. ICU Delirium and Cognitive Impairment Study Group Website 2008. (Accessed June 10, 2008 at http://www.icudelirium.org/docs/WakeUpAndBreathe.pdf.)
(196) Kress JP, Pohlman AS, O'Connor MF, et al. Daily interruption of sedative infusions in critically ill patients undergoing mechanical ventilation. N Engl J Med 2000; 342:1471-1477.
(197) Ely EW, Baker AM, Dunagan DP, et al. Effect on the duration of mechanical ventilation of identifying patients capable of breathing spontaneously. N Engl J Med 1996; 335:1864-1869.
(198) The Acute Respiratory Distress Syndrome Network. Ventilation with lower tidal volumes as compared with traditional tidal volumes for acute lung injury and the acute respiratory distress syndrome. N Engl J Med 2000; 342:1301-1308.
(199) Lundstrom M, Edlund A, Karlsson S, et al. A multifactorial intervention program reduces the duration of delirium, length of hospitalization, and mortality in delirious patients. J Am Geriatr Soc 2005; 53:622-628.

(200) Vidan MT, Sanchez E, Alonso M, et al. An Intervention Integrated into Daily Clinical Practice Reduces the Incidence of Delirium During Hospitalization in Elderly Patients. J Am Geriatr Soc 2009; 57:2029-2036.
(201) Anderson D. Preventing delirium in older people. Br Med Bull 2005; 73-74:25-34.
(202) Rockwood K. Educational interventions in delirium. Dement Geriatr Cogn Disord 1999; 10:426-429.
(203) Inouye SK, Bogardus ST, Jr., Baker DI, et al. The Hospital Elder Life Program: a model of care to prevent cognitive and functional decline in older hospitalized patients. Hospital Elder Life Program. J Am Geriatr Soc 2000; 48:1697-1706.
(204) Young J, Leentjens AF, George J, et al. Systematic approaches to the prevention and management of patients with delirium. J Psychosom Res 2008; 65:267-272.
(205) Pierre JS. Delirium: a process improvement approach to changing prescribing practices in a community teaching hospital. J Nurs Care Qual 2005; 20:244-250.
(206) Tabet N, Howard R. Non-pharmacological interventions in the prevention of delirium. Age Ageing 2009.
(207) Tabet N, Hudson S, Sweeney V, et al. An educational intervention can prevent delirium on acute medical wards. Age Ageing 2005; 34:152-156.
(208) Inouye SK, Bogardus ST, Jr., Williams CS, et al. The role of adherence on the effectiveness of nonpharmacologic interventions: evidence from the delirium prevention trial. Arch Intern Med 2003; 163:958-964.
(209) Lynch EP, Lazor MA, Gellis JE, et al. The impact of postoperative pain on the development of postoperative delirium. Anesth Analg 1998; 86:781-785.
(210) Young J, Siffleet J, Nikoletti S, et al. Use of a Behavioural Pain Scale to assess pain in ventilated, unconscious and/or sedated patients. Intensive Crit Care Nurs 2006; 22:32-39.
(211) Flinn DR, Diehl KM, Seyfried LS, et al. Prevention, diagnosis, and management of postoperative delirium in older adults. J Am Coll Surg 2009; 209:261-268.
(212) Gelinas C, Fillion L, Puntillo KA, et al. Validation of the critical-care pain observation tool in adult patients. Am J Crit Care 2006; 15:420-427.
(213) Gelinas C, Johnston C. Pain assessment in the critically ill ventilated adult: validation of the critical-care pain observation tool and physiologic indicators. Clin J Pain 2007; 23:497-505.
(214) Riutta A, Ylitalo P, Kaukinen S. Diurnal variation of melatonin and cortisol is maintained in non-septic intensive care patients. Intensive Care Med 2009; 35:1720-1727.
(215) Tembo AC, Parker V. Factors that impact on sleep in intensive care patients. Intensive Crit Care Nurs 2009; 25:314-322.
(216) Salas RE, Gamaldo CE. Adverse effects of sleep deprivation in the ICU. Crit Care Clin 2008; 24:461-4vi.
(217) Weinhouse GL, Schwab RJ. Sleep in the critically ill patient. Sleep 2006; 29:707-716.
(218) Bosma KJ, Ranieri VM. Filtering out the noise: evaluating the impact of noise and sound reduction strategies on sleep quality for ICU patients. Crit Care 2009; 13:151.
(219) Xie H, Kang J, Mills GH. Clinical review: The impact of noise on patients' sleep and the effectiveness of noise reduction strategies in intensive care units. Crit Care 2009; 13:208.
(220) Hopkins RO, Spuhler VJ. Strategies for promoting early activity in critically ill mechanically ventilated patients. AACN Adv Crit Care 2009; 20:277-289.
(221) Skrobik Y. Delirium prevention and treatment. Crit Care Clin 2009; 25:585-91, x.

(222) Nordstrom AL, Farde L, Wiesel FA, et al. Central D2-dopamine receptor occupancy in relation to antipsychotic drug effects: a double-blind PET study of schizophrenic patients. Biol Psychiatry 1993; 33:227-235.
(223) Girard TD, Pandharipande PP, Carson SS, et al. Feasibility, efficacy, and safety of antipsychotics for intensive care unit delirium: the MIND randomized, placebo-controlled trial. Crit Care Med 2010; 38:428-437.
(224) Krantz MJ, Martin J, Stimmel B, et al. QTc Interval Screening in Methadone Treatment. Ann Intern Med 2009; 150:1-9.
(225) Breitbart W, Gibson C, Tremblay A. The delirium experience: delirium recall and delirium-related distress in hospitalized patients with cancer, their spouses/caregivers, and their nurses. Psychosomatics 2002; 43:183-194.
(226) Hui D, Bush SH, Gallo LE, et al. Neuroleptic dose in the management of delirium in patients with advanced cancer. J Pain Symptom Manage 2010; 39:186-196.
(227) Lerch M. Could the Montreal Cognitive Assessment (MoCA) be the new "gold standard" in cognitive evaluation in geriatric patients: a clinical comparison. The Journal of the Alzheimer's Association 2010; 6:S494.
(228) Aggarwall A, Kean E. Comparison of the Folstein Mini Mental State Examination (MMSE) to the Montreal Cognitive Assessment (MoCA) as a Cognitive Screening Tool in an Inpatient Rehabilitation Setting. Neuroscience & Medicine 2010; 1:39-42.
(229) Van Eijk MM, van Marum RJ, Klijn IA, et al. Comparison of delirium assessment tools in a mixed intensive care unit. Crit Care Med 2009; 37:1881-1885.
(230) Guenther U, Popp J, Koecher L, et al. Validity and reliability of the CAM-ICU Flowsheet to diagnose delirium in surgical ICU patients. J Crit Care 2010; 25:144-151.
(231) Fraser GL, Prato BS, Riker RR, et al. Frequency, severity, and treatment of agitation in young versus elderly patients in the ICU. Pharmacotherapy 2000; 20:75-82.
(232) Morrison RS, Magaziner J, Gilbert M, et al. Relationship between pain and opioid analgesics on the development of delirium following hip fracture. J Gerontol A Biol Sci Med Sci 2003; 58:76-81.
(233) Ouimet S, Kavanagh BP, Gottfried SB, et al. Incidence, risk factors and consequences of ICU delirium. Intensive Care Med 2007; 33:66-73.
(234) Reade MC, O'Sullivan K, Bates S, et al. Dexmedetomidine vs. haloperidol in delirious, agitated, intubated patients: a randomised open-label trial. Crit Care 2009; 13:R75.
(235) Carson SS, Kress JP, Rodgers JE, et al. A randomized trial of intermittent lorazepam versus propofol with daily interruption in mechanically ventilated patients. Crit Care Med 2006; 34:1326-1332.
(236) Roth DL, Burgio LD, Gitlin LN, et al. Psychometric analysis of the Revised Memory and Behavior Problems Checklist: factor structure of occurrence and reaction ratings. Psychol Aging 2003; 18:906-915.
(237) Teri L, Truax P, Logsdon R, et al. Assessment of behavioral problems in dementia: the revised memory and behavior problems checklist. Psychol Aging 1992; 7:622-631.
(238) Bedard M, Molloy DW, Squire L, et al. The Zarit Burden Interview: a new short version and screening version. Gerontologist 2001; 41:652-657.
(239) Charlson ME, Pompei P, Ales KL, et al. A new method of classifying prognostic comorbidity in longitudinal studies: development and validation. J Chronic Dis 1987; 40:373-383.

(240) Saunders JB, Aasland OG, Babor TF, et al. Development of the Alcohol Use Disorders Identification Test (AUDIT): WHO Collaborative Project on Early Detection of Persons with Harmful Alcohol Consumption--II. Addiction 1993; 88:791-804.
(241) Knaus WA, Draper EA, Wagner DP, et al. APACHE II: a severity of disease classification system. Crit Care Med 1985; 13:818-829.
(242) Vincent JL, Moreno R, Takala J, et al. The SOFA (Sepsis-related Organ Failure Assessment) score to describe organ dysfunction/failure. Intensive Care Med 1996; 22:707-710.
(243) Vincent JL, Mendonca Ad, Cantraine F, et al. Use of the SOFA score to assess the incidence of organ dysfunction/failure in intensive care units: Results of a multicenter, prospective study. Crit Care Med 1998; 26:1793-1800.
(244) Thompson BT, Schoenfeld D. Usual care as the control group in clinical trials of nonpharmacologic interventions. Proc Am Thorac Soc 2007; 4:577-582.
(245) Garrouste Org, Timsit JF, Soufir L, et al. Impact of adverse events on outcomes in intensive care unit patients. Crit Care Med 2008; 36:2041-2047.
(246) Senn S. Statistical issues in drug development. Statistical Issues in Drug Development. West Sussix, England: John Wiley and Sons, Ltd., 1997: 98-99.
(247) Hernandez AV, Steyerberg EW, Habbema JD. Covariate adjustment in randomized controlled trials with dichotomous outcomes increases statistical power and reduces sample size requirements. J Clin Epidemiol 2004; 57:454-460.
(248) Cox DR. Regression models and life-tables. J R Stat Soc Ser B 1972; 34:187-220.
(249) Concato J, Peduzzi P, Holford TR, et al. Importance of events per independent variable in proportional hazards analysis. I. Background, goals, and general strategy. J Clin Epidemiol 1995; 48:1495-1501.
(250) Peduzzi P, Concato J, Kemper E, et al. A simulation study of the number of events per variable in logistic regression analysis. J Clin Epidemiol 1996; 49:1373-1379.
(251) Rothman KJ. No adjustments are needed for multiple comparisons. Epidemiology 1990; 1:43-46.
(252) Perneger TV. What's wrong with Bonferroni adjustments. BMJ 1998; 316:1236-1238.
(253) Cook RJ, Farewell V. Multiplicity considerations in the design and analysis of clinical trials. J Roy Statist Soc A 1996; 159:93-110.
(254) O'Brien PC, Fleming TR. A multiple testing procedure for clinical trials. Biometrics 1979; 35:549-556.
(255) Roderick J, Little A, Rubin DB. Statistical Analysis with Missing Data. Wiley, 1987.
(256) Cremer OL, Moons KG, van Dijk GW, et al. Prognosis following severe head injury: Development and validation of a model for prediction of death, disability, and functional recovery. J Trauma 2006; 61:1484-1491.
