# Supplementary material for: Antipsychotics and the QTc Interval During Delirium in the Intensive Care Unit: A Secondary Analysis of a Randomized Clinical Trial
Source: JAMA Netw Open. 2024 Jan 22;7(1):e2352034. doi: 10.1001/jamanetworkopen.2023.52034 (PMC10804270; doi:10.1001/jamanetworkopen.2023.52034)
Supplement: Supplement 2. — Statistical Analysis Plan [file jamanetwopen-e2352034-s002.pdf]

1. Administrative Information

Title	The Modifying the Impact of ICU-Associated Neurological Dysfunction-USA (MIND-USA) Study
Trial Registration	https://clinicaltrials.gov/ct2/show/NCT01211522
Principal Investigators	E. Wesley Ely, MD<br>Timothy Girard, MD
Biostatistics	Rameela Chandrasekhar, PhD<br>Jennifer Thompson, MPH
Date	May 7, 2018
SAP Version	1.0

2. Introduction

This serves as the formal Statistical Analysis Plan (SAP) for The Modifying the Impact of ICU-Associated Neurological Dysfunction-USA (MIND-USA) Study, finalized before unblinding of the treatment groups. The trial is registered at clinicaltrials.gov/ct2/show/NCT01211522. This SAP is written based on guidelines in Gamble et al, JAMA 2017.

2.1 Background and Rationale

Antipsychotics are the first-line pharmacological agents recommended to treat delirium, and over the past 30 years they have gained widespread use in hospitalized patients globally prior to adequate testing of efficacy and safety for this indication. Haloperidol, the most commonly chosen antipsychotic, is used by over 80% of ICU doctors for delirium, while atypical antipsychotics are prescribed by 40%. Antipsychotics safety concerns include lethal cardiac arrhythmias, extrapyramidal symptoms, and the highly publicized increased mortality associated with their use in non-ICU geriatric populations. The long-term objective of the study is to define the role of antipsychotics in the management of delirium in vulnerable critically ill patients, specifically as they relate to short- and long-term clinical outcomes, including days alive without acute brain dysfunction (referred to as delirium/coma-free days or DCFDs) over a 14-day period; 30-day, 90-day, and 1-year survival; ICU length of stay; incidence, severity, and/or duration of long-term neuropsychological dysfunction; and quality of life at 90-day and 1-year.

2.2 Objectives

The MIND-USA study is a multicenter, double-blind, randomized, placebo-controlled trial investigating the effects of intravenously (IV) administered typical and atypical antipsychotics (haloperidol and ziprasidone) on delirium in critically ill patients. The study evaluates the following aims:

- **Aim 1:** To determine whether haloperidol or ziprasidone administered to delirious medical/surgical ICU patients will increase days alive without delirium and coma (measured as delirium/coma-free days [DCFDs]) over a 14-day study period compared with placebo.
- **Aim 2:** To determine whether haloperidol or ziprasidone will improve 30-day, 90-day, and 1-year survival compared with placebo.
- **Aim 3:** To determine whether haloperidol or ziprasidone will reduce ICU length of stay (LOS) (i.e., time to ICU discharge) compared with placebo.
- **Aim 4:** To determine whether haloperidol or ziprasidone will improve long-term neuropsychological outcomes, functional independence, quality of life, and post-traumatic stress disorder symptoms at 3-month and 1-year follow-up compared with placebo.

3. Study Methods

3.1 Trial Design

This is a multicenter, double-blind, randomized, placebo-controlled trial. The two treatment arms comprise a typical antipsychotic (haloperidol) and an atypical antipsychotic (ziprasidone). Patients were consented and enrolled upon meeting inclusion and exclusion criteria; patients were then randomized to receive study drug if and when they became delirious, per research coordinator CAM and RASS assessment.

3.2 Randomization

Randomization to haloperidol, ziprasidone, or placebo was conducted in a 1:1:1 ratio using a computer-generated permuted-block randomization scheme stratified by study center and age (<70 years vs. ≥70 years). The randomization scheme was created by a biostatistician external to the study and distributed directly to the investigational pharmacy at each study site. Unblinding of the treatment groups (and subsequent data lock) will be performed after data cleaning and will be documented. Any unlock of the database will be performed only to correct serious data entry errors and will be documented in a detailed manner.

3.3 Power and Sample Size *(as was written in the grant)*

Power Analyses and Sample Size Calculations for Delirium (Aim 1). Based on data collected during our BRAIN-ICU Study, we anticipate that patients in the control (placebo) group in MIND-USA will have a mean ± SD of 6.8±5.2 delirium/coma-free days during the 14-day Treatment Period. At a 2-sided significance level of 2.50%, after Bonferroni adjustment for 2 pairwise comparisons (each active treatment will be compared to placebo), a trial of 187 patients per treatment group (total N=561) will have 92% analytical power to detect a 2-day improvement in delirium/coma-free days. Importantly, this sample size will also provide 80% power to detect a 2.5-day improvement in DCFDs within 4 subgroups expected to be ~45% (N=252) of the study population: severe sepsis, age ≥65 years, high illness severity (APACHE II ≥25), and medical vs. surgical patients. We will also conduct a hypothesis-generating subgroup analysis for patients enrolled with preexisting cognitive impairment (~25% of study population, N=140).

Power Analyses for Mortality and ICU LOS (Aims 2 and 3). Assuming 40% one-year mortality in the control (placebo) group, this study will have 80% power to detect a 13% absolute difference in one-year mortality at a 2-sided significance level of 1.67% (if control mortality is 50%, study has 80% power to detect 14% difference). For ICU length of stay (LOS), this study will have 80% power to detect a 2-day (24%) difference at a 2-sided significance level of 1.67% assuming an 8.2±7.6 day ICU LOS in controls.

Timing of Final Analysis

In-Hospital Database Cleaning & Lock Procedures

MIND-USA uses the REDCap electronic data capture platform for data collection. Upon completion of the in-hospital portion of the MIND-USA study, the following procedures will be followed and documented within the Database Cleaning & Lock SOP:

1. VCC will work with site coordinators to address all data issues revealed by ongoing data cleaning. This process will continue until all issues have been addressed.
2. Upon completion of in-hospital data cleaning, the REDCap database **MIND-USA Study: Exclusion Log** will be locked in the following way:
   a. Initially all users with current "view and edit" user privileges will be moved to "read only" user privileges.
   b. After the window closes for sites to export their data the database will be permanently moved to inactive status (meaning that no data can be changed).
3. Upon completion of in-hospital data cleaning, the REDCap database **MIND-USA Study: In-Hospital Database** will be locked in the following way:
   a. Initially all study site personnel will be restricted to "read-only" user access for the entire database. VCC Project Managers and the Follow-Up Team will be restricted to read-only access for all fields except those needed for patient contact, reconsenting, DNA permissions, notes to file and event reporting, and tracking dates of death and study withdrawal. All fields not needed by these teams will be restricted to read only by use of the @readonly action tags. The follow-up team will continue to be blinded (via restricted access) to all information about the hospital course, as has been the case throughout the study.

	**VCC Project Managers**	**Follow-Up Team**
Dates Tracking Form - all variables made read-only (using action tag @readonly) to all users except variables pertaining to consenting, death and study withdrawal	View and Edit	View and Edit
Contact Form - all fields	Read Only	View and Edit
NTF - all fields	View and Edit	View and Edit
Adverse Events - all fields	View and Edit	View and Edit
DNA Log - all fields	View and Edit	No Access
All other forms/fields	Read Only	No Access

b. During the remaining Follow-Up period, the sites will be given a window for downloading their data for local storage.
c. Once 12-month follow-up is completed, we will conduct final data cleans on the updated information and then permanently move the entire database to inactive status, meaning that no data can be changed unless serious errors are noted.

A log of all steps in this process will be maintained in the Database Cleaning & Lock SOP.

4. Statistical Principles

Statistical analysis will be conducted in accordance to the plan outlined in this SAP. Statistical analysis will abide by these general statistical principles below.

Confidence Intervals and P-Values

Level of statistical significance will be set at 5% (with multiple comparisons adjustments for the primary outcome, as noted below). All tests will be two-sided. 95% confidence intervals will be reported along with all effect estimates. Presentation of results will emphasize clinical significance, effect sizes and confidence intervals, over statistical significance.

Modeling Principles

Whenever possible (based on variable distribution), we will not assume linear associations between covariates and outcomes; rather, nonlinear associations between continuous variables and outcomes will be permitted by inclusion of restricted cubic splines with 3 knots. To account for correlation among patients within a given site, we will adjust standard errors using Huber-White sandwich estimation.

Multiple Comparisons

For the primary outcome (DCFDs), multiple comparisons will be conducted at a 2.5% level if the overall test of association is significant at a 5% level. Regarding the analyses of all *a priori*-defined secondary outcomes described herein, no adjustments will be made for multiple comparisons, in keeping with authoritative recommendations on this topic and standard practice when analyzing multiple, prospectively defined outcomes in a clinical trial. As described in our proposal, each comparison made will be hypothesis-driven and based upon biological plausibility rather than hypothesis-generating in nature.

Missing Data

Individual Variables

Missing data for individual variables will be imputed using clinical imputation rules when appropriate; details on these rules are noted in the Definitions and Derived Variables section. This section also describes the imputation process for summary variables for mental status (eg, days alive and free of delirium and coma), which may involve *partially* missing data.

Missingness when Modeling

In adjusted analyses, model-based multiple imputation strategies will be used (using R's mice library) if >5% of any covariates are missing. In all cases, decisions and processes will be documented both in data management and analysis code and in statistical reports.

Adherence to the Intervention and Protocol Noncompliance

Definition & assessment of adherence to the intervention

All analysis will be conducted based on the intention to treat principle. Subjects will be considered to have the treatment they were randomized to regardless of withdrawal from treatment or extent of exposure.

Presentation of adherence to intervention

We will describe, within each treatment group, the following:

- Patient level:
  - How many randomized patients received >=1 dose of study treatment
  - Days each randomized patient received study treatment
  - Doses of study treatment each randomized patient received, along with average daily dose
  - Whether study treatment was ever held or permanently discontinued, and reasons for hold/discontinuation
- Dose level:
  - Proportion of opportunities to receive study treatment which were given, temporarily held, or permanently discontinued
  - Amount (mL) of each dose given
  - Whether dose amount was maintained, increased, or decreased
  - Reasons for hold or discontinuation

Definition and description of protocol noncompliance

Any noncompliance that increased safety risk to the patient was considered protocol noncompliance. These events will be captured for a variety of causes considered related to patient safety and will be described in the final study report, broken down according to a simple categorization scheme followed prospectively during the conduct of the MIND-USA investigation.

Analysis Populations

All analysis will be conducted on the randomized, intent-to-treat population. We will also compare demographic and admission characteristics of patients who were enrolled but not randomized to those who were randomized.

Analysis for all **in-hospital** outcomes will include all randomized patients in an intent-to-treat manner, with the following exceptions:

1. Liberation from mechanical ventilation will include only patients who were on mechanical ventilation (invasive or noninvasive) at or within 24 hours following the time of randomization.
2. ICU readmission will include only patients who survived and remained in the study with access to medical data throughout their first ICU admission.

Analysis for **long-term** outcomes will include all patients who survive and remain in the study at the specified time point (3 or 12 months after randomization), and who have data for the outcome in question. We will describe the general cohort of patients who are included in any long-term outcome models at each time point.

5. Trial Population

The study inclusion and exclusion criteria can be obtained from clinicaltrials.gov. Patient flow information as recommended by CONSORT guidelines will be presented for both enrolled and randomized patients, including screening, exclusions, refusal of consent, enrollment, randomization, withdrawals, and hospital discharge status. We will describe, by treatment group and overall, baseline characteristics of randomized patients including demographics (age, race, ethnicity, gender, insurance status, education, BMI); baseline clinical status (comorbidities, home antipsychotic use, frailty, disability, cognition); and ICU admission characteristics (admission reason, severity of illness).

6. Statistical Analysis

Outcome Definitions

Primary outcome (PO)

The primary outcome is delirium/coma-free days (DCFDs) over a 14-day study period, defined as the number of days during the 14-day intervention period (beginning on the day of randomization) that the patient was alive and experienced neither delirium nor coma.

Secondary outcomes (SO)

Time frames, as noted in parentheses, all begin on the day of randomization to study treatment, with the exception of ICU readmission.

SO1. Survival
  - Description: Time to death
  - Time frame: 30 days, 90 days, 1 year

SO2. Delirium duration
  - Description: Duration of delirium during the intervention period
  - Time frame: 14 days

SO3. Time to final ICU discharge
  - Description: Days from randomization to final, successful ICU discharge, where "successful" indicates that discharge was followed by at least 48 hours alive. "ICU discharge" is represented by readiness for ICU discharge indicated by a physician order for transfer to a lower level of care even if a bed availability problems prevent actual discharge from the ICU.
  - Time frame: 90 days

SO4. Time to hospital discharge
  - Description: Days from randomization to successful hospital discharge, where "successful" indicates that discharge was followed by at least 48 hours alive.
  - Time frame: 90 days

SO5. Time to liberation from mechanical ventilation
  - Description: Days from randomization to successful liberation from mechanical ventilation, where "successful" indicates that liberation was followed by at least 48 hours alive and without reinitiation of invasive or noninvasive ventilation.
  - Time frame: 30 days

SO6. Time to ICU readmission
  - Description: Days from first ICU discharge to next ICU readmission within the index hospitalization.
  - Time frame: 90 days after first ICU discharge

SO7. Hospital readmission
- Description: Readmission to the hospital after index hospital discharge determined by self-report during follow-up interviews.
- Time frame: 365 days

SO8. Neuropsychological dysfunction
- Description: Assessed using Telephone Interview for Cognitive Status (TICS), Digit Span and Similarities from the WAIS-III, Confusion Assessment Method (CAM) Telephone version, Paragraph Recall (both immediate and delayed portions) from the WMS-III, Controlled Oral Word Association Test (COWA), and the Hayling Test. These assessments will be scored in standard fashion and will allow us to characterize cognitive impairment across patients.
- Time frame: 3 and 12 months post-randomization

SO9. Quality of life
- Description: Assessed using the Katz ADL, Employment Questionnaire, Functional Activities Questionnaire (FAQ), EQ-5D-3L and a Healthcare Utilization Survey.
- Time frame: 3 and 12 months post-randomization

SO10. Post-traumatic stress disorder
- Description: Assessed using the PTSD Checklist (PCL-S, event specific version) with respondents instructed to answer questions in reference to the ICU experience.
- Time frame: 3 and 12 months post-randomization

SO11. Torsades de pointes
- Description: Incidence of tachyarrhythmias determined to be torsades de pointes after review by the site primary investigator, DSMB, and coordinating center.
- Time frame: 14 days plus 4-day post-study drug period (if longer than 14 days)

SO12. Extrapyramidal symptoms
- Description: Occurrence and severity of extrapyramidal symptoms as measured by a modified Simpson-Angus Scale, Akathisia Visual Analogue Scale, and a standardized definition of dystonia
- Time frame: 14 days plus 4-day post-study drug period (if longer than 14 days)

SO13. Neuroleptic malignant syndrome
- Description: Incidence of neuroleptic malignant syndrome identified by the clinical team and confirmed by the site primary investigator and coordinating center
- Time frame: 14 days plus 4-day post-study drug period (if longer than 14 days)

7. Analysis Methods

All in-hospital outcomes will be analyzed using both univariate methods and multivariable regression, adjusting for covariates noted below. Though patient characteristics should theoretically be balanced between treatment groups due to randomization, adjustment increases our power and precision. Though we will perform both types of analyses for each outcome, **adjusted analyses will be considered the primary analysis.**

Unadjusted Analyses

In-Hospital Continuous Outcomes

We will analyze continuous outcomes (delirium/coma-free days [**PO**]; delirium duration [total, **SO2**; hyperactive and hypoactive delirium separately; coma duration; mean CV SOFA and lowest mean arterial pressure]) using the Kruskal-Wallis test. These outcomes are typically not normally distributed; therefore, the assumptions for a parametric ANOVA would be violated, and results would be unreliable. The nonparametric Kruskal-Wallis test does not assume that the outcome has a normal distribution, and thus provides more power and reliability in the case of a non-normal distribution.

If the unadjusted test for the primary outcome (DCFDs) only is statistically significant, we will use Dunn's test for pairwise differences between treatment groups, using a Bonferroni correction for multiple comparisons at a 2.5% alpha level.

Time to Event Outcomes

We will describe and test for differences in time to death (**SO1**) using Kaplan-Meier curves and the log-rank test, respectively. ICU and hospital discharge, liberation from mechanical ventilation, and ICU readmission all have a competing risk of death (**SO3**, **SO4**, **SO5**, and **SO6**, respectively). (For ICU readmission and liberation from mechanical ventilation, hospital discharge without experiencing the outcome will be treated as an additional competing risk.) Therefore, we will describe the cumulative incidences of both the outcome of interest and each competing risk, along with a modified chi-squared test for the difference between groups in the subdistribution of interest (Gray 1988). Patients who withdrew in the hospital with no discharge or death information available are censored at the time of withdrawal; we censor at x.01 days anyone who has experienced neither death nor the outcome of interest by x days (where x is the end of the time frame specified above for each outcome). We will detail how many and when patients were censored for each analysis.

"Time 0" will be the time of randomization for each of these outcomes, with the exception of ICU readmission; for this outcome, "time 0" will be the time of the first ICU discharge, and only patients who survived their first ICU admission will be included.

Long-Term Outcomes

We will use multivariable regression to analyze the relationship between treatment group and scores measuring long-term neuropsychological, quality of life, and PTSD outcomes as outlined in the Outcomes section above. Depending on the distribution of each outcome, we will use linear regression, negative binomial regression, or proportional odds logistic regression, as appropriate. These models will not include covariates other than treatment.

For each outcome, we will have separate models for 3- and 12-month scores. At each time point, we include all patients with available outcome data, using inverse probability weighting to account for patient death and attrition. To decrease reliance on a few highly influential observations, we will truncate weights at the top 1 percentile.

As a sensitivity analysis, we will also define long-term outcomes using the unadjusted composite endpoint approach (reference: Lachin) to deal with outcomes truncated due to death or loss to follow up. The composite endpoint will be defined as:

- If the patient dies prior to assessment: days between randomization and death
- If the patient survives and is successfully assessed: days between randomization and planned assessment (constant) + assessment score

For example, if we are analyzing a 3-month outcome, the constant would be 90. Differences between the composite end point distribution amongst groups will be assessed using the Kruskal-Wallis test.

Adjusted Analyses

We will adjust all coefficient variances using Huber-White sandwich estimation, clustered by study site. This will help account for unmeasured variability and correlation among patients within a given site.

In-Hospital Continuous Outcomes

We will use proportional odds logistic regression for continuous outcomes (delirium/coma-free days; delirium duration [any, hyperactive, and hypoactive]; coma duration; mean CV SOFA and lowest mean arterial pressure); this method assumes an ordinal outcome but does not assume that it follows a specific statistical distribution.

If the overall test for treatment vs our primary outcome (DCFDs) is significant at the 5% level, we will report individual treatment tests vs placebo at the 2.5% level.

Time to Event Outcomes

We will use Cox proportional hazards regression for mortality. For all other time to event outcomes (ICU and hospital discharge, ICU readmission, and liberation from mechanical ventilation), we use Fine-Gray competing risks regression, treating death as our competing risk. (For ICU readmission and liberation from mechanical ventilation, hospital discharge without experiencing the outcome will be treated as an additional competing risk.) Patients who withdrew in the hospital with no discharge or death information available will be censored at the time of withdrawal; we will censor at x.01 days anyone who has experienced neither death nor the outcome of interest by x days, where x is the end of the time frame specified above for each outcome. Details on censoring will be provided with each analysis.

“Time 0” will be the time of randomization for each of these outcomes, with the exception of ICU readmission; for this outcome, “time 0” will be the time of the first ICU discharge, and only patients who survived their first ICU admission will be included.

Long-Term Outcomes

We will use the same modeling techniques as described in “Unadjusted Analyses” above; however, here, we will adjust for the covariates listed below.

Model Assumptions

Model assumptions will be evaluated graphically. Proportional odds assumptions will be checked using logistic regression with multiple cutoffs for proportional odds assumption (see Harrell’s *Regression Modeling Strategies*, section 13.3.3) and Schoenfeld residuals will be used for proportional hazards. If linear regression is used for long-term outcomes, we check residual vs fitted plots and quantile-quantile plots to ensure assumptions are met. For negative binomial models, we will check goodness-of-fit measures.

Covariates

Covariates for all multivariable regression models include (all demographic or ICU admission characteristics):

- Age at study consent
- Preexisting CI, via the IQCODE (performed via patient or surrogate questionnaire)
- Preexisting frailty, via the CSHA Clinical Frailty Score
- Preexisting comorbidities, via the Charlson Comorbidities Index
- SOFA on the day of randomization, excluding the CNS component
- Level of arousal at randomization, via the RASS closest to the time of randomization

Prior to modeling, we perform redundancy analyses to ensure that no covariates completely explain any of the others (resulting in multicollinearity).

Exploratory Analysis

In addition to the primary and secondary outcomes listed on clinicaltrials.gov, the following additional analyses will be used to inform specific decisions on missing data and modeling, to elucidate findings from primary outcomes, and more fully describe the course of the intervention:

- Exploration and description of outcome and covariate missingness
- Distribution of all continuous covariates, to determine ability to use restricted cubic splines and knot placement
- Coma duration as an additional outcome, to aid in elucidating relationship between treatment and primary outcome of DCFDs; will be analyzed in the same manner as delirium duration (Kruskal-Wallis for unadjusted analysis, and proportional odds logistic regression for adjusted analysis, with the same covariates)
- Durations of a) hypoactive and b) hyperactive delirium as additional outcomes, to describe any relationship between treatment and specific types of delirium. These will be defined as follows:
    - Hypoactive: CAM positive and RASS -3, -2, -1, or 0
    - Hyperactive: CAM positive and RASS +1, +2, +3, or +4
- Daily compliance on the first five elements (A-E) of the ICU Liberation ABCDEF Bundle during the intervention period (number and % of eligible days compliant; descriptive statistics only).
- Description of incidents of Torsades de pointes, to accompany other secondary outcomes related to safety.
- Severity of Shock
    - Description:
        - Mean CV SOFA per day
        - Mean lowest MAP per day
    - Time Frame: 14 days plus 4-day post-study drug period (if longer than 14 days)

Subgroup/Effect Modification Analysis

We will assess whether patient characteristics modify the effects of the interventions on outcomes to identify a subcategory of patients who may benefit more than others. For these analyses, we will develop separate multivariable regression models that include interaction terms between study group and the following clinical characteristics:

- Age at consent (continuous)
- Presence of severe sepsis at ICU admission (yes/no)
- Preexisting cognitive impairment (measured by the IQCODE; continuous covariate)
- Medical vs surgical patients
  Surgical patients are those who have a recorded primary ICU admission reason involving surgery; had emergency or elective surgery between hospital admission and ICU admission; and/or went to the operating room between ICU admission and study enrollment. All other patients will be considered medical patients.

8. Definitions and Derived Variables

Severity of Illness

Due to the nature of clinical data collection, we have some missing values for APACHE II and SOFA components despite our coordinators' best efforts. We handled these missing values in the following ways:

APACHE II (ICU admission only)

- Oxygenation: If no arterial blood gas was done, we converted the lowest O2 saturation to PaO2 per the EPIC II conversions and assigned points based on PaO2 alone. O2 saturations below the lowest level included in the conversion table were assigned the lowest PaO2; O2 saturations of 100 were assigned the highest PaO2.
- pH: If no arterial blood gas was done, we used the serum HCO3 conversions noted in the original reference.
- Glasgow Coma Score: If no GCS was available, we assigned points for the APACHE using the lowest RASS on the day of ICU admission using Vasilevskis et al's point values for the SOFA.
- All other components: If no values were available on a given day, we looked for a value on the closest day within the three full days after ICU admission. If none was available, we assumed that no measurement implied no clinical reason to suspect dysfunction, and assigned a normal value (0 points).

SOFA (ICU admission + daily throughout intervention period)

- Substitutions for specific components:
    - Respiratory: If P/F ratio was not available, we used the lowest S/F ratio, per Pandharipande et al.
    - Central nervous system: If no GCS was available, we used the lowest RASS available that day, per Vasilevskis et al, method C.
- Missing data at ICU admission: Using only data from ICU admission, there are 28 consented patients and 7 randomized patients missing at least one SOFA component score. For these patients' missing components, we imputed the next available value within the following two calendar days. If none was available, we assumed a normal value (0 points). (This could happen either because there was no clinical reason to order labs, or because the patient was not consented within three days of ICU admission and thus no study data was collected in that period.)
- Missing data during the intervention period:
    - We were unable to calculate SOFA scores using raw data on 3 patient-days during the study period (randomization + 13 days). *(Missingness is much higher after the official intervention/post-intervention periods, when patients were no longer being actively followed for daily data collection.)*

    - If the data required to calculate a given component was unavailable, we imputed the closest non-missing component score before *or* after the missing day, up to two full days away (missing day +/-2 days). If data was available X days both before *and* after the missing day, we prioritized past over future values.
    - If, after this imputation, values are still missing, we assume that no available data indicates no clinical reason to suspect organ dysfunction and therefore impute a normal value for that component. Again, this applies to 3 patient-days during the study period.

Medications

- Benzodiazepines include midazolam, lorazepam, and/or diazepam. Doses are expressed in midazolam equivalents.
- Opioids include fentanyl, morphine, and/or hydromorphone. Doses are expressed in fentanyl equivalents.
- Antipsychotics include open-label haloperidol, open-label ziprasidone, quetiapine, aripiprazole, olanzapine (including in combination with fluoxetine), and/or risperidone. Doses are expressed in haloperidol equivalents.
- All conversion formulas can be found in this spreadsheet.

Mechanical Ventilation

During the course of the study, patients could be on invasive mechanical ventilation (MV), noninvasive positive pressure support (NIPPV), both, or neither. For our purposes, “time on MV” describes the number of days each patient was on *either* type of MV beginning at the time of randomization, including time between discontinuation of MV and reinitiation or death, if that time is less than 48 hours. (In other words, if a patient was extubated at 12pm and died at 3pm, those final three hours are included in the total time on MV.)

“Liberation from mechanical ventilation” indicates the first discontinuation of either type of MV which was followed by at least 48 hours alive without reinitiation of MV.

Delirium/Coma-Free Days

This primary outcome variable is calculated over the 14 days including and immediately following randomization. It is defined as days alive and without brain dysfunction.

Mental Status (Delirium and Coma)

Determining Mental Status Using CAM and RASS

We determined mental status for a given *assessment* using the following criteria:

1. Comatose: RASS -4 or -5, or RASS missing and CAM Unable to Assess
2. Delirious: RASS missing or >= -3, and CAM Positive

3. Normal: RASS missing or >= -3, and CAM Negative

Patients could have multiple assessments on a given study day. On a given *day*, a patient was considered delirious if any assessment was considered delirious; comatose if no assessments met criteria for delirium and at least one was considered comatose; and normal if no assessments met criteria for delirium or coma, and at least one was considered normal.

Handling Missing Data

Prior to unblinding, the data shows that among all consented patients, 401 (4%) in-hospital patient-days have insufficient information to determine mental status (due to missing data or study withdrawal). Among randomized patients during the intervention period, 251 (4%) of patient-days have missing mental status, again due either to missing data or study withdrawal. Mental status can change quickly; therefore, simple imputation methods like last observation carried forward could be inaccurate. Since we have strong covariate data, we performed single imputation using polytomous logistic regression, including the following variables as covariates in the imputation.

- Baseline: age at consent; gender; BMI; education; level of proficiency in English; insurance; home antipsychotic use; Charlson comorbidities index; CSHA Clinical Frailty Score; APACHE II Acute Physiology Score
- Daily:
    - Medications (clonazepam, dexmedetomidine, propofol, remifentanil, antipsychotics, benzodiazepines, opioids [IV and PO], antibiotics, anxiolytics, statins)
    - Variables indicating severity of illness (CV SOFA, creatinine, urine output, platelets, lowest recorded RASS, Glasgow Coma Scale, P/F ratio, S/F ratio, bilirubin)
    - Any mental status data available the day of, the day before, and the day after the missing day

All summary variables (delirium/coma-free days, delirium duration, and coma duration) are presented using imputed mental status.

9. Software Details

R version 3.4.4 (2018-03-15) or above will be used for all analyses. Versions of specific packages used for analysis will be noted in the analysis report. The checkpoint package will be used to preserve R package versions throughout the manuscript submission and review process.

10. References

1. Gamble C, Krishan A, Stocken D, et al. Guidelines for the content of statistical analysis plans in clinical trials. JAMA. doi: 10.1001/jama.2017.18556
2. R. Gray. A class of k-sample tests for comparing the cumulative incidence of a competing risk. Ann Stat, 16:1141-1154, 1988.
3. Fine, J., & Gray, R. (1999). A Proportional Hazards Model for the Subdistribution of a Competing Risk. *Journal of the American Statistical Association, 94*(446), 496-509. doi:10.2307/2670170
4. Harrell. Regression Modeling Strategies with Applications to Linear Models, Logistic Regression, and Survival Analysis 2001, Springer-Verlag. link
5. Pandharipande P, Shintani AK, Hagerman HE, St Jacques PJ, Rice TW, Sanders NW, Ware LB, Bernard GR, Ely EW. Derivation and validation of Spo2/FiO2 ratio to impute for PaO2/FiO2 ratio in the respiratory component of the Sequential Organ Failure Assessment Score. Crit Care Med, 2009 Apr; 37(4): 1317-21. doi: 10.1097/CCM.0b013e31819cefa9
6. Vasilevskis EE, Pandharipande PP, Graves AJ, Shintani A, Tsuruta R, Ely EW, Girard TD. Validity of a Modified Sequential Organ Failure Assessment Score Using the Richmond Agitation-Sedation Scale. Crit Care Med 2016 Jan; 44(1): 138-146. doi: 10.1097/CCM.0000000000001375
7. Lachin JM. Worst-rank score analysis with informatively missing observations in clinical trials. Control Clin Trials 1999 Oct; 20(5):408-22. PMID: 10503801.
