# Supplementary material for: Antipsychotics and the QTc Interval During Delirium in the Intensive Care Unit: A Secondary Analysis of a Randomized Clinical Trial
Source: JAMA Netw Open. 2024 Jan 22;7(1):e2352034. doi: 10.1001/jamanetworkopen.2023.52034 (PMC10804270; doi:10.1001/jamanetworkopen.2023.52034)
Supplement: Supplement 3. — eTable 1. Daily Average Predose QTc Interval Across All Study Days eTable 2. The Effect of Pharmacologic Medication With QTc Interval Changes by Sex eFigure 1. Association Between ECG and Telemetry QTc Interval During Study Drug Held for QTc Prolongation eFigure 2. The Distribution of Initial Postdose QTc Interval eFigure 3. Association of Patient Factors With Initial Postdose QTc Interval eFigure 4. CONSORT Diagram: Screening, Randomization, Follow-Up, and Analysis [file jamanetwopen-e2352034-s003.pdf]

Supplementary Online Content

Stollings JL, Boncyk CS, Birdrow CI, et al. Antipsychotics and the QTc interval during delirium in the intensive care unit: a secondary analysis of a randomized clinical trial. *JAMA Netw Open*. 2024;7(1):e2352034. doi:10.1001/jamanetworkopen.2023.52034

eTable 1. Daily Average Predose QTc Interval Across All Study Days

eTable 2. The Effect of Pharmacologic Medication With QTc Interval Changes by Sex

eFigure 1. Association Between ECG and Telemetry QTc Interval During Study Drug Held for QTc Prolongation

eFigure 2. The Distribution of Initial Postdose QTc Interval

eFigure 3. Association of Patient Factors With Initial Postdose QTc Interval

eFigure 4. CONSORT Diagram: Screening, Randomization, Follow-Up, and Analysis

This supplementary material has been provided by the authors to give readers additional information about their work.

© 2024 Stollings JL et al. *JAMA Network Open.*

eTable 1. Daily Average Predose QTc Interval Across All Study Days

	N	Placebo N=1009	Haloperidol N=962	Ziprasidone N=940	Combined N=2911
Day 1	557	442 (420.5-469.2)	447.8 (416.6-472.4)	444.8 (417.6-468)	445 (418-470)
Day 2	496	435.2 (413.1-472.2)	443.5 (412.5-472.8)	440 (413.5-465)	440 (413.5-470.2)
Day 3	407	442.5 (418.8-471)	442 (417-470)	444.5 (421-465)	444 (418.8-467.5)
Day 4	311	439 (410-465)	437.5 (409-470)	446 (416.2-470)	441 1(412.5-469)
Day 5	234	438 (404-462)	435 (405.8-457)	436.2 (409.1-455.6)	435 (405.1-460)
Day 6	190	432.5 (413.8-460)	435.2 (405.6-457.9)	440 (406.8-466.4)	436.5 (408.6-462.4)
Day 7	152	430.5 (402-452)	426.5 (404.5-448.2)	443.5 (409.2-462.6)	432 (405-453.6)
Day 8	116	433.2 (404.6-458)	429.5 (400-455.5)	454 (420-468)	438.2 (408.9-461.6)
Day 9	90	436 (407.5-468.5)	395.5 (375-428)	443 (426.6-456.6)	431.5 (402.8-450)
Day 10	80	442.5 (410-462)	404 (387.5-435.1)	445 (419-457.5)	429.8 (405.9-455.5)
Day 11	72	441 (411.2-463)	426.5 (402.5-441)	430 (413.2-468.2)	430.2 (405-460)
Day 12	61	439.5 (410.5-460)	420.8 (398-441.1)	424.8 (403-465.6)	434.5 (400-455.5)
Day 13	60	443.2 (427.9-467.9)	408.8 (383.9-440.4)	435.5 (403.8-452.2)	432.2 (405-456.8)
Day 14	49	439 (428.5-452)	420 (410-460)	450 (411-482)	436.5 (411-460)
Day 15	36	439.2 (397.4-466.8)	408 (394-437)	410 (408-436.5)	418 (398.5-459.2)

© 2024 Stollings JL et al. *JAMA Network Open.*

eTable 2. The effect of pharmacologic medication with QTc interval changes by sex

**Placebo**				
	**N**	**Male N=107**	**Female N=77**	**Combined N=184**
Baseline/pre-randomization QTc interval	184	450 (430-470.5)	453 (434-475)	452 (431.8-472.2)
Median of total dose per day (mg)	183	N/A	N/A	N/A
Days received study drug within the intervention period	183	4 (3-7.5)	4 (3-6.2)	4 (3-7)
Initial QTc change (among those who have post-dose QTc measured)	82	0 (-14-12)	3.5 (-11.2-10.8)	0.5 (-14-12)
Change in QTc from Day 1 to Day 2	170	-3 (-24-17)	-6 (-28-17)	-3.5 (-24.8-17)
Median QTc change during intervention period	170	0 (-8-6.5)	-1.5 (-10-7.5)	-0.2 (-10-6.9)
Peak QTc change during intervention period	170	30 (11-55)	25 (10-50)	29 (10.2-50)
**Haloperidol**				
	**N**	**Male N=108**	**Female N=84**	**Combined N=192**
Baseline/pre-randomization QTc interval	192	459 (432-479)	455 (432.8-480)	457.5 (432-479)
Median of total dose per day (mg)	192	11.9 (7.5-20)	10 (7.5-15.6)	10 (7.5-20)
Days received study drug within the intervention period	192	4 (3-6)	4 (2-6)	4 (3-6)
Initial QTc change (among those who have post-dose QTc measured)	86	8 (-13-21.5)	3 (-10-20.5)	6 (-10.8-21)
Change in QTc from Day 1 to Day 2	179	0 (-24-14)	-4.5 (-33-19.5)	-1 (-28-15)
Median QTc change during intervention period	179	0 (-14-6)	0 (-11-7.6)	0 (-13.5-6.5)
Peak QTc change during intervention period	179	24 (9-50)	23.5 (9.5-49.2)	24 (9-50)

© 2024 Stollings JL et al. *JAMA Network Open.*

**Ziprasidone**				
	**N**	**Male N=108**	**Female N=82**	**Combined N=190**
Baseline/pre-randomization QTc interval	190	453.5 (427.8-468.2)	448.5 (423.2-475.8)	451 (424.2-472)
Median of total dose per day (mg)	189	20 (11.2-30)	20 (10-30)	20 (10-30)
Days received study drug within the intervention period	189	4 (2-6)	4 (3-7)	4 (3-7)
Initial QTc change (among those who have post-dose QTc measured)	82	2 (-10-12.8)	6.5 (-9.8-19.2)	5 (-10-16.5)
Change in QTc from Day 1 to Day 2	171	-1.5 (-23.8-20)	0 (-20-19.5)	0 (-23-20)
Median QTc change during intervention period	171	0 (-9.6-8.6)	0 (-8.8-5.8)	0 (-9.5-7.2)
Peak QTc change during intervention period	171	26.5 (8-52.5)	27 (10-48.5)	27 (10-51.5)

Abbreviations: mg: milligrams

© 2024 Stollings JL et al. *JAMA Network Open.*

eFigure 1. Association Between ECG and Telemetry QTc Interval During Study Drug Held for QTc Prolongation

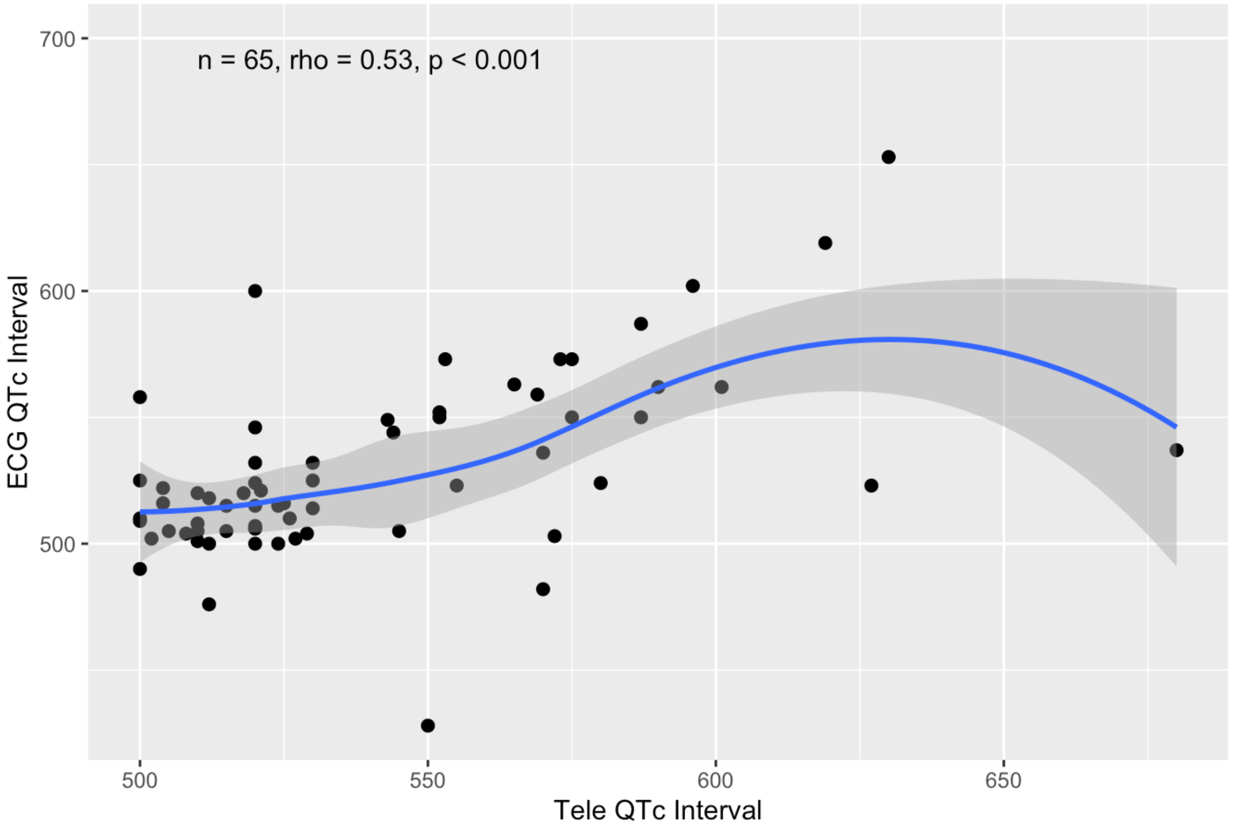

© 2024 Stollings JL et al. *JAMA Network Open.*

eFigure 2: The Distribution of Initial Postdose QTc Interval

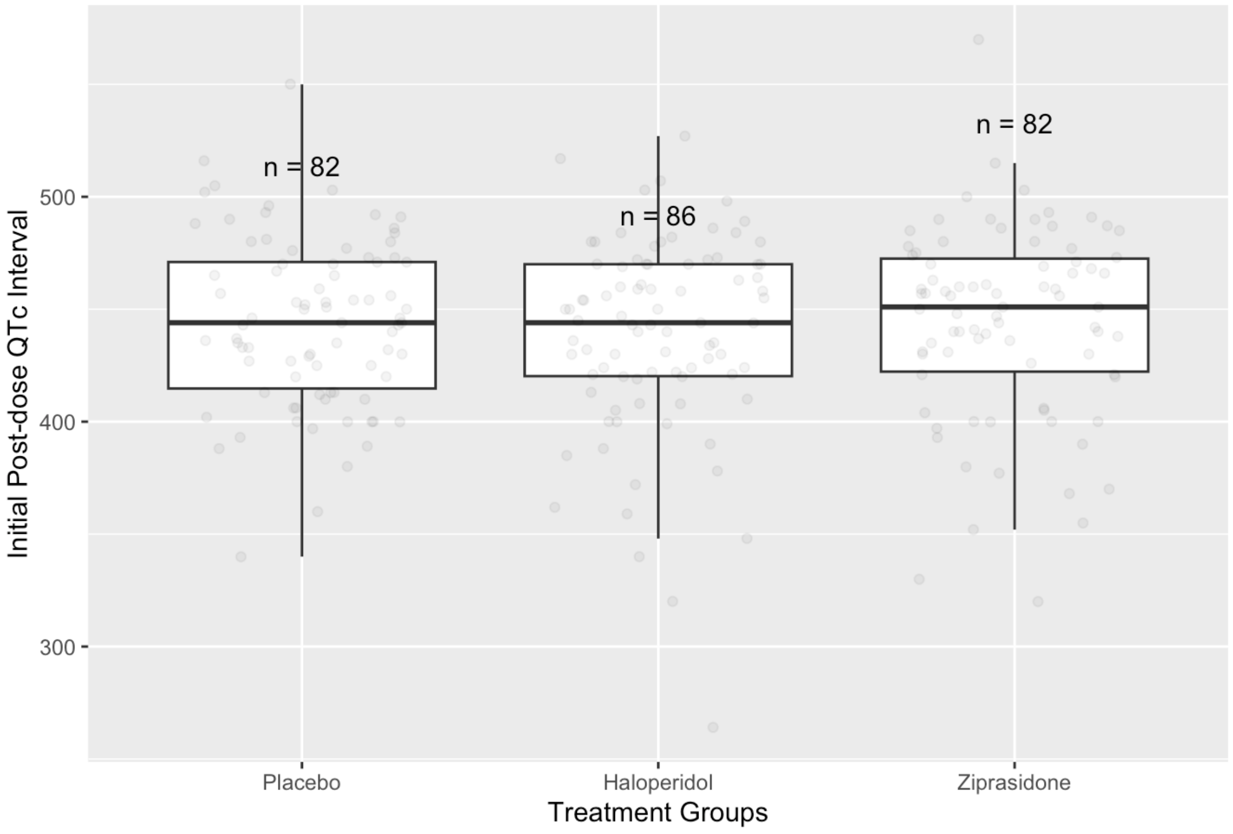

© 2024 Stollings JL et al. *JAMA Network Open.*

eFigure 3. Association of Patient Factors with Initial Postdose QTc Interval

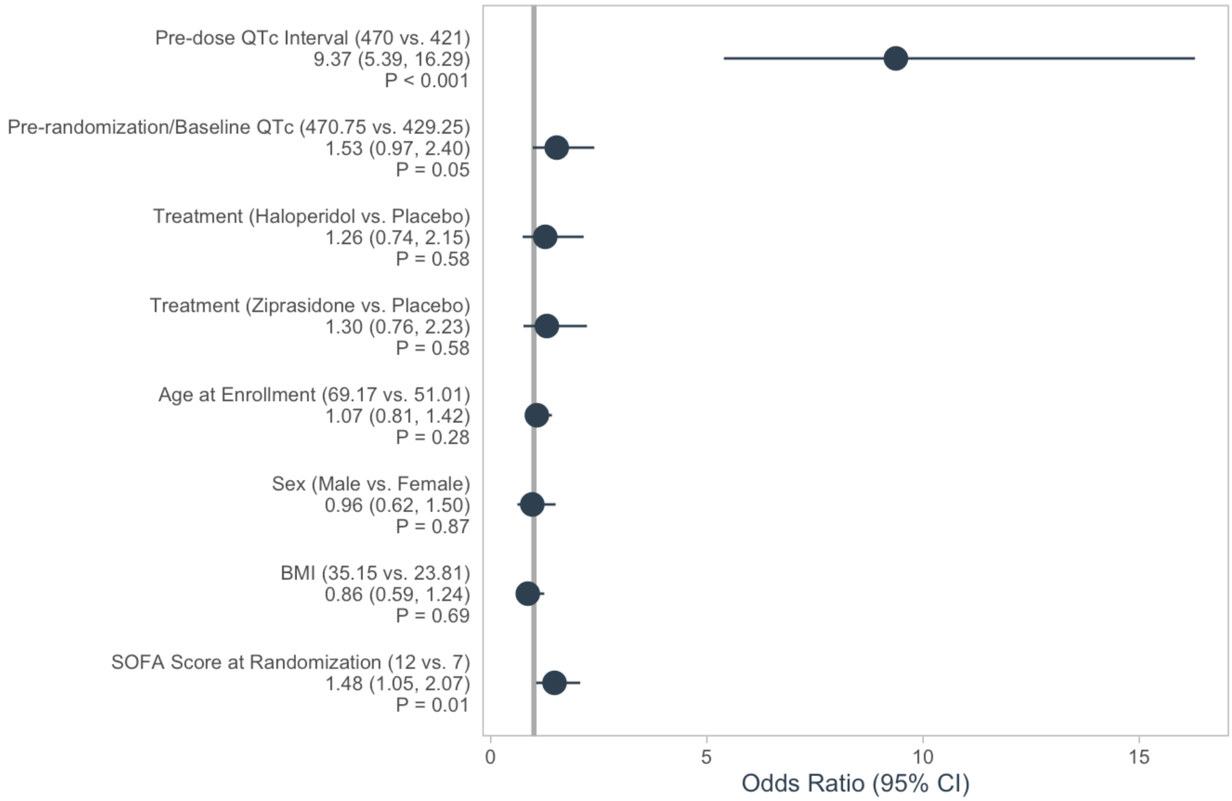

© 2024 Stollings JL et al. *JAMA Network Open.*

eFigure 4. Consort Diagram: Screening, Randomization, Follow-up, and Analysis

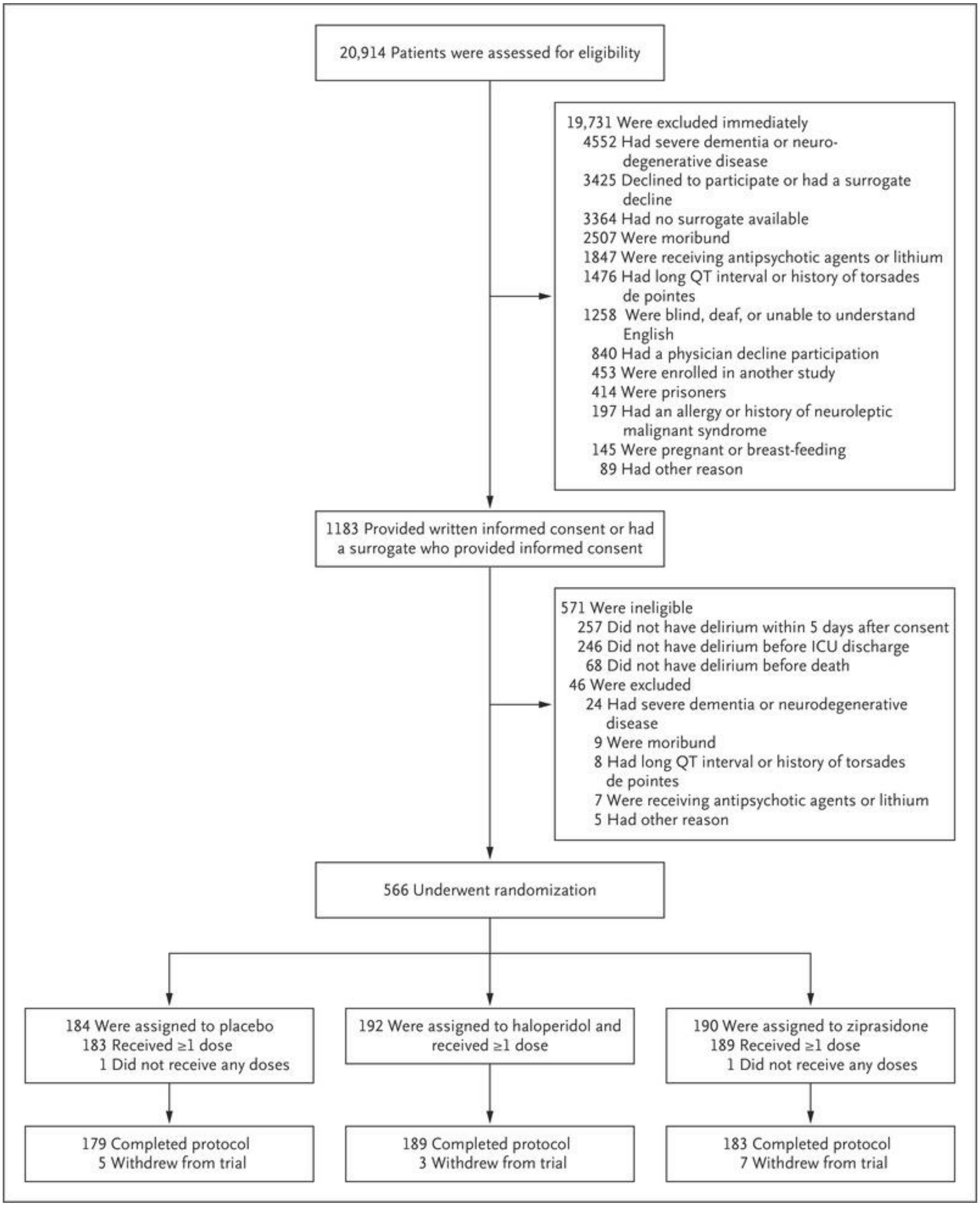

© 2024 Stollings JL et al. *JAMA Network Open.*
