# Supplementary material for: Antipsychotics and the QTc Interval During Delirium in the Intensive Care Unit: A Secondary Analysis of a Randomized Clinical Trial
Source: JAMA Netw Open. 2024 Jan 22;7(1):e2352034. doi: 10.1001/jamanetworkopen.2023.52034 (PMC10804270; doi:10.1001/jamanetworkopen.2023.52034)
Supplement: Supplement 4. — Data Sharing Statement [file jamanetwopen-e2352034-s004.pdf]

Data Sharing Statement

Stollings. Antipsychotics and the QTc Interval During Delirium in the Intensive Care Unit. *JAMA Netw Open*. Published January 22, 2024. doi:10.1001/jamanetworkopen.2023.52034

Data
Data available: No

Additional Information
Explanation for why data not available: as it arises, we're happy to supply supporting documents, provide access to our data, and collaborate with others for future analyses
